# Peeling back the many layers of competitive exclusion

**DOI:** 10.3389/fmicb.2024.1342887

**Published:** 2024-03-21

**Authors:** John J. Maurer, Ying Cheng, Adriana Pedroso, Kasey K. Thompson, Shamima Akter, Tiffany Kwan, Gota Morota, Sydney Kinstler, Steffen Porwollik, Michael McClelland, Jorge C. Escalante-Semerena, Margie D. Lee

**Affiliations:** ^1^School of Animal Sciences, College of Veterinary Medicine, Virginia Polytechnic Institute and State University, Blacksburg, VA, United States; ^2^Department of Population Health, University of Georgia, Athens, GA, United States; ^3^Department of Biomedical Sciences and Pathobiology, College of Veterinary Medicine, Virginia Polytechnic Institute and State University, Blacksburg, VA, United States; ^4^Department of Microbiology and Molecular Genetics, University of California, Irvine, Irvine, CA, United States; ^5^Department of Microbiology, University of Georgia, Athens, GA, United States

**Keywords:** pathogen, exclusion, *Salmonella*, antimicrobials, competition, attenuation

## Abstract

Baby chicks administered a fecal transplant from adult chickens are resistant to *Salmonella* colonization by competitive exclusion. A two-pronged approach was used to investigate the mechanism of this process. First, *Salmonella* response to an exclusive (*Salmonella* competitive exclusion product, Aviguard^®^) or permissive microbial community (chicken cecal contents from colonized birds containing 7.85 Log_10_*Salmonella* genomes/gram) was assessed *ex vivo* using a *S. typhimurium* reporter strain with fluorescent YFP and CFP gene fusions to *rrn* and *hilA* operon, respectively. Second, cecal transcriptome analysis was used to assess the cecal communities’ response to *Salmonella* in chickens with low (≤5.85 Log_10_ genomes/g) or high (≥6.00 Log_10_ genomes/g) *Salmonella* colonization. The *ex vivo* experiment revealed a reduction in *Salmonella* growth and *hilA* expression following co-culture with the exclusive community. The exclusive community also repressed *Salmonella*’s SPI-1 virulence genes and LPS modification, while the anti-virulence/inflammatory gene *avrA* was upregulated. *Salmonella* transcriptome analysis revealed significant metabolic disparities in *Salmonella* grown with the two different communities. Propanediol utilization and vitamin B12 synthesis were central to *Salmonella* metabolism co-cultured with either community, and mutations in propanediol and vitamin B12 metabolism altered *Salmonella* growth in the exclusive community. There were significant differences in the cecal community’s stress response to *Salmonella* colonization. Cecal community transcripts indicated that antimicrobials were central to the type of stress response detected in the low *Salmonella* abundance community, suggesting antagonism involved in *Salmonella* exclusion. This study indicates complex community interactions that modulate *Salmonella* metabolism and pathogenic behavior and reduce growth through antagonism may be key to exclusion.

## Introduction

Day-of-hatch chicks are susceptible to clinical disease in response to *Salmonella* exposure (Gast and Beard, 1989); however, chicks challenged at 2 days of age are easily colonized but do not exhibit clinical disease symptoms (Cheng et al., 2015). Young chicks’ intestinal microbiota contains low community diversity, with *Enterobacteriaceae* as a dominant bacterial group, indicating that the community may have a large niche for Gram-negative enterics (Lu et al., 2003; Ballou et al., 2016; Zhou et al., 2021). *Salmonella* colonization and *Enterobacteriaceae* abundance diminish with age as community diversity increases (Pedroso et al., 2021). Firmicutes and Actinomycetota become the abundant Gram-positive bacteria, while the Bacteroidetes, are the dominant Gram-negatives in the chicken intestinal microbiome as it matures (Lu et al., 2003; Xiao et al., 2017; Bhogoju et al., 2018; Khan et al., 2020). In the mature chicken, small intestinal and cecal intestinal compartments contain distinctly different communities (Lu et al., 2003; Choi et al., 2014; Xiao et al., 2017; Khan et al., 2020). This community segregation, however, does not occur until birds are approximately 3 weeks old (Lu et al., 2003), and at this time the abundance of intestinal *Salmonella* begins to decline (Cheng et al., 2015).

Day-of-hatch chicks exposed to a mature intestinal microbiota rapidly develop high community diversity (Pedroso et al., 2016) and are resistant to *Salmonella* infection and colonization (Nurmi and Rantala, 1973). One of the earliest examples of fecal transplants was done in chickens, where cecal bacteria from mature hens were shown to block *Salmonella* colonization in chicks (Nurmi and Rantala, 1973). This phenomenon was later termed competitive exclusion, even though it was not known whether competition (Fujikawa, 2016) was at the heart of the exclusion mechanism. Fecal transplants with the intestinal microbiota from adult chickens have also been shown to dramatically reduce *Salmonella* prevalence in broiler chickens in the field (Wierup et al., 1988; Hirn et al., 1992). Nurmi suggested that competitive exclusion may be attributed to: (1) competition for limiting nutrients; (2) competition for attachment sites on the mucosa; or (3) the production of antibacterial substances, including volatile fatty acids (VFAs) or bacteriocins (Nurmi et al., 1992). Another possibility is that competitive exclusion (4) modulates the virulence of the enteropathogens, reducing their ability to modify the intestinal environment or create a tissue reservoir to enable persistence (Watkins and Miller, 1983).

A great deal of culture-based and molecular studies have focused on identifying potential intestinal community members involved in pathogen exclusion. Most molecular studies have used a 16S census approach to characterize the intestinal microbiota (Stenkamp-Strahm et al., 2018; Pedroso et al., 2021; Valeris-Chacin et al., 2022; Falardeau et al., 2023). Candidate taxons have been identified that positively or negatively correlate with *Salmonella* abundance (Azcarate-Peril et al., 2018; Ding et al., 2021; Pedroso et al., 2021; Pottenger et al., 2023). However, the identified intestinal species have not been repeatedly encountered across studies, suggesting a multifactorial role (Pedroso et al., 2021). The key to pathogen exclusion likely involves a diverse microbial community exhibiting complex metabolic and inhibitory effects (Lone et al., 2013; Stanley et al., 2014; Zhang et al., 2015; Chopyk et al., 2016; Pedroso et al., 2021).

With the success of undefined, diverse intestinal communities in competitive exclusion, efforts led to the development of undefined intestinal communities free of any avian pathogens (Schneitz et al., 1991). Aviguard^®^ is a competitive exclusion product derived from the cecal contents of adult hens that has been shown to be effective at reducing *Salmonella* and other enteropathogens in the intestine of chickens (Hofacre et al., 1998, 2000; Weschka et al., 2021). This competitive exclusion product has also been shown to promote intestinal development in young chicks (Lee et al., 2023a). Undefined intestinal communities used as competitive exclusion products have been limited in their distribution or application by regulatory agencies like the U.S. Food and Drug Administration (Lee et al., 2023b). This has turned emphasis toward the development of single or multiple microbial species formulations capable of excluding an animal pathogen (Khan et al., 2020). These microbes have either been isolated from the intestine (Pascual et al., 1999; Kubasova et al., 2019), chosen based on similarities with intestinal species (Nair et al., 2021), or based on microbial properties of competition or antagonism so as to be detrimental to the pathogen in question (Latorre et al., 2016).

While the chicken small intestine is rich in nutrients, *Salmonella* and other Proteobacteria are a minor population within the bacterial community in this environment (Lu et al., 2003; Wang et al., 2022). In chickens, *Salmonella*’s niche appears to be the cecum (Snoeyenbos et al., 1982). While this compartment, in comparison to the small intestine, is scarce in free sugars (Józefiak et al., 2004), *Salmonella* abundance can reach 10^7^ cells per gram of cecal content (Cheng et al., 2015), despite the fact that the cecum expels its content daily. In general, Proteobacteria are not capable of degrading the fibers and mucin polysaccharides that are plentiful in the cecal compartment. They are therefore largely dependent on *Clostridia*, which are primary degraders capable of breaking down complex polysaccharides into free mono- and disaccharides that *Salmonella* and other scavengers can then metabolize. This scavenger activity would be able to occur through cooperation, in that members of the microbiota could provide nutrition for other members. However, the pathogen is in competition with other intestinal species for even these resources. One possible adaptation for this competition would be for *Salmonella* to metabolize fermentation end products produced by the primary degraders through respiration (Shelton et al., 2022). Their metabolism of these waste products would also help maintain an environment favorable to continued metabolism and growth for the primary degraders, illustrating another example of cooperation (Benoit et al., 2020). In ecological terms, intestinal member species can interact with *Salmonella* favorably or unfavorably through cooperation, competition, or antagonism (Hammarlund et al., 2021; Rogers et al., 2021). Some intestinal member species can only compete with *Salmonella* for the same limited resources by producing metabolites that suppress growth or kill their competitor through antagonism (Sibinelli-Sousa et al., 2022). Competitive exclusion therefore works by failing to support pathogen growth either through competition or antagonism, which has been the guiding principle of many groups in developing probiotics (Zhao et al., 2006; Maltby et al., 2013). Competition may only be effective under conditions where primary degraders and other member species stop feeding *Salmonella* nutrients that only it can metabolize, forcing it to compete for other resources equally metabolized by other intestinal community members.

Because the mechanism of competitive exclusion is currently unknown and is likely multifactorial, in this study, an *ex vivo* transcriptomic approach was used to reveal *Salmonella* physiology and behavior in response to permissive or exclusive communities. The *ex vivo* approach would allow direct analysis of *Salmonella* transcriptional response even if their growth was inhibited. In addition, chickens were experimentally infected with *Salmonella*, and its abundance was quantified in order to allow *in vivo* analysis of the cecal microbial transcriptomes to identify community metabolism that favors or excludes *Salmonella*. These experiments revealed significant metabolic differences in *Salmonella*’s response to the intestinal community and vice versa; however, failure to metabolize liberated sugars or microbial metabolites did not fully account for the mechanism of exclusion by the *Salmonella* exclusive community. The chicken intestinal community was found to reduce pathogen behavior (attenuation) and metabolism (cooperation/competition) and produce antimicrobials that limit *Salmonella* growth (antagonism), suggesting that the mechanism of competitive exclusion was multifactorial.

## Materials and methods

### Experimental approach

**Figure 1:**
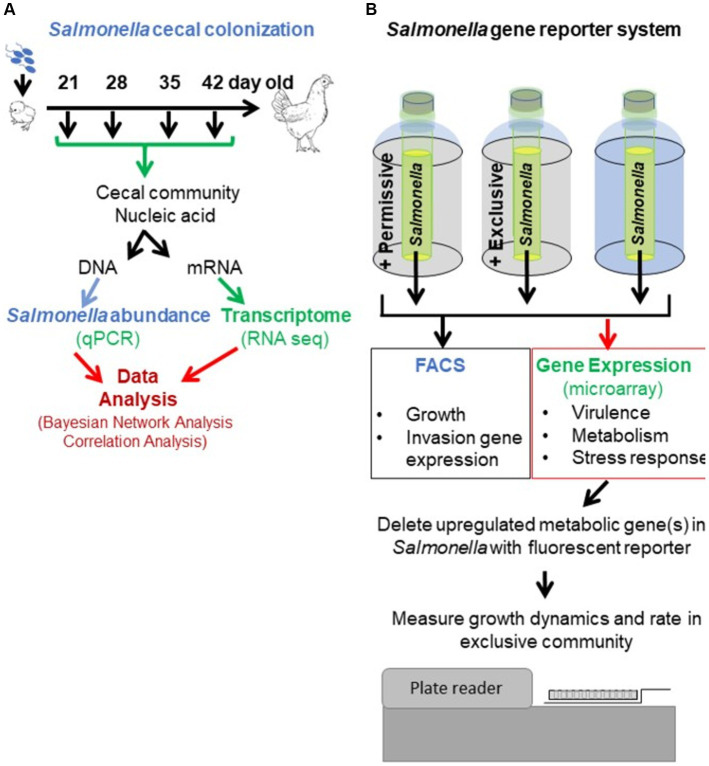
Experimental approach to revealing the mechanism of action of competitive exclusion. A two-prong approach involved determining cecal community metabolism relative to *Salmonella* abundance *in vivo* **(A)** and *Salmonella* expression in response to a permissive or exclusive community *ex vivo* **(B)**. In the *in vivo* study, 2-day-old, specific pathogen-free white leghorn chickens were orally administered *Salmonella* Typhimurium SL1344 (1 × 10^5^ CFU). At different ages, chickens were sacrificed, the ceca were aseptically collected from each bird, and nucleic acids were extracted. *Salmonella invA* qPCR was used to determine *Salmonella* abundance in the cecal community samples. The cecal community RNA was sequenced and annotated using the MG-RAST pipeline. The *ex vivo* study focused on *Salmonella* response when grown microaerophilically in a simulated cecal medium, *Ex Vivo* Cecal Contents (EVCC), containing a permissive or exclusive community. A fluorescent *S. typhimurium* SL1344 *rrn* promoter::*yfp*, *hilA* operon::*cfp* reporter strain was placed within dialysis tubing, physically separate from the permissive or exclusive community, enabling the collection of *Salmonella* cells for analysis. Jellyfish green fluorescent protein variants YFP and CFP served as reporters for *Salmonella* growth and SPI-1 invasion gene reporter, respectively. Fluorescence was measured using fluorescence-activated cell sorting. The cecal community from a 35-day-old chicken with high *Salmonella* abundance served as the permissive community, while the exclusive community consisted of the cecal community comprising the competitive exclusion product Aviguard^®^. *Salmonella* grown in EVCC alone served as a control. Select *Salmonella* metabolic genes, upregulated in either or both communities, were deleted in a *S. typhimurium* SL1344 fluorescent reporter strain, and mutants were compared to the wild-type reporter strain for growth in the exclusive community.

The mechanism underlying competitive exclusion involves one of the principles of population biology: cooperation, competition, antagonism, or attenuation. Table 1 describes the research approach to identifying the role of each in the competitive exclusion of *Salmonella*. One methodological challenge, addressed by this research approach, was detecting *Salmonella* growth rate and transcriptional signal in low abundance in the cecal microbiota, whose abundance may exceed *Salmonella*’s by 1,000-fold (Cheng et al., 2015). Therefore, a non-culture-based approach was used to determine cecal community metabolism correlating with *Salmonella* abundance *in vivo* in chickens (Figure 1A) and *Salmonella* gene expression and growth *ex vivo*, in response to communities that “permit” or “exclude” *Salmonella* (Figure 1B). The permissive community used in the *ex vivo* experiments was acquired from the *in vivo* chicken experiment and consisted of cecal contents from 35-day-old birds with high *Salmonella* abundance (7.86 Log_10_ *Salmonella* genomes/g cecal content) (Pedroso et al., 2021). The exclusive community used in the *ex vivo* experiments was the commercially competitive exclusion product, Aviguard^®^ (Lallemand Animal Nutrition; Montreal, Canada), which reduces *Salmonella* colonization in chickens by at least 5.0 Log_10_ CFU (Lee et al., 2023b).

**Table 1:** The mechanism underlying competitive exclusion.

Mechanism	*Salmonella* growth *in vivo*	Cecal community activities	Research approach	Expected outcomes
Cooperation	Dependent on community activity to provide nutritional resources	Provides essential resources for *high Salmonella* abundance	*Ex vivo* growth system (Figure 1A) Monitor *Salmonella* abundance and virulence expression in the presence of permissive or exclusive communities Monitor *Salmonella* expression by microarray analysis Monitor *Salmonella* mutants’ growth rates and growth dynamics *In vivo* chicken cecal colonization (Figure 1B) Monitor *Salmonella* abundance (qPCR) Perform cecal community transcriptomics	*Salmonella* growth and abundance will be higher in permissive vs. exclusive communities *Salmonella*’s catabolic gene expression will reflect growth metabolism from nutrients provided by a permissive community vs. starvation response resulting from attempts to replicate in an exclusive community Transcriptomes of cecal communities with high *Salmonella* abundance metabolically correlate with *ex vivo Salmonella* gene expression in the presence of a permissive community
Competition	Dependent on community metabolic activity to provide nutritional resources. However, the community also provides nutritional resources for multiple species of organisms	Provides essential resources but *Salmonella* abundance varies depending on abundance of competing species		Same Outcomes 1 and 2 for Cooperation Cecal transcriptomes will differ within birds with differing *Salmonella* abundance *Salmonella* metabolic mutants display growth defects within exclusive community
Antagonism	Dependent on community metabolic activity but abundance independent of nutritional resources	Provides essential resources varied *Salmonella* abundance Inhibitory behavior *low Salmonella* abundance		Same as Outcome 1 for Cooperation and Competition *Salmonella*’s expression of stress response reveals production of antimicrobials by the exclusive community Cecal communities’ transcriptomes contain transcripts for antimicrobial synthesis bacteriocins or other antibacterial mechanisms, for example, Type 6 Secretion System, indicating antimicrobial activity in communities with low *Salmonella* abundance
Attenuation	Dependent on community metabolic activity but invasion provides additional resources	Provides essential resources varied *Salmonella* abundance Oxidative stress *high Salmonella* abundance		No difference in *Salmonella* growth *ex vivo* with either community SPI-1 (invasion) repressed in *Salmonella* grown *ex vivo* with exclusive community Cecal transcriptome contains transcripts associated with production of attenuating molecules such as butyrate or indole

The *ex vivo* system was developed to monitor *Salmonella* growth, virulence (SPI-1 expression), and gene expression (microarray) in response to the permissive and exclusive communities. The jellyfish green fluorescent protein variants, yellow fluorescent protein (*yfp*) and cyan fluorescent protein (*cfp*), were fused to *rrn* growth-dependent promoter and *hilA* operon (SPI-1 cell invasion locus), respectively, in *Salmonella*. Fluorescence associated with the YFP and CFP reporters was used to monitor *Salmonella* growth and SPI-1 virulence gene expression in co-culture with cecal communities *ex vivo*. The *Salmonella* reporter strain was grown in dialysis tubing in a simulated cecal medium, *Ex Vivo* Cecal Contents (EVCC), submerged in permissive or exclusive communities to enable collection of *Salmonella* cells for study even if there was poor growth. Initially, the fluorescent reporters were used to empirically determine the earliest time point at which the exclusive community had the most significant impact on *Salmonella* growth or virulence expression relative to the permissive community. Six-hour co-culture of the reporter strain with the communities was determined based on YFP brightness measured by flow cytometry. Cells were then harvested for gene expression (microarray) comparisons, and genes within metabolic pathways that were differentially expressed in permissive vs. exclusive communities were deleted in *Salmonella* using pGLOW, a fluorescent reporter visible in anaerobic conditions. *Salmonella* mutants’ growth dynamics when co-cultured with the exclusive community were monitored continuously over 48 h using a fluorescence plate reader.

Table 1 also describes expected outcomes based on the roles of cooperation, competition, antagonism, or attenuation in the mechanism of *Salmonella* competitive exclusion with respect to the *in vivo* and *ex vivo* community transcriptomics and *Salmonella* response. The detailed methodology for each research approach is described in detail below (*in vivo*: “The cecal communities’ transcriptome relative to *Salmonella* abundance”; *ex vivo*: “Assessing *Salmonella* growth rate and SPI-1 T3SS expression in an *ex vivo* system with permissive or exclusive communities,” “Microarray analysis of *S.* Typhimurium YC 1104 reporter strain growth in permissive or exclusive microbial communities,” and “Contribution of metabolic gene(s) to *Salmonella* Typhimurium growth in an exclusive community”).

### Construction of *Salmonella* reporter strains and mutants

λ Red (Datsenko and Wanner, 2000) was used to construct *rrn* and *iag* (SPI-1) reporters, tagged with jellyfish green fluorescent protein variants: yellow fluorescent protein (YFP) and cyan fluorescent protein (CFP), respectively (Heim and Tsien, 1996; Miyashiro and Goulian, 2007). The growth-dependent *rrn* promoter was cloned upstream of a “promoter-less” *yfp* in tandem with the chloramphenicol resistance marker *cat* (Supplementary Tables S1, S2). The *rrnB* P1 promoter was engineered into the forward primer targeting 5′ *yfp* with its ribosome binding site (RBS) (Supplementary Table S2). The reverse primer was initially designed with a 3′ overlap with *yfp* and the *rrnB*T12 transcriptional terminator, previously cloned downstream of *yfp* in plasmid pMG32 to create pMG32T1T2 (Supplementary Tables S1, S2). These primers, using pMG32T1T2 as template, were used to amplify *yfp*, now with *rrnB* P1 promoter and T1T2 transcriptional terminators. The resulting amplicon was cloned into pCR-XL-TOPO and subsequently subcloned into Π-dependent suicide vector pGP704 (Miller and Mekalanos, 1988) to create pCY01 (Supplementary Figure S1). The *rrnB*T1T1 was replaced with λT0 transcriptional terminator (pCY02), to which *cat* was introduced 3′ from this terminator by cloning amplicons with engineered restriction enzyme sites for directional cloning to generate the final plasmid pCY03 (Supplementary Tables S1, S2). PCR primers were designed to target a P22 insertion site, an intergenic site between *thrW* and STM0324, and contained overlap with the *rrn* promoter and *cat* sequences. These primers were used to amplify the reporter construct using pCY03 as template (Supplementary Figure S1; Supplementary Table S2). λ Red was used to introduce *Salmonella* growth reporter into a P22 integration site with the P22*att*-*rrnB* P1-*yfb*-*cat* amplicon (Datsenko and Wanner, 2000).

The *hilA* operon reporter using *cfp* was constructed as follows. Tetracycline resistance gene *tetA* was first introduced into the intergenic region between *iagB* and *sptP*, just 5′ of the transcriptional terminator for the *hilA* operon, using λ Red (Datsenko and Wanner, 2000), with primers described in Supplementary Table S2 and pKD3 to generate the amplicon used to transform *S. typhimurium* SL1344. *iagB* is the last gene in the *hilA* operon. The *tetA* was later replaced with a “promoter-less” *cfp* following transformation with the *cfp* amplicon generated with *iagB*, *sptB*, and *cfp* primers (Supplementary Table S2) and selection for fusaric acid resistance. Fusaric-resistant colonies were screened for tetracycline sensitivity (Bochner et al., 1980). P22 was used to move *rrnB* P1*-yfp*-*cat* into *Salmonella* strain with *hilA* operon-*cfp* reporter to construct the final *S. typhimurium* strain YC1104 with *yfp* growth and *cfp*, *hilA* operon reporters (Provence and Curtiss Iii, 1994).

λ Red was also used to create single and double mutations in metabolic pathways in *Salmonella* (Supplementary Tables S1, S2). P22 generalized transduction was used to introduce λ*red*-generated knockouts with intact antibiotic resistance gene cassettes into *S. typhimurium* SL1344 or SL1344 mutants before removing the antibiotic resistance marker with pCP20. YFP was replaced with pGLOW as a reporter of *Salmonella* growth (evocatal GmbH, Monheim am Rhein, Germany). This reporter was chosen because fluorescence is not oxygen-dependent (Drepper et al., 2007), which allows monitoring of *Salmonella* growth continuously under strictly anaerobic conditions. Electroporation was used to introduce plasmids into *Salmonella* strains (Dower et al., 1988).

### Measuring YFP and CFP expression using fluorescence-activated cell sorting

Flow cytometry was conducted with a CyAn ADP Analyzer (Beckman Coulter; Brea, CA) using the software Summit (ver. 4.3, Beckman Coulter). The 468 nm excitation and FL1 530/30 BP filter were used for YFP detection. The 409 nm excitation and FL6 450/50 BP filter were used for detecting CFP. For each sample, a total of 20,000 events were collected. In some instances, samples were read multiple times to detect variations between sample collections. The flow cytometer reported fluorescence as median intensity values of arbitrary units. The cytometer flow rate was adjusted to keep the average event rate between 1,000 and 3,000 events per second to avoid coincidental detection of bacterial cells. Data were analyzed using FlowJo software (ver. 9.4, TreeStar, Inc.). Particles that did not fall into the expected size and shape of a bacterial cell were not included in the analyses. Positive (YFP, CFP; *Escherichia coli* with pMG32 or pMG34, respectively) and negative (*S. typhimurium* SL1344, wild-type strain) controls were included with every run.

### Assessing *Salmonella* growth and SPI-1 T3SS expression in an *ex vivo* system with permissive or exclusive communities

The *ex vivo* study entailed the growth of *Salmonella* reporter strain YC1104 in the presence of a permissive or exclusive community. The *S. typhimurium* reporter strain has *rrn-yfp* and *hilA* operon-*cfp* chromosomal promoter fusions for monitoring growth and SPI-1 expression, respectively. This approach allowed for growth of *Salmonella* to cell densities and volumes to later harvest sufficient *Salmonella* mRNA for microarray analysis. The reporter strain is a derivative of *S. typhimurium* SL1344, the same wild-type strain used in the *in vivo* study (see below).

A medium was developed to mimic the luminal cecal nutritional environment, based on published literature, and contained porcine gastric mucin, uric acid, six amino acids (arginine, cysteine, isoleucine, lysine, methionine, and threonine), and phytone peptone. All amino acids, except cysteine, were at concentrations previously reported to be found in the chicken cecum (Babinszky et al., 2006). Uric acid was added at a concentration found in the cecal contents of white leghorn chickens (Beck and Chang, 1980). A redox indicator, resazurin, and a reducing agent, cysteine, were also added at concentrations to enhance anaerobiosis (Demain and Solomon, 1986). This *ex vivo* chicken cecal (EVCC) medium formulation is described in Supplementary Table S3. Stock solutions were made for resazurin (1,000×), hemin (1,000×), uric acid (100×, pH 8.8–9.0), and amino acid supplement (10×, pH 7). The pH of the basal medium was adjusted to 6.1 ± 0.1 prior to autoclaving. After the addition of uric acid and amino acid supplements, the final pH of the EVCC medium was approximately 6.5.

All cultures, media preparation, and dilutions were performed in an anaerobic glove box. Media and diluent were sparged with gas to make conditions microaerophilic. Autoclaved, 30-ml-capacity serum bottles (Fisher Scientific) with rubber stoppers served as culture vessels. About 20 mL of EVCC medium (Supplementary Table S3) was added to each vessel in an anaerobic cabinet, to which a *Salmonella* permissive (7.86 Log_10_ *Salmonella* genomes/g cecal contents) or exclusive community was added (Supplementary Figure S2). One culture vessel was left uninoculated (EVCC alone) (Supplementary Figure S2). The lyophilized Aviguard^®^ product was rehydrated in sterile saline according to the manufacturer’s recommendation. This product is generally administered to chickens in drinking water and has been shown to reduce *Salmonella* colonization by at least 5 Log_10_ in chickens (Lee et al., 2023b). Direct bacterial cell counts were determined microscopically at 1,000× magnification using a Petroff-Hausser counting chamber. The cell density of the starting material was estimated for both communities at 10^11^ cells/ml. The rehydrated Aviguard viability was determined by a LIVE/DEAD BacLight stain (Molecular Probes; Grand Island, NY). The viability of the commercial lots varied between 30 and 75%. Both communities were diluted 100-fold in sterile saline before adding 2 mL of this cell suspension to EVCC medium to generate a final cell density of 10^8^ cells/ml. Spectrum™ (Fisher Scientific) cellulose ester membrane with 100,000 Daltons exclusion limit and 20 mm width served as the internal culture vessel for the *Salmonella* reporter. Approximately 80 mm of dialysis tubing was cut, rehydrated in sterile H_2_O, and clamped at one end with a dialysis clip, pre-treated with 70% ethanol. The clamped end was placed in a serum bottle first, and the open dialysis end was pulled over the mouth of the bottle. The *Salmonella* reporter strain was prepared as follows. Strain YC1104 was grown anaerobically on MacConkey agar at 39°C for 48 h. A cell suspension was made from growth on the MacConkey agar plate in sterile saline to 0.2 OD (λ 600 nm) (~10^8^ CFU/mL). The cell suspension was diluted 100-fold in sterile saline. Subsequently, 1 mL of the 100-fold dilution was used to inoculate 5 mL of EVCC medium (10^5^ CFU/mL final cell density), which was transferred to the dialysis tubing. A rubber stopper and metal crimp were used to seal the vessel. A sterile syringe needle was used to sparge the vessel with a gas mix of 6% CO_2_, 6% O_2_, 85% N_2_, and 3% H_2_ or sample the dialysis tubing as depicted (Supplementary Figure S2). The oxygen concentration was chosen based on jellyfish green fluorescent proteins and its variants’ requirements for oxygen to fluoresce (Heim et al., 1994) and the likely minimal oxygen tension necessary for supporting the microaerophile *Campylobacter jejuni* (Pennie et al., 1984), an abundant (8 Log_10_ CFU/g) bacterial species in the chicken cecum (Hue et al., 2011). The top was sealed with tape. Culture vessels were placed in an anaerobic jar, flushed with the same gas mix described previously, and incubated at 39°C to simulate the chicken’s internal body temperature. This and subsequent samplings (see below) were all done within an anaerobe glove box. The dialysis tubing’s exclusion limit allowed free exchange of metabolites, peptides, and proteins between *Salmonella* and the external microbial community. Due to the nature of the cellulose dialysis material and because the cecal anaerobic community may secrete cellulases, the integrity of the membrane during experimentation was determined earlier by incubating tubing containing just dextran blue with a cecal community grown anaerobically in EVCC for 24 h at 39°C. There was no visible leakage of the dextran blue into the external culture, confirming the integrity of the dialysis membrane. Culture vessels were incubated at 39°C. The *Salmonella* reporter strain was periodically sampled over time (3, 6, 9, 12, and 24 h) using a sterile needle and syringe to sample the dialysis tubing, and fluorescence was monitored by fluorescence-activated cell sorting (FACS) to empirically identify the best time point, for harvesting *Salmonella*, where growth or SPI-1 expression was significantly reduced in the exclusive community, compared to the permissive community and the uninoculated, EVCC control. 0.2 mL aliquots were added to cryotubes containing 40 μL of 60% sterile glycerol (12% final concentration) and stored at −80°C for later FACS analysis.

### Microarray analysis of *Salmonella typhimurium* YC 1104 strain growth in permissive or exclusive microbial communities

Microarray analysis was used to assess *Salmonella*’s transcriptome response to microbial communities in the *ex vivo* system, as previously described, with the following modifications. *Salmonella* strain YC1104 was grown anaerobically on MacConkey agar at 39°C for 48 h. A cell suspension was made in 5-ml sterile saline from the MacConkey plate, adjusted to a cell density of 0.2 OD (λ 600 nm) (~10^8^ CFU/mL). This cell suspension was used to inoculate 5 mL of EVCC medium (10^6^ CFU/mL) and incubate anaerobically at 39°C for 12 h. Five milliliter of EVCC was inoculated with a 4-fold dilution of the 12 h culture to 10^7^ CFU/mL and transferred to dialysis tubing. The higher cell density per dialysis volume was needed to provide a sufficient amount of *Salmonella* RNA for microarray analysis. This experiment was done in triplicate, where *Salmonella* was grown and harvested after 6-h incubation in EVCC alone or in EVCC with the permissive or exclusive community (Supplementary Figure S2). *Salmonella* grown in culture vessels with EVCC alone served as the comparison control in the microarray analysis. Total RNA was harvested from *Salmonella* grown in the dialysis tubing, and microarray analysis was performed as previously described (Cheng et al., 2015). Briefly, the samples were treated with 0.1 mL volume of 95% ethanol and 5% acidic phenol (pH 4.3), and total RNA was extracted using MasterPure Complete DNA and RNA purification kits (Epicentra; Madison, WI) and High Pure RNA isolation kit (Roche; Indianapolis, IN). The samples were subsequently treated two times with the Turbo DNA-free kit (Ambion; Austin, TX). *Salmonella*-specific *hilA* PCR (Cheng et al., 2015) was used to confirm that RNA was free of contaminating DNA, and the quality and integrity of the RNA were evaluated by gel electrophoresis (Sambrook et al., 1989). Genomic *Salmonella* DNA served as a labeling and hybridization reference control. Hybridization and fluorescent labeling of RNA were performed as described by Fink et al. (2007). Microarray chips were scanned with a ScanArray 5,000 laser scanner (GSI Lumonics; Watertown, MA). The data were analyzed using QuantArray v.2.01 software, and background intensity was subtracted from spot boundary signal intensities to derive Cy3 and Cy5 median signal intensities. Differential gene expression and statistical significance were determined with the Significance Analysis of Microarrays software package (Stanford University).^1^ The data were analyzed using paired Student’s *t*-test, and statistical significance from multiple comparisons was determined by one-way analysis of variance followed by Bonferroni posttest, with statistically significant *p*-values set at <0.001.

### Contribution of metabolic gene(s) to *Salmonella* Typhimurium growth in an exclusive community

Mutant or wild-type strains, with the pGLOW reporter, were grown in EVCC + Aviguard. Oxyrase (Sigma-Aldrich; St. Louis, MO) was added to the medium (1:20 final dilution) and overlaid with mineral oil to create low oxygen conditions. *Salmonella* starting cell density was 10^5^ CFU/mL. The lyophilized Aviguard was reconstituted as recommended by the manufacturer (Lallemand Animal Nutrition) in sterile saline with Oxyrase and used to inoculate EVCC cecal medium (1:20 dilution) (7.99 Log_10_ viable cells/mL). The viability of the rehydrated Aviguard was determined microscopically using LIVE/DEAD BacLight stain (Molecular Probes). Sterile 96-well, black-walled polystyrene microtiter plates with a clear bottom (Fisher Scientific) were used to monitor *Salmonella* fluorescence with a BioTek Synergy HT 96-well fluorescence microtiter plate reader at 37°C. A filter was added to the BioTek Synergy HT fluorescent plate reader (BioTek; Winooski, VT) to record pGLOW fluorescence based on its excitation frequency (Drepper et al., 2007). Fluorescence was recorded every 30 min. Strains were run in triplicate.

### The cecal communities’ transcriptome relative to *Salmonella* abundance

The *in vivo* study involved oral administration of *Salmonella* Typhimurium SL1344 (1 × 10^5^ CFU) to 2-day-old, specific pathogen-free (certified *Salmonella*-free), white leghorn chickens (Charles River Laboratories; Wilmington, MA; *n* = 100), dispersed into five HEPA-filtered isolator units with wire mesh floors to reduce re-exposure due to coprophagy. To ensure birds were *Salmonella*-free, day-of-hatch chicks and their isolator environment were also tested for *Salmonella* by enrichment (Pedroso et al., 2021). Chicks and their environment were culture-negative for *Salmonella*. The birds were fed a non-medicated, commercial starter feed *ad libitum*. The bird density per isolator unit was reflective of commercial standards; culling birds was necessary periodically to maintain this stocking density. No probiotic(s), competitive exclusion product, or cocktail of intestinal bacterial species was administered to chickens in this study. One bird from each of five isolator units (*n* = 5) was collected at 21, 28, 35, and 42 days of age, euthanized, and the ceca was aseptically obtained. One bird in isolator 5 died after day 35, and therefore, there were only four subjects for day 42. *Salmonella invA* qPCR was used to enumerate *Salmonella* present in cecal DNA as previously described (Pedroso et al., 2021). Nucleic acid was extracted from cecal bacteria using the Mo Bio Soil DNA extraction kit, vortexing samples with glass beads at maximum speed for 40 min (Lu et al., 2003). Lysate was treated with 0.5% SDS and proteinase K (0.1 mg/mL) at 37°C for 30 min before phenol–chloroform–isoamyl alcohol (25:24:1) extraction. Sample nucleic acid was portioned equally, one being stored at −80°C for RNA purification (see below) and the other treated with DNAse-free RNase for *Salmonella* qPCR.

RNAse-free DNAse was added to nucleic acid (Pedroso et al., 2021) for each sample (*n* = 19) and incubated at 37°C. The quality and quantity of RNA were assessed by agarose gel electrophoresis. PCR, using universal 16S rRNA primers (Supplementary Table S2; Suzuki et al., 2000), assessed and confirmed that the RNA was free of DNA. Nucleic acid preps were repeatedly treated with RNAse-free DNAse until no PCR amplicon was detected to confirm RNA purity. Ribosomal RNA was removed using the MICROBExpress™ Additional rRNA removal was performed until samples were free of the detectable 23S and 16S rRNA bands (2.9 and 1.5 Kb, respectively) on an agarose gel. Finally, the Bacterial mRNA Enrichment Kit (Thermo Fisher Scientific; Waltham, MA) was used to further purify and concentrate cecal community mRNA, as described by the manufacturer’s instructions. mRNA was submitted to the Georgia Genomics and Bioinformatics Core for sequencing using RNA-seq (Illumina; San Diego, CA). The resulting sequence reads were uploaded to the MG-RAST server for annotation (Keegan et al., 2016).

### Carbohydrate and volatile fatty acid analysis of the chicken cecum

In order to reveal carbohydrates present in the cecal glycome of a permissive community, two samples with high (7.20 Log_10_ *Salmonella* genomes/g) vs. low *Salmonella* abundance (5.85 Log_10_ *Salmonella* genomes/g) were submitted to the University of Georgia Complex Carbohydrate Center for glycome analysis. Glycosyl composition analysis was performed by combined gas chromatography–mass spectrometry (GC–MS) of the per-O-trimethylsilyl (TMS) derivatives of the monosaccharide methyl glycosides produced by acidic methanolysis as previously described (Santander et al., 2013). GC–MS analysis of the resulting TMS methyl glycosides was performed on an Agilent 7890A GC interfaced to a 5975C MSD (Agilent; Santa Clara, CA), using a Supelco Equity-1 fused silica capillary column (30 mm × 0.25 mm, internal diameter).

Because some short-chain fatty acids can attenuate *Salmonella* virulence, inhibit, or promote pathogen growth (Kwon and Ricke, 1999; Zhang S. et al., 2020; Zhang Z.J. et al., 2020), cecal contents were obtained from two birds with high *Salmonella* abundance (7.00 Log_10_ *Salmonella* genomes/g cecal contents, 8.51 Log_10_ *Salmonella* genomes/g cecal contents). VFA analysis was performed by combined gas chromatography–mass spectrometry (GC–MS) of the butyl ester derivatives produced from the samples. The cecal contents were lyophilized and subsequently suspended in hexane, to which octadecane was added as an internal standard. Butyl esters were prepared by adding BF_3_-1-butanol to the sample and heating it at 100°C for 2 h in a sealed tube. The reaction was subsequently quenched with 1–2 mL H_2_O. The aqueous layer was discarded, and the organic phase was washed once with an equal volume of H_2_O. The organic layer was then used for GC–MS analysis, performed using HP 6890 GC (Agilent) interfaced to a 5975b MSD (Agilent) using an All Tech EC-1 fused silica capillary column (30 mm × 0.25 mm) (Fisher Scientific). A mixture containing known concentrations of VFAs and octadecane was treated under the same conditions and served as standards for quantification.

### Network and statistical analyses of chicken cecal meta-transcriptomes for cecal communities with high or low *Salmonella* abundance

Chicken cecum transcriptome read counts were normalized as a percentage of total reads for the MG-RAST categories KO metabolism, COG metabolism, virulence, and stress response. Enzymes were mapped and counted in the raw data set for sequence reads through Python version 3.7 (Ekmekci et al., 2016). These counted reads were merged in R through scripting, and then calculated as the percentage of these reads against total reads in Python to create a data set with normalized read counts of all enzymes in all samples. The read count data were edited, transposed, combined with *Salmonella* abundance, associated with individual samples, and filtered according to abundance from highest to lowest. The mean value of *Salmonella* abundance was calculated, and the value above the mean (5.85 Log_10_ *Salmonella* genomes/g cecal contents) was considered high abundance; a value equal to or below the mean was considered low abundance.

For analysis of the fermentation meta-transcriptome, the data set was first limited to 45 enzymes associated with fermentation in the KO data set, listed in Supplementary Table S5. This data set was expanded to 164 enzyme transcripts to include the enzymes listed in Supplementary Table S6. In the analysis of the microbiome’s stress response, data were pulled from stress response and virulence data sets and categorized into groups based on function ascribed to the enzyme, for example, DnaK and heat shock, and consolidated into the individual categories. Two hundred thirty-four enzymes extracted from these data sets included those associated with the following categories: heat shock; carbon starvation; extra-cytoplasmic/envelop stress response; regulation; translation and protein export; oxidative stress; osmotic shock; acid tolerance; iron metabolism; antimicrobials; miscellaneous; and polyketide synthesis (Supplementary Table S7).

Bayesian network analysis was performed in R with the bnlearn package (Friedman et al., 2013; Scutari and Denis, 2021). Bayesian network analysis is a probabilistic model in which a graph structure represents a qualitative dependency relationship among random variables and a conditional probability expresses a quantitative link between individual variables. This method is comparable to approaches used to infer gene regulatory networks based on microarray or RNA-seq data and represents a directed edge connecting two genes used to determine a biochemical process such as a reaction, transformation, interaction, activation, or inhibition. The arc or arrows represent the probability of connectivity. A graph structure showing dependency relationships between nodes, obtained from a collection of conditional probabilities, defines the model (Ebana and Furukawa, 2019). The conditional probability with the state of the node close to the arrow propagates through the arrow one after the other. The probability of each node is used to generate the graph structure. The effective final network produced automatically from the data indicates the impact and the association link between the data. The strength of probabilistic links, reflected by the arc of a connection, was measured and presented.

Several different network models were used, including the score-based learning algorithm—Hill-Climbing (HC) (Friedman et al., 2013); constraint-based structure learning algorithms—Max–Min Parents and Children (MMPC) (Lagani and Tsamardinos, 2010); hybrid structure learning algorithms—Max–Min Hill-Climbing (MMHC) (Song et al., 2022), Hybrid Parents and Children (H2PC) (Anonymous, 2021), and General 2-Phase Restricted Maximization (RSMAX2) (Yu et al., 2019) to identify a common network structure for the various enzymes or categories described. Among all these implemented algorithms, HC performed best and produced the highest number of connections with various data sets. Therefore, for model fitting, a network structure was inferred using the Hill-Climbing approach, and the quality of the fit was evaluated using the Bayesian information criterion (BIC). The HC algorithm trains the network in a score-based “greedy search” (Scutari et al., 2019). This algorithm ranks network architectures based on the increase/decrease in BIC score induced by the removal of the arc. The network score was used to build arcs connecting two nodes if there were direct relationships between them. The arc strength value >0.5 was considered reliable. Illogical arcs were “blacklisted” and excluded from analysis to improve model fitting and avoid circular structure in the structure learning process (Ebana and Furukawa, 2019).

A more detailed analysis was performed at the individual enzyme level for those enzymes associated with carbohydrate metabolism, fermentation/respiration, antimicrobials, and stress response (Supplemental Cecal Transcriptome Data Set; *n* = 598). Pearson and Spearman-rank correlations were determined for enzyme transcript abundance compared to *Salmonella* abundance. Significance was given to Pearson or Spearman r or ρ values >0.6999 or < −0.6999 and *p*-values <0.05.

The identity of enzyme transcripts, with network connections or statistically significant correlation with *Salmonella* abundance, was determined by BLAST search at the nucleotide level (Altschul et al., 1990). Species or genus were assigned to the enzyme transcript if there was ≥99% coverage and 98–100% sequence match. Some matches based on these criteria identified *Clostridia* yet to be classified, which were assigned strain name or descriptor of the matching deposited sequence or genome.

## Results

### *Salmonella* growth and expression of virulence genes were reduced in the presence of an exclusive community

**Figure 2:**
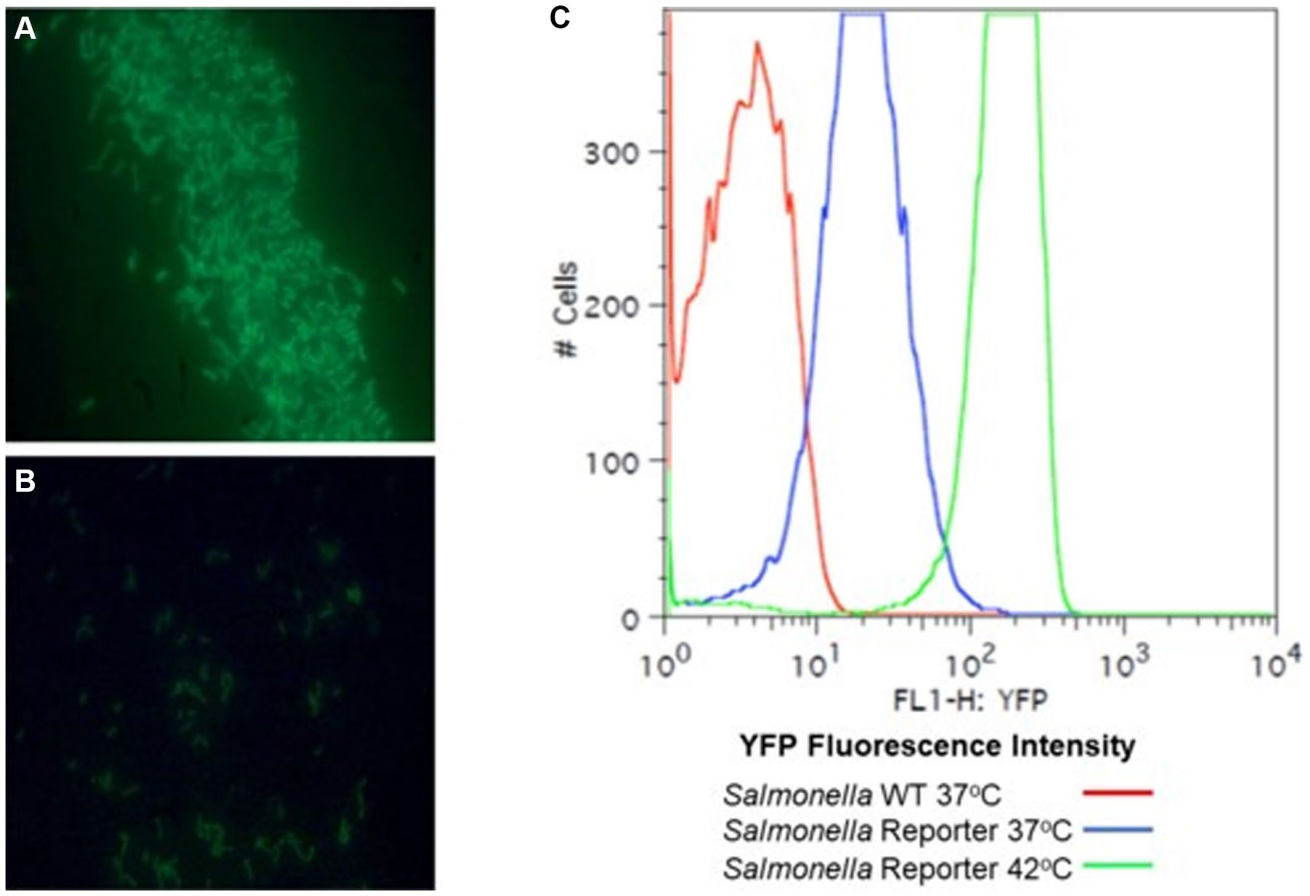
A *rrn* promoter jellyfish yellow fluorescent protein (YFP) gene fusion as a reporter for *Salmonella* growth. Lambda red was used to construct *rrn* tagged with YFP. The *rrn* promoter was cloned upstream of a “promoter-less” *yfp* and in tandem with *cat*. PCR primers were designed to overlap with the target insertion site, an intergenic site between *thrW* and STM0324. The λ Red system (Datsenko and Wanner, 2000) was used to introduce this reporter into *S. enterica* Typhimurium SL1344. *S. typhimurium* SL1344 strain YC1104 with the *rrn*-*yfp* reporter was grown aerobically to OD 1.0, λ 600 nm, in LB broth **(A)** or M9 minimal medium with 0.4% glycerol as a carbon source **(B)** with aeration and observed with a fluorescence microscope. **(C)** Fluorescence-activated cell sorting (FACS) analysis of the *Salmonella rrn*-*yfp* reporter strain grown aerobically, in LB broth to mid-exponential phase (OD 0.5, λ 600 nm) at 37°C or 42°C to demonstrate fluorescence intensity with a high growth rate.

**Figure 3:**
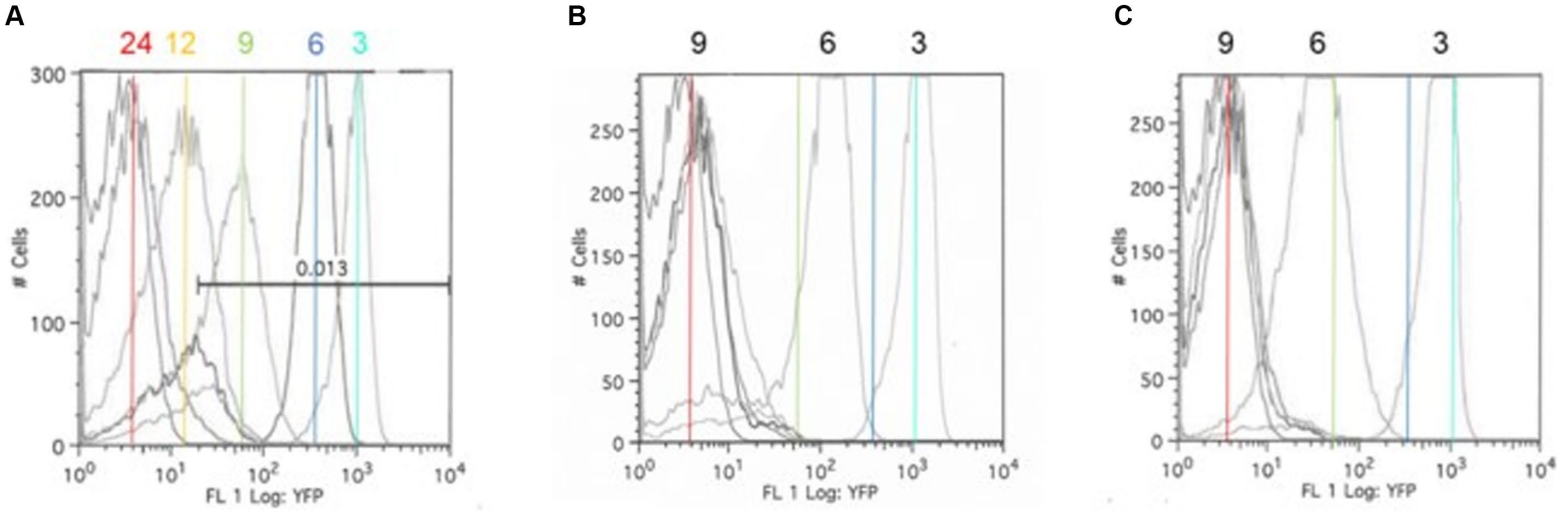
The *Salmonella ex vivo* growth in permissive **(B)** or exclusive **(C)** communities. FACS analysis was used to measure the *rrn-yfp* promoter activity in *S. typhimurium* SL1344 grown in simulated cecal medium (EVCC) **(A)** within a permissive or exclusive community. *Salmonella* YFP expression was monitored by FACS analysis at 3, 6, 9, 12, and 24 h using the parental strain as a negative fluorescence control. Cyan (3 h), blue (6 h), green (9 h), and red (24 h) lines mark the peak fluorescence levels of the reporter strain grown alone in EVCC **(A)** in order to demonstrate changes in intensity when grown in a permissive **(B)** or exclusive **(C)** community.

**Figure 4:**
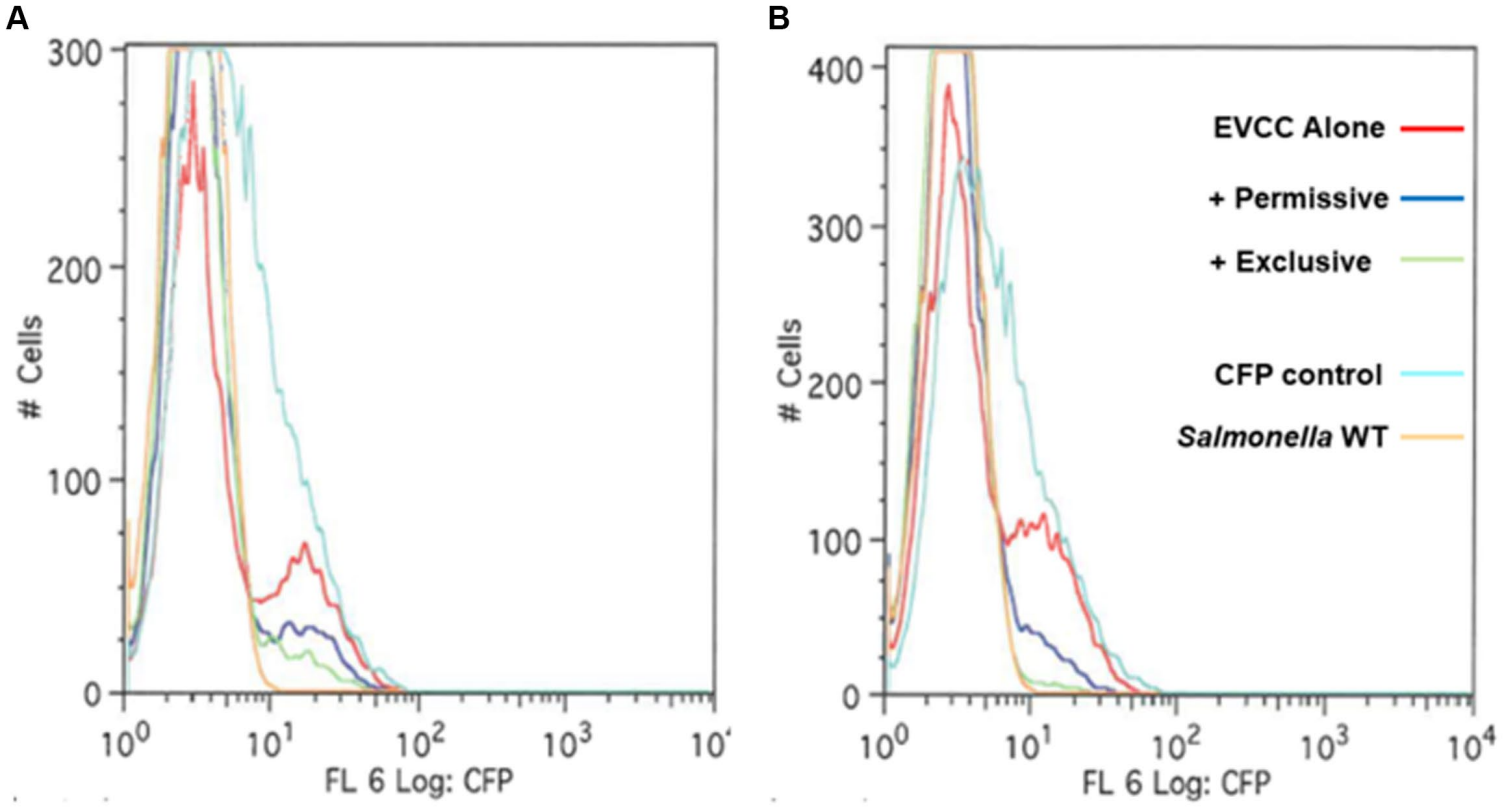
A *hilA* operon*-cfp* promoter fusion using jellyfish cyan fluorescent protein (CFP) as a reporter for *Salmonella* pathogenicity. A “promoter-less” *cfp* was placed between *iag* and the transcriptional terminator for the *hilA* operon using λ Red recombineering (Datsenko and Wanner, 2000). The *Salmonella* reporter strain was grown in EVCC medium alone or EVCC medium inoculated with the permissive or exclusive cecal community at **(A)** 3 h or **(B)** 6-h incubation. FACS analysis was used to measure the *hilA* operon*-cfp* promoter fusion in *S. typhimurium* SL1344 grown in EVCC medium alone (red line) or with a permissive (blue line) or exclusive (green line) community. The *S. typhimurium* SL1344 parental strain (orange line) served as a negative fluorescence control in FACS analysis. The CFP plasmid vector, pMG34, was used as a fluorescence-positive control (cyan line) (Miyashiro and Goulian, 2007). While a fluorescent population of cells were detected for *Salmonella* grown in EVCC alone or in a permissive community, an exclusive community repressed the *Salmonella hilA* locus, which is a global regulator of the pathogenicity island 1 (SPI-1) associated type 3 secretion system (T3SS).

A fluorescence-reporter system was developed to monitor the *Salmonella* response to permissive and exclusive communities *ex vivo*. The growth-dependent promoter for rRNA operon, *rrn* (Bartlett and Gourse, 1994), was fused to the jellyfish fluorescent protein variant YFP and inserted into the P22 prophage integration site within the *Salmonella* chromosome, while CFP was inserted downstream of *iag*, the last gene in SPI-1 *hilA* operon, to monitor expression of the type 3 secretion system (T3SS) cell invasion genes. Both reporter insertions were expected to be neutral, as one was placed within the intragenic site for P22 prophage integration and the other involved insertion behind the last gene of the *hilA* operon *iag*, leaving all genes within this operon intact. The *rrn-yfp* promoter fusion reported was used to monitor *Salmonella* growth. Fluorescence varied depending on growth conditions, with intensive signal observed from cells grown in complex medium (LB vs. minimal medium with glycerol as a carbon/energy source) or at higher growth temperatures in LB broth (37°C vs. 42°C) (Figure 2). *Salmonella* reporter strain’s growth dynamics and growth rate were the same as the wild type under these *ex vivo* conditions with regard to doubling time (Dt) and growth rate (μ) at 30°C vs. 37°C (Supplementary Figure S3; Parish, 1985). When *Salmonella* was grown in EVCC medium, its growth was reduced after 6 h in the exclusive community compared to the permissive community (Figure 3). For *Salmonella* grown in the EVCC alone (control), the intensity of YFP fluorescence diminished over time until there was no detectable fluorescence after 24 h. When grown with either cecal community, *Salmonella* YFP fluorescence decreased to undetectable levels after 9 h. Expression of *Salmonella* T3SS invasion *hilA* operon increased over 6 h in the EVCC control, then decreased to undetectable levels by 24 h. Two peaks were observed in the FACS analysis: a major peak that overlapped with the non-fluorescent *Salmonella* negative control and a minor CFP fluorescent peak, indicating that the majority of cells were not expressing *hilA* (Figure 4). The proportion of *Salmonella* expressing CFP was diminished when grown in the presence of either cecal community after 3 h, but after 6 h of growth in the exclusive community, CFP expression was at its lowest compared to the other two conditions (Figure 4). The data indicate that after 6 h with the exclusive community, *Salmonella* growth was reduced and T3SS invasion expression was repressed. This time point was therefore selected to examine the *Salmonella* transcriptome response to growth in the communities.

**Figure 5:**
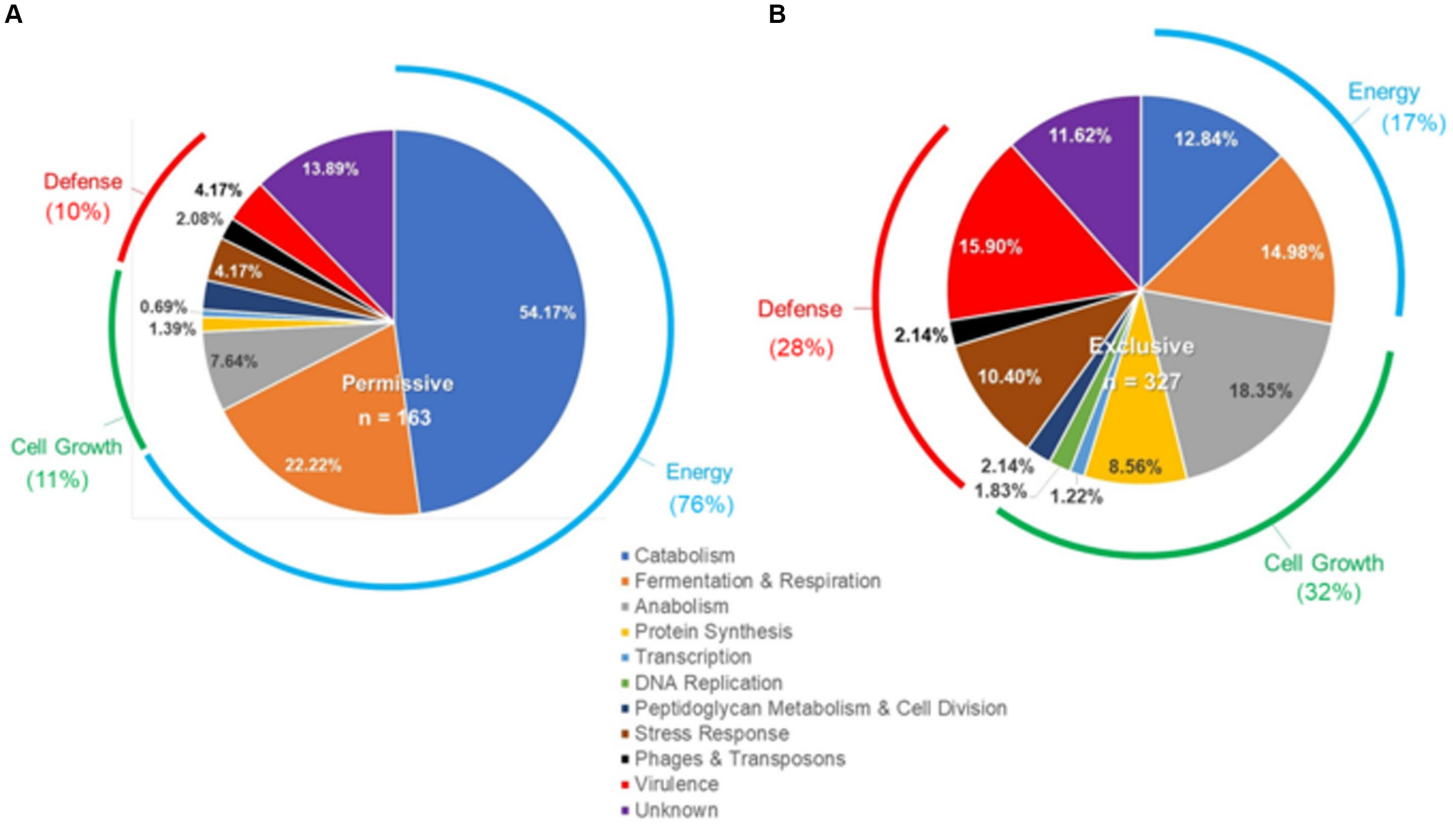
Differences in the *Salmonella* response to cecal permissive **(A)** and exclusive **(B)** communities *ex vivo*. The *Salmonella* SL1344 *rrn-yfp*, *hilA* operon*-cfp* reporter strain was grown in EVCC alone or with a permissive or exclusive community. Total RNA was extracted from *Salmonella* after 6-h growth. Genes reported with elevated expression and significant differences in expression (*p* < 0.0001) for growth in the permissive or exclusive community versus the negative community control were categorized based on function.

**Figure 6:**
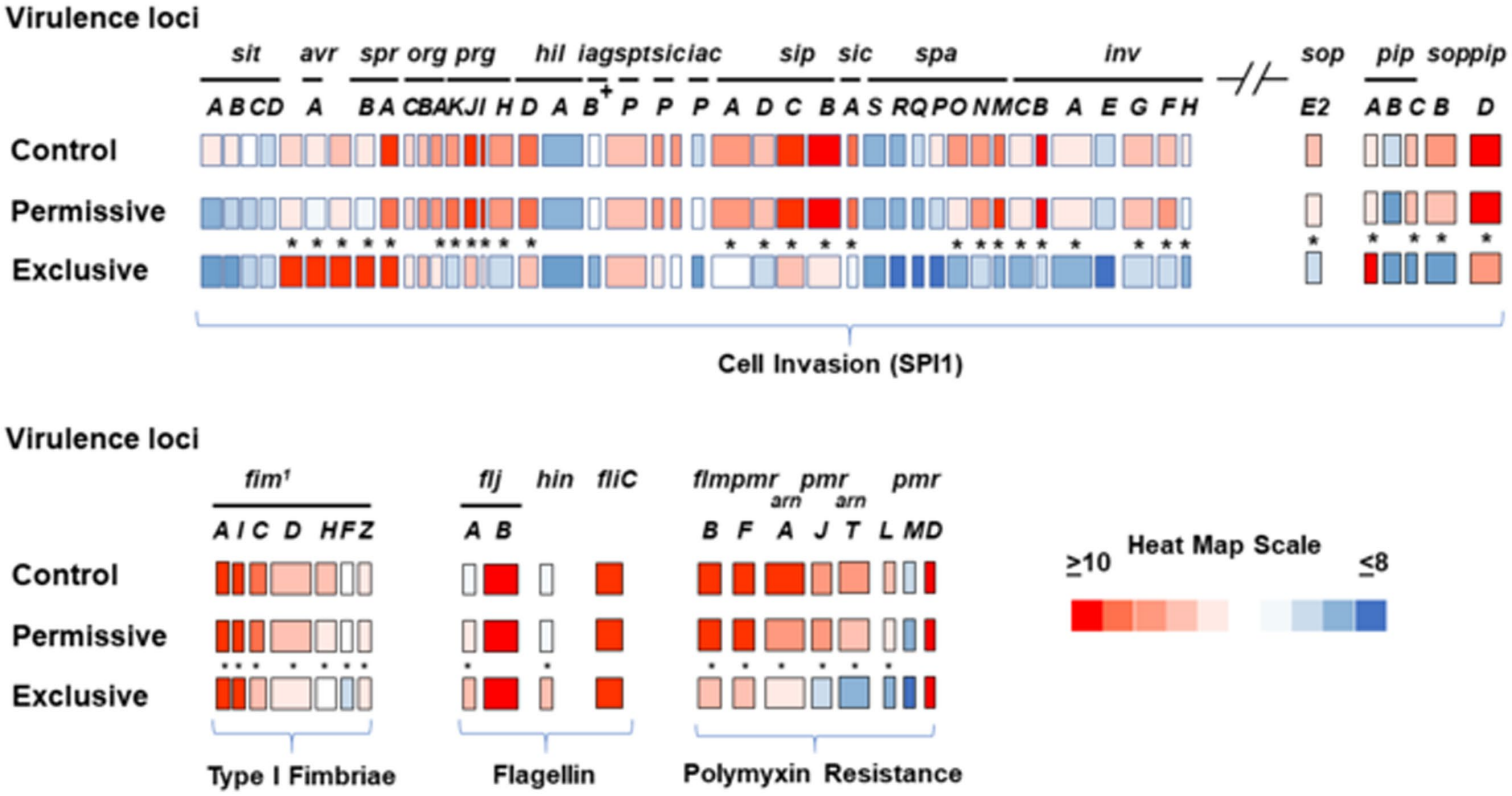
Transcriptional analysis of *Salmonella* virulence expression after growth in permissive or exclusive communities. FACS analysis was used to measure the *hilA* operon*-cfp* promoter fusion expression in *S. typhimurium* SL1344 grown in EVCC or with permissive or exclusive communities. Because Cfp expression was diminished in the *Salmonella* reporter strain after 6 h of growth in the exclusive community, the reporter strain was harvested after 6 h for microarray analysis. Significant transcription differences (*p < 0.0001) were observed in the SPI-1 locus, its ancillary T3SS effectors *sopE2*, *pipA-D*, and *sopB*, the type I fimbriae operon, flagellin, and polymyxin resistance expression. A heat map scale depicts the magnitude of microarray signal.

**Figure 7:**
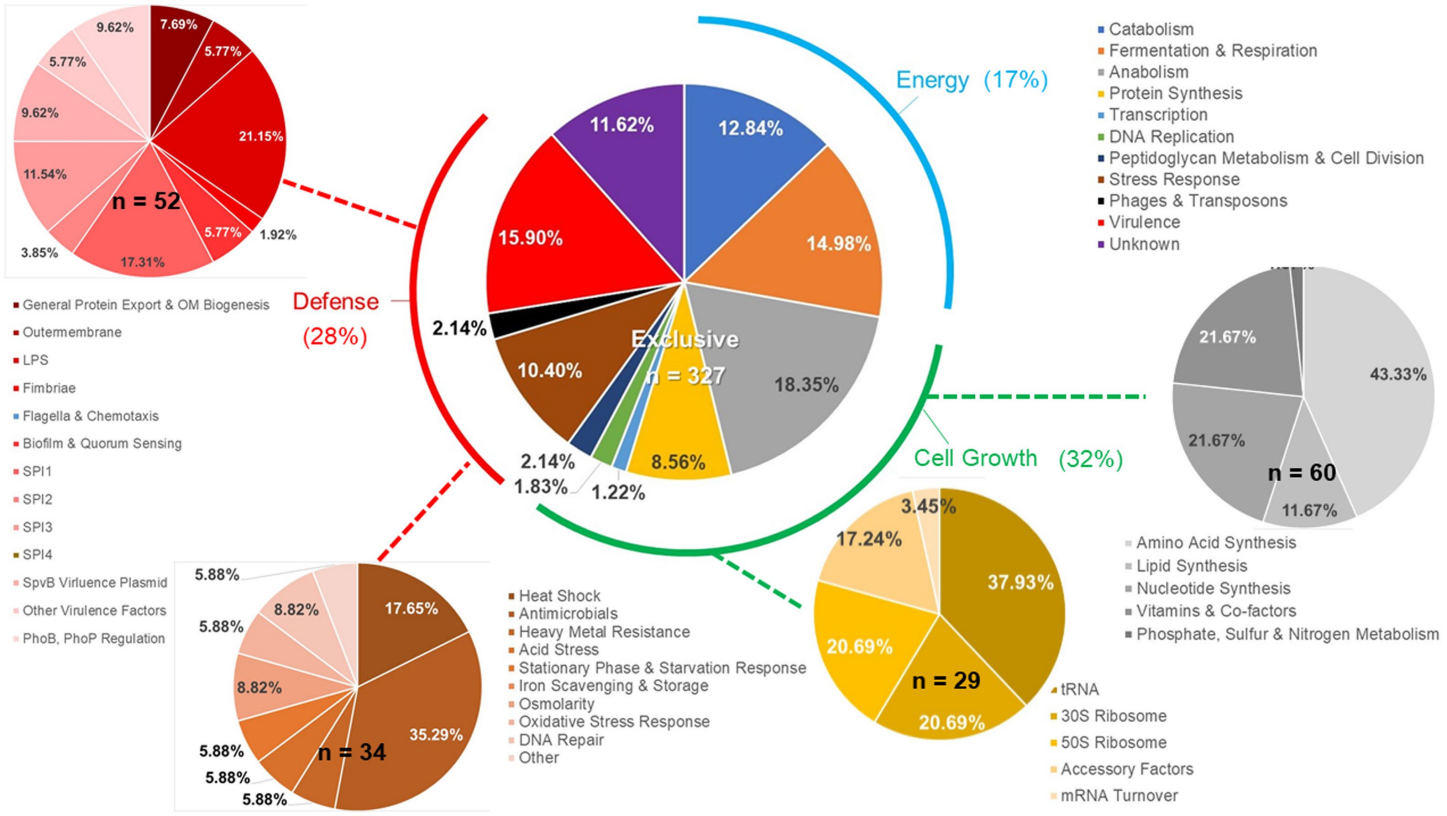
Details of *ex vivo Salmonella* transcriptional response to an exclusive community. *Salmonella* SL1344 *rrn-yfp*, *hilA* operon*-cfp* reporter strain was grown in co-culture in EVCC with an exclusive community or media alone. Total RNA was extracted from *Salmonella* after 6-h growth. Genes reported with significant differences in expression (*p* < 0.0001) versus the medium control were categorized based on function.

Microarray transcriptome analysis of *Salmonella* revealed that the exclusive community had a profound effect on the expression of pathogenic behavior, significantly affecting the expression of 52 virulence genes (15.9%), compared to 7 that were altered when *Salmonella* was grown in the presence of the permissive community (Figure 5). The major differences in virulence gene expression in response to these communities were associated with LPS synthesis and *Salmonella* pathogenicity island 1 (Figures 6, 7). Most of the important virulence genes in SPI-1 as well as its ancillary T3SS effectors were repressed when *Salmonella* was grown with the exclusive community, compared to growth in the permissive community or the EVCC control (*p* < 0.001) (Figure 6). AvrA, an anti-inflammatory/anti-virulence factor (Jones et al., 2008), was elevated in *Salmonella* grown with the exclusive community. The only fimbrial operon expressed under these experimental conditions was the type I fimbrial operon, an important adhesin essential to the cell invasion process (Ernst et al., 1990). While the major fimbrial subunit FimA was expressed under all conditions, there was significantly less expression of export apparatus (*fimC*,*D*) and fimbrial adhesin (*fimF*) (p < 0.001). The polymyxin resistance operon *pmr*, responsible for modifying the *Salmonella* LPS (Gunn, 2008), was also repressed by the exclusive community. These results indicate that attenuation is likely involved in the mechanism of competitive exclusion.

### *Salmonella* catabolic and anabolic response to permissive and exclusive communities *ex vivo*

**Figure 8:**
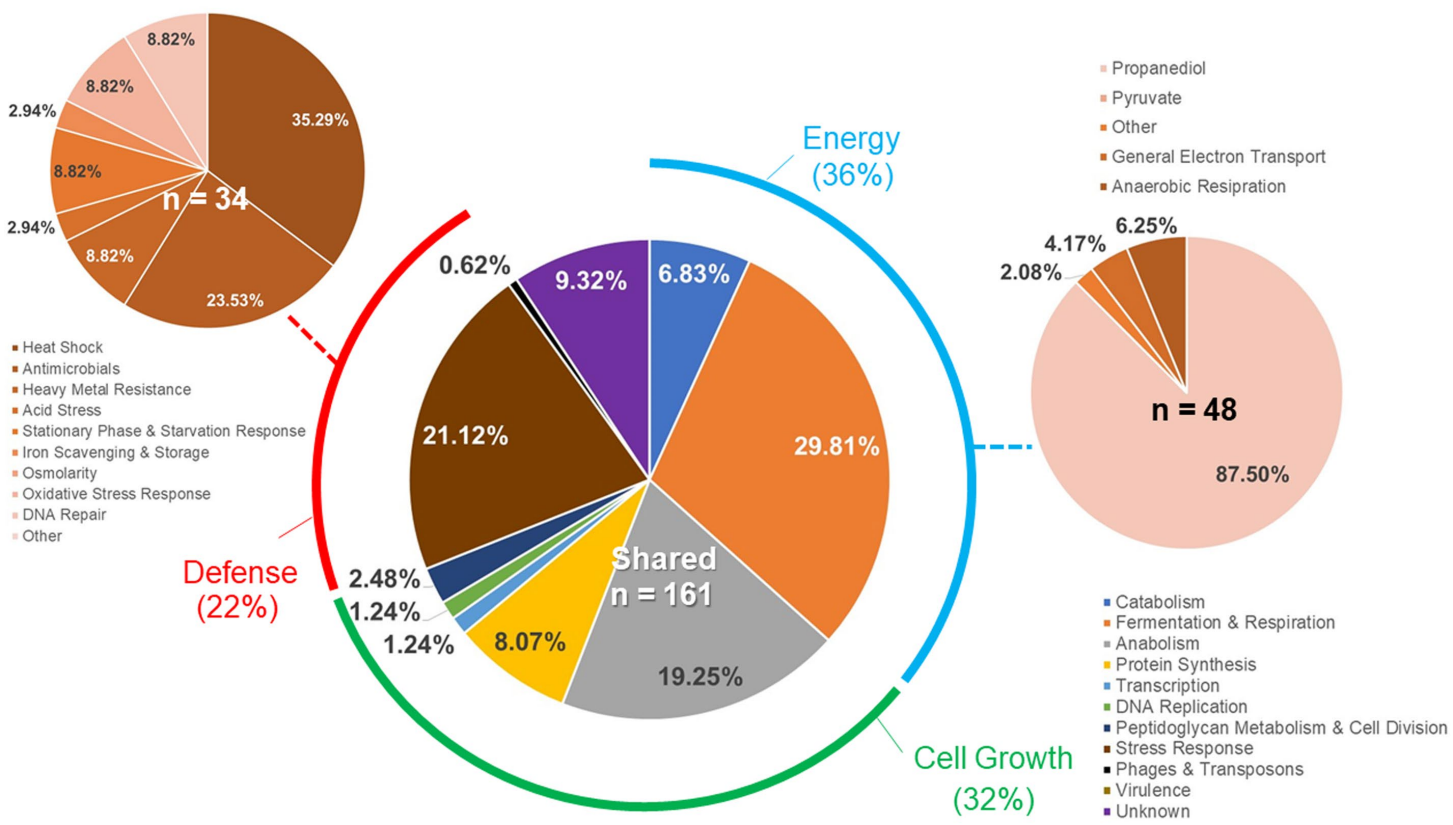
Differences in the *Salmonella* response to cecal permissive or exclusive communities *ex vivo*. The *Salmonella* SL1344 *rrn-yfp*, *hilA* operon*-cfp* reporter strain was grown in EVCC alone or with a permissive or exclusive community. Total RNA was extracted from *Salmonella* after 6-h growth. Genes reported with significant differences in expression (*p* < 0.0001) for both communities versus the negative community control were categorized based on function.

Individual gene transcript levels for salmonellae grown with either permissive or exclusive communities were compared to those of *Salmonella* grown in the EVCC medium alone. Of the 6,069 genes analyzed, 161 were significantly elevated (*p* < 0.0001) in *Salmonella* grown with either community (Figure 8). Thirty-six percent of these transcripts were associated with energy production, and most of these genes (87.5%; *n* = 48) were responsible for propanediol metabolism, the end product of fucose and rhamnose fermentation (Obradors et al., 1988). However, there was low to no expression of transcripts associated with propionate metabolism (*prpB-E*), the end product of propanediol metabolism (Supplemental: *Salmonella* Coculture Data Set). In addition, genes associated with vitamin B12 synthesis (*cbi* operon), a key co-factor involved in both propanediol and ethanolamine metabolism pathways (Jeter, 1990; Brinsmade et al., 2005), were also elevated in *Salmonella* grown with either microbial community, compared to the EVCC control (Table 2). In the presence of the exclusive community, *Salmonella* did not express vitamin B12 transporters (*btuBCD*) compared to EVCC medium alone or with the permissive community. Moreover, ethanolamine utilization transcripts were only expressed in *Salmonella* grown in EVCC alone (Table 2).

**Table 2:** *Salmonella* transcriptome response when co-cultured with cecal communities with high *Salmonella* abundance (permissive), with the competitive exclusion product (exclusive), or in EVCC medium (control).

Function	Genes^1^	Regulation^2^	Control^5^	Permissive^5^	Exclusive^5^
Catabolism					
Arabinose	*ara***C***BAD*; *araE*	*crp*	−	+	−
	*yjcB*	*crp*	−	+*	+
Fructose	*fruAKF*	*crp*	−	+	−
Fucose	*fuc* **R***UKIP*	*crp*	−	+	−
Tagatose	*STM3253-5*		+	−	−
Sialic acid	*nanATEK*	*crp*	+	+	−
d-Glucosaminate	*STM* **4534**		+	+	−
	*STM45354-38*; STM4540.S*		−	+	−
Amino sugars	*STM1130-32*		+	+	−
	*STM1129*		−	+	−
Unknown sugars	*STM3780-85*; *STM1128*; *STM4535-8*		−	+	−
	*STM3774-75*; *STM2289-91*; *STM1613*		+	+	−
	*STM3772*		−	+	−
Gluconate	*hex**R***; *edd*		−	−	+
5-Keto-4-deoxyuronate	*kduDI*		+	**+**	−
Idonate	*idn* **R** *TODK*	*crp*	+	**+**	−
Methyl-galactose	*mglCAB*;**galS**	*crp*	+	+	−
Maltose	*malZ*; *malGF***K**; *malS*	*crp*	−	−	+
Mannitol	*mtl* **R**	*crp*	−	−	+
Glucose–phosphate stress	*sgr* **R**		−	−	+
Carbon starvation	*cstA_1*		+	+	−
	*csiE*	*crp*; *rpoS*	+	−	−
	*yjjY*		−	−	+
C4-Dicarboxylate	*dctA*; *dcuC*	*crp*; *fnr*	+	+	−
	*dcuB*	*crp*; *fnr*	−	−	+
TCA cycle	*acnA*	*fnr*	+	+	−
Cysteine catabolism	*STM0458*		+	+	−
Ethanolamine utilization	*eut* operon^3^	*fur*; *crp*	+	−	−
Respiration					
Electron transport	*rnfABCD*		−	−	+
Cytochrome oxidase	*cyoABCDE*	*fur*; *fnr*	+	+	−
Nitrate reductase	*narGHJI*;**narL**	*fnr*	+	−	−
	*napD*	*fnr*	+	+	−
Formate-dependent nitrate reductase	*nrfCDEG*	*fnr*	+	+	−
Nitrite reductase	*nirBD*	*crp*; *fnr*	−	−	+
Anaerobic sulfide reductase	*asrABC*		−	−	+
Thiosulfate reductase	*phsABC*		+	+	−
Fermentation					
Fermentation end products	*fdnH*; *aldB*	*crp*; *fnr*	+	+	−
Propanediol utilization	*pdu* operon^4^		−	+	+
	*pduX*		−	+	−
Anabolism					
Vitamin B12	*cbiA-E*		−	+	+
Vitamin B12 transport	*btuCD*		+	−	−
	*btuB*		+	+	−
Thiamin biosynthesis	*cof*		+	+	−
Purine synthesis	*purG*; *purM*; *purDH*; *purKE*	*fnr*	−	−	+
	*guaB*	*crp*; *rpoS*	−	−	+
Allantoin	*gcl*; *gip*; *glxR*; *allPR*; *ybbY*; *glxK*; *allCD*	*rpoS*	+	−	−
Pyrimidine metabolism	*STM2186-7*		+	+	−
Nucleotide metabolism	*ndk*; *cdpB*; *nupG*	*crp*	+	+	−
	*STM3473*; *ibrA*; *yjcD*	*crp*	−	−	+
Peptide transport	*STM3592*; *STM2759*		+	+	−
Methionine	*metF*; *metN*		−	−	+
Serine	*sdaB*	*crp*	+	+	−
Threonine	*tdcA*	*crp*; *fnr*	+	+	−
Arginine	*carAB*		+*	−	+
Asparagine	*asnCA*		−	−	+
	*ansB*	*crp*	+	+	−
Cysteine	*cysK*; *eamA*		+	+	−
Glutamate	*gluD*		+	+	−
Alanine	*dadA*	*crp*; *rpoS*	+	+	−
	*dadX*; *STM1633*	*crp*	+	−	−
Stringent response	*ytfK*		+	+	_
Fatty acid metabolism	*aidB*; *ugpB*; *ucpA*	*crp*	+	+	−
Ferrichrome transport	*fhuA*; *STM0191*	*fur*	+	−	−
Iron	*bfd*; *ryhB*	*fur*; *rpoS*	+	−	−
Protein synthesis	*queA*; *trpS2*; *yadB*		−	−	+
	*STM4446*		−	+	−
Replication	*tus*		+	−	−
DNA repair	*STM1514*		+	−	−
Regulation					
Transcription factors	*phoH*; *rsd*; *arcZ*	*rpoS*	+	+	−
	*STM1001*		+	−	−
	*adiY*		−	+	+
	*STM3834*		−	+	−
	*crp/cyaA*; *fnr*; *fur*; *rpoS*		+	+	+
Virulence and stress response					
Quorum sensing (*lsr*)	*STM4071-80*	*crp*	+	+	−
SPI-1	*invFGBJ*; *spaO*; *sicA*; *sipD*; *prgHK*		+	+	−
	*sitABC*	*fur*	+	−	−
	*pipC*; *sopB*; *orfX*		+	+	−
	*pipA*		−	−	+
SPI3	*mgtBC*		−	−	+
Polymyxin resistance	*pmrJ*; *arnT*; *pmrL*; *ybjG*		+	+	−
Other virulence factors	*virK_2*		−	−	+
	*rck*		−	+	+
Fimbriae	*stcA*		+	+	−
Motility and chemotaxis	*trg*; *flgJ*; *STM3156*; *STM3604*		+	+	−
Outer membrane	*pgtE*		−	−	+
	*yhcN*; *STM3361-2*		−	+	+
	*STM0080*		+	−	−
	*yhfL*		+	+	−
Cell wall	*cidAB*		−	−	+
Oxidative stress	*srgA*		−	−	+
	*yciGFE*		+	−	−
Osmotic stress	*yehY*		+	+	−
Antimicrobials	*emrD*		−	−	+
	*yabI*		−	+	−
	*ydhE*		+	−	
CRISPR	*STM2938-93*; *STM2937-43*		+	+	−
Phage	*STM2740*				
Unknown					
	*yeiH*		+*	−	+
	*STM4441-2*	*crp*	−	+	−
	*STM0514*; *yhhX*		+	−	−
	*ybhQ*; *ygjR*; *yidE*; *yiiL*; *yjcO*; *ylbA*; *STM0660*; *STM1810*; *STM1933*; *STM2950*; *STM3343*; *STM4503*	*crp*	+	+	−
	*ybdH*; *yfhL*		−	−	+

^1^Bold-transcription factor. ^2^crp-regulated genes (regulon of Crp in *Salmonella* Typhimurium LT2; https://regprecise.lbl.gov/regulon.jsp?regulon_id=10138; accessed 08/02/2023); fur-regulated genes [(Troxell et al., 2011) or Regulon of Fur in *Salmonella* Typhimurium LT2 https://regprecise.lbl.gov/regulon.jsp?regulon_id=10114; accessed 08/02/2023]; fnr-regulated genes (Regulon of Fnr in *Salmonella* Typhimurium LT2; https://regprecise.lbl.gov/regulon.jsp?regulon_id=41958) or rpoS-regulated (Wong et al., 2017). ^3^eut**R**AHGJ; eutD; eutS. ^4^pduGH; pduKLMOPQSTUV; *Weak expression. +: “on”; −: “off”; crp: catabolite repression; fnr: anaerobic metabolism; and fur: iron-regulated genes; rpoS: stationary phase response. ^5^“+”: upregulated; “−” downregulated.

There were significant differences in the *Salmonella* global transcriptome response to the microbial communities. Over 75% of 163 elevated gene transcripts from *Salmonella* grown with the permissive community, compared to the control, were dedicated to energy metabolism, whereas this was only the case for 17% of 327 elevated gene transcripts for *Salmonella* grown with the exclusive community (Figure 5). *Salmonella* grown with the permissive community directed fewer resources toward anabolism, as a percentage of upregulated genes (8%) than those grown with the exclusive community, where 18% of the upregulated genes were devoted to anabolism (Figure 5). *Salmonella* metabolic genes associated with the catabolism of arabinose, fructose, fucose, d-glucosaminate, and other amino-sugars were exclusively upregulated in *Salmonella* grown with the permissive community, while maltose was the only sugar utilization pathway expressed by *Salmonella* in co-culture with the exclusive community (Table 2). Several genes annotated as sugar transporters and associated enzymes, whose substrates have yet to be identified, were also expressed in *Salmonella* grown with the permissive community. In addition, *Salmonella* grown in EVCC medium alone or with the permissive community also upregulated genes responsible for sialic acid metabolism (*nanATEK*) and transport of methyl-galactose (*mglCAB*, *galS*). Catabolite repression may be central to regulating *Salmonella* gene expression in either microbial community, as several of these and other catabolic genes tied to energy generation (i.e., respiration) and a few genes with a role in the anabolic pathways responsible for nucleotide and amino acid metabolism possessed the signature nucleotide sequence recognized by the catabolite repressor protein Crp (Gunasekera et al., 1992). Transcripts for *crp* and the adenylate cyclase *cyaA* were produced in *Salmonella* under all growth conditions. *Ex vivo*, *Salmonella* produced enzymes under all conditions for metabolizing glucose (*ptsG*, *glk*, *pfkAB*, *fdaB*, *tpiA*, *pgk*, *gpmA*, *eno*, *pykF*), galactose (*galMKTE*), mannose (*manA*, *manXYZ*), glucuronate/galacturonate (*kdgT*, *uxaA*, *uxaC*), N-acetyl-galactosamine (*gatY*), and N-acetyl-glucosamine (*nag* operon) (Supplemental: *Salmonella* Coculture Data Set). There was greater expression for several of these genes (*ptsG*, *glk*, *fdaB*, *manXYZ*) in *Salmonella* grown with the permissive cecal community versus the exclusive community.

As propanediol is an end product of fucose and rhamnose fermentation, the focus of the *Salmonella* transcriptome analysis was shifted toward metabolism of microbial metabolites, including volatile fatty acids. Several genes tied to acetate (*ackA*, *pta*, *poxB*), d-lactate (*dld*), and hydrogen (hydrogenase-1, hydrogenase-2, and hydrogenase-3) metabolism were expressed under all conditions (Supplemental: *Salmonella* Coculture Data Set). However, neither acetate nor lactate was likely to be serving as a resource provided by its microbial community members, as the acetate permeases ActP and YaaH and lactate permease LldP were not elevated in *Salmonella* under any growth conditions. Differential expression was observed for formate (*fdnH*) and aldehyde (*aldB*) dehydrogenases in *Salmonella* grown in the presence of the microbial communities. However, the highest transcript levels of these genes were detected in *Salmonella* grown in the absence of either community.

Respiration may be central to *Salmonella* growth in that NAD appeared to be regenerated via electron transport, and NADH dehydrogenase genes (*nuo* operon) were strongly expressed under all growth conditions. In addition, cytochrome d oxidase genes (*cydAB*) were expressed under all growth conditions, whereas there was a significant decrease in cytochrome o oxidase *cyo* operon transcripts in *Salmonella* grown with the exclusive community (Table 2). While aerobic respiration appeared to be involved in *Salmonella* metabolism, several enzymes involved in anaerobic respiration were also differentially transcribed by *Salmonella* grown in EVCC alone (nitrate reductase: *narGHIJ*, *narL*), with the permissive community (nitrate reductases: *napD*; *nrfCDEG*; thiosulfate reductase: *phsABC*) or the exclusive community (nitrite reductase: *nirBD*; anaerobic sulfide reductase: *asrABC*).

Finally, there were substantial differences in the upregulation of anabolic enzyme transcripts between *Salmonella* grown with either community compared to EVCC medium alone (7% vs. 20%, respectively; Figure 5). In *Salmonella* grown in the presence of the exclusive community, a total of 65% of these anabolic enzymes were dedicated to amino acid and nucleotide synthesis. *Salmonella* grown with the exclusive community-expressed enzymes needed to synthesize nucleotides (*STM3473*; *ibrA*; *yicD*), especially purines (*purG*; *purM*; *purDH*; *purKE*; *guaB*) (Table 2). The peptide transporters STM3592 and STM2759 were expressed by *Salmonella* with the permissive community, as well as several enzymes associated with amino acid metabolism for serine (*sdaB*), threonine (*tdcA*), asparagine (asparaginase *ansB*), cysteine (*cysK*, *eamA*), glutamate (*gluD*), and d-alanine (*dadA*). There were significantly fewer transcripts found in the expression of enzymes involved in catabolism of serine/threonine (*sdaA*, *sdaBC*; *tdc* operon) in *Salmonella* grown with the exclusive community (Supplemental: *Salmonella* Coculture Data Set). These catabolic enzymes are subject to catabolite repression by Crp (Table 2). Methionine (*metF*, *metN*), arginine (*carAB*), and asparagine (*asnCA*) anabolism enzyme transcripts were upregulated in *Salmonella* grown with the exclusive community.

### Cecal communities in chickens with high *Salmonella* abundance have a volatile fatty acid metabolic network

**Figure 9:**
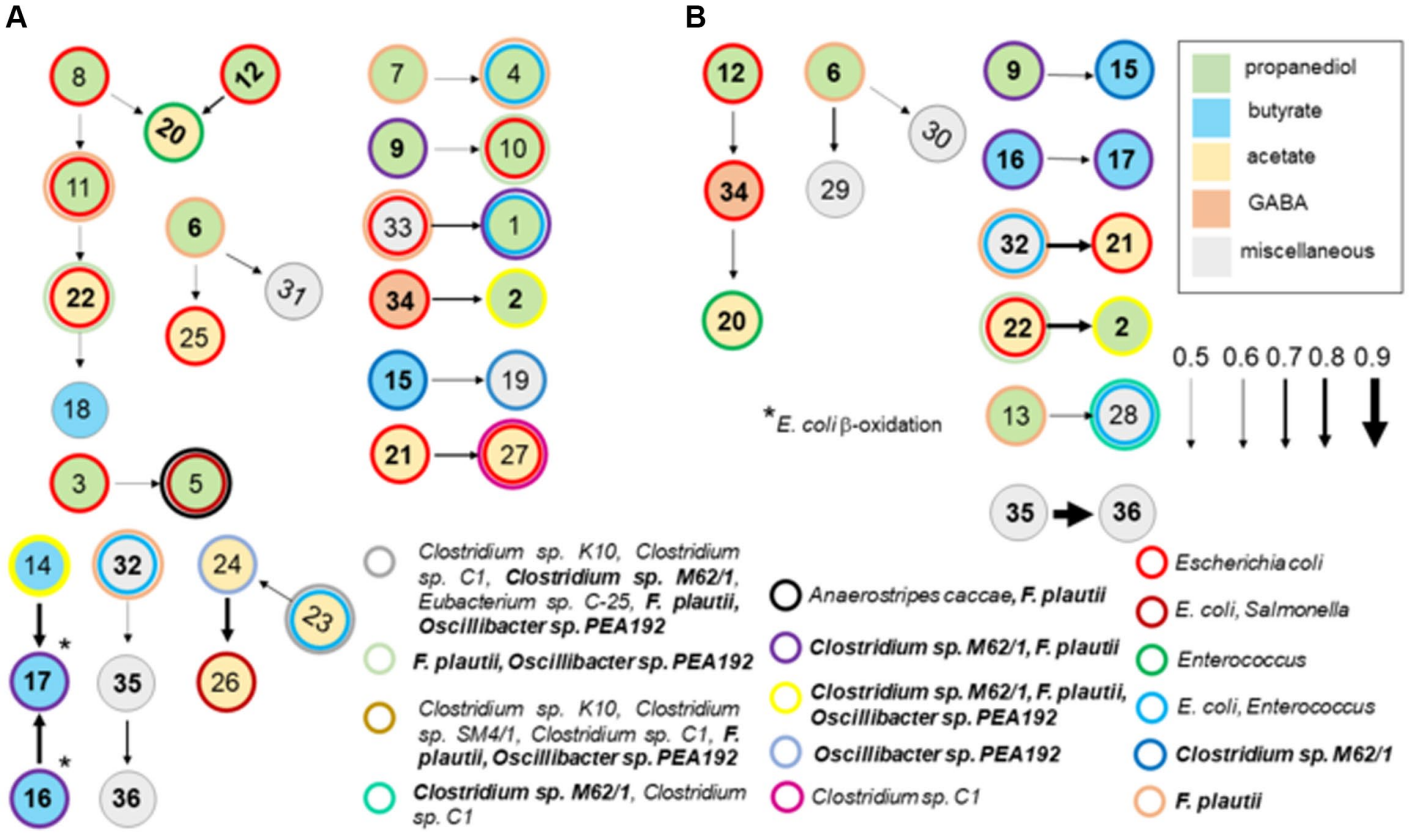
A Bayesian network analysis of cecal transcriptome, focused on fermentation, from chickens with high (>5.85 Log_10_ *Salmonella* genomes/g cecal contents) **(A)** or low **(B)** *Salmonella* abundance. The network depicted was identified using the score-based learning algorithm Hill-Climbing. The data were obtained from KO metabolism data sets in MG-RAST. Connections were identified among the 45 enzyme transcripts by network analysis, of which 36 were identified as single or multiple connections, producing 20 and 10 connections from the transcriptomes of cecal communities with high or low *Salmonella* abundance, respectively. Arrows indicate the direction and strength of the connection. Enzymes are color-coded based on their associated propanediol (green), butyrate (blue), or acetate (yellow) fermentation pathways. Enzymes identified in these networks were: methyl-malonyl-CoA decarboxylase (1); methylglyoxal synthase (2); lactaldehyde dehydrogenase (3); hydroxyacylglutathione hydrolase (4); glycerol dehydrogenase (5); glycerol dehydratase (6); propanediol dehydratase (7); propionate kinase (8); propionyl-CoA carboxylase (9); propionate-CoA transferase (10); methyl-malonyl-CoA mutase (11); propionyl-CoA synthase (12); propionaldehyde dehydrogenase (13); butyryl-CoA dehydrogenase (14); glutaconate-CoA transferase (15); hydroxybutyryl-CoA dehydratase (16); hydroxybutyryl-CoA dehydrogenase (17); thiolase (18); ferredoxin hydrogenase (19); pyruvate oxidase (20); acetyl-CoA synthase (21); acetyl-CoA hydrolase (22); acetate kinase (23); pyruvate ferredoxin oxidoreductase (24); acetyl-CoA synthetase (25); aldehyde dehydrogenase (26); phosphotransacetylase (27); lactate dehydrogenase (28); phosphoketolase (29); glutamate synthase (30); methylaspartate ammonia-lyase (31); ethanolamine ammonia-lyase (32); ethanolamine utilization protein (33); 4-aminobutyrate aminotransferase (34); cobalamin (vitamin B12) (35); and cobalamin biosynthesis (36). The following enzymes were not identified in any network analysis: lactaldehyde reductase, butyrate kinase, pyruvate dehydrogenase, citramalate synthase, serine dehydratase, alanine dehydrogenase, formate hydrogenlyase, formate C-acetyltransferase, and glutamate mutase. Numbers in bold are enzymes shared between cecal communities with high and low *Salmonella* abundance. Circles with thick-colored borders are for enzymatic transcripts with 98–100% nucleotide identity and 99–100% coverage by BLAST scores for intestinal species including *Escherichia coli*, *Enterococcus faecium*, and *Flavonifractor plautii*.

A Bayesian network analysis was used to analyze the cecal transcriptome pulled from MG-RAST-generated metabolism data sets. Guided by the findings of the *ex vivo* transcriptome, analysis of the cecal transcriptome was focused on enzyme transcripts involved in fermentation. A Hill-Climbing network identified 36 enzymes with 20 total connections (total enzymes, *n* = 45), of which there were seven single connections and five multiple connections with three or more enzymes (Figure 9) for the cecal communities with high *Salmonella* abundance (>5.85 Log_10_ *Salmonella* genomes/g cecal contents). Twelve enzymes associated with propanediol fermentation were identified in this network analysis in cecal communities with high *Salmonella* abundance. Propionate kinase PduW was a major node connected to enzymes involved in propanediol and acetate metabolism. The network connections for the high *Salmonella* abundance group mirrored their enzymatic positions within their respective metabolic pathways for some enzyme transcripts. Ten enzyme connections involving 18 enzymes were identified from the low *Salmonella* abundance cecal transcriptome. Here, propanediol-associated enzymes: methylglyoxal synthase, glycerol dehydratase, propionyl-CoA carboxylase, propionyl-CoA synthase, and propionaldehyde dehydrogenase were identified. Fourteen enzymes were present in both groups and included: methylglyoxal synthase; glycerol dehydratase; propionyl-CoA carboxylase; propionyl-CoA transferase; glutaconate-CoA transferase; hydroxybutyryl-CoA dehydratase; hydroxybutyryl-CoA dehydrogenase; pyruvate oxidase; acetyl-CoA synthase; acetyl-CoA hydrolase; ethanolamine ammonia-lyase; 4-aminobutyrate aminotransferase; cobalamin (vitamin B12); and cobalamin biosynthesis; although some of these were in different enzyme connections between these two groups.

As these enzymatic pathways are associated with carbohydrate or amino acid fermentation, network analysis was expanded to include metabolic enzyme transcripts (164 total enzymes) with polysaccharide degradation and fucose/rhamnose catabolism responsible for producing the fermentation end product propanediol. Forty-four were identified in single or multiple connections for the two groups, producing 26 and 17 connections from the cecal transcriptomes for the high and low *Salmonella* abundance groups, respectively (Supplementary Figure S4). Propanediol and acetate metabolism remain dominant metabolic pathways in this new network analysis, especially for the high *Salmonella* abundance group, pulling in additional enzymes mostly associated with glucose metabolism and the TCA cycle. In fact, the same 30 fermentation enzymes from the first network analysis were identified in the expanded network analysis, except there were fewer single-enzyme connections for both *Salmonella* abundance groups. As expected, the expanded network analysis identified similar connections in the high *Salmonella* abundance group compared to the original 45-gene analysis (Supplementary Figure S3; Figure 9). Three of 10 enzyme transcripts involved with fermentation (*n* = 25), propionyl-CoA carboxylase, butyryl-CoA dehydrogenase, and pyruvate dehydrogenase, also had a significantly positive correlation with low *Salmonella* abundance (<6.00 Log_10_ *Salmonella* genomes/g cecal contents) (Table 3), while propionyl-CoA synthase and pyruvate oxidase had a significantly negative correlation with *Salmonella* abundance at *Salmonella* levels <6.00 Log_10_ *Salmonella* genomes/g of cecal contents. Other propionate enzyme transcripts (propionaldehyde dehydrogenase and propionate kinase) exhibited a negative correlation (*p* < 0.05) related to the age of the bird (not shown).

**Table 3:** Cecal microbial community enzyme transcripts correlate with *Salmonella* abundance.

Enzyme transcripts^1^	*Salmonella* abundance (Log_10_ genomes/g^2^)	Correlation coefficients^3^			
		Pearson		Spearman	
		*r* =	*p* =	*ρ* =	*p* =
Carbohydrate utilization					
α-Galactosidase	7.00–9.22	0.7965	0.9939	**0.7186**	**0.0340**
	6.79–9.22	0.5445	0.9519	−0.3830	**0.0259**
Galactose-1-phosphate uridyltransferase	2.11–6.89	−**0.7275**	**0.0159**	−0.5952	0.0977
	2.11–5.85	−**0.7628**	**0.0282**	−0.7714	0.0535
Arabinogalactan endo-1,4-β-galactosidase	2.11–5.85	0.7275	0.9609	**0.8286**	**0.0297**
Ribose/xylose/arabinose/galactoside ABC-type transport system	0.00–5.85	0.5329	0.9193	**0.7489**	**0.0243**
β-Fructofuranosidase	0.00–5.85	−0.5082	0.0932	−**0.7553**	**0.0225**
	7.00–9.22	0.6920	0.9766	0.6826	**0.0484**
Phosphofructokinase	7.00–9.22	−**0.7106**	**0.0192**	−**0.7904**	**0.0142**
α-l-Fucosidase	2.11–5.85	−**0.7284**	**0.0388**	−0.5429	0.2219
l-Fuculokinase	7.00–9.22	0.8723	0.9988	**0.8503**	**0.0052**
l-Fucose isomerase and related proteins	7.00–9.22	0.7387	0.9861	**0.8144**	**0.0099**
	2.11–6.89	0.6788	0.9733	**0.8095**	**0.0107**
l-Fucose isomerase	7.00–9.22	0.7387	0.9861	**0.8144**	**0.0099**
	2.11–6.89	0.7387	0.9861	**0.8144**	**0.0099**
Rhamnulokinase	7.00–9.22	0.7123	0.9811	**0.7425**	**0.0262**
N-acetylneuraminate lyase	0.00–5.85	0.6925	0.9767	**0.7085**	**0.0377**
	7.00–9.22	−0.6692	**0.0292**	−0.6707	0.0539
ABC-type maltose transport system	0.00–5.85	0.0596	0.5563	**0.8193**	**0.0092**
Maltose-binding periplasmic proteins	2.11–5.85	−**0.8447**	**0.0098**	−**0.8986**	**0.0102**
β-hexosaminidase	0.00–5.85	0.4909	0.8976	0.6991	**0.0414**
Dehydro-3-deoxyphosphogluconate aldolase	0.00–5.85	0.4909	0.9209	**0.9429**	**0.0032**
Aldose 1-epimerase	2.11–6.89	−0.5501	0.0727	−**0.7381**	**0.0275**
	2.11–5.85	−0.6470	0.0703	−**0.8286**	**0.0297**
α-Glucuronidase	2.11–5.85	−**0.7086**	**0.0457**	−0.3714	0.4216
β-Glucuronidase	0.00–5.85	−0.388	0.4633	**0.7009**	**0.0407**
α-Glucosidase	2.11–6.89	−**0.7193**	**0.0174**	−0.5238	0.1549
Glucose-6-phosphate 1-dehydrogenase	0.00–5.85	−0.0222	0.4790	−**0.7664**	**0.0197**
Phosphoglycerate kinase	7.00–9.22	−0.5926	0.0546	−**0.7785**	**0.0168**
β-Xylosidase	2.11.5.85	−**0.8864**	**0.0043**	−**0.8857**	**0.0130**
Fermentation					
Glycerol dehydratase	0.00–5.85	−0.1834	0.3299	−**0.8131**	**0.0101**
Propionyl-CoA carboxylase	2.11–5.85	0.7350	0.9633	**0.9429**	**0.0032**
Propionyl-CoA synthase	2.11–5.85	−**0.8424**	**0.0102**	−0.6547	0.1249
Butyryl-CoA dehydrogenase	0.00–5.85	0.0013	0.5012	**0.9364**	**0.0004**
Glutaconate-CoA transferase	0.00–5.85	0.4489	0.8733	0.6837	**0.0479**
	7.00–9.82	−0.6112	**0.0476**	−0.4671	0.2117
Pyruvate dehydrogenase	0.00–6.89	0.4684	0.9177	**0.8079**	**0.0034**
	2.11–6.89	0.4946	0.8996	**0.7143**	**0.0356**
	2.11–5.85	0.8899	0.9960	**0.8857**	**0.0130**
Pyruvate oxidase	2.11–6.89	−**0.7811**	**0.0077**	−0.5774	0.1106
	2.11–5.85	−**0.8424**	**0.0102**	−0.6547	0.1249
Acetyl-CoA hydrolase	0.00–5.85	0.4381	0.8666	**0.7131**	**0.0360**
	2.11–8.51	−0.4781	**0.0401**	−0.5919	**0.0244**
Na + −transporting methyl-malonyl-CoA/oxaloacetate decarboxylase	0.00–6.89	−**0.7383**	**0.0057**	−0.6687	**0.0272**
	0.00–5.85	−**0.7084**	**0.0197**	−0.4669	0.2119
N-methylhydantoinase A/acetone carboxylase	0.00–6.89	−**0.7289**	**0.0065**	−**0.7877**	**0.0050**
TCA					
Citrate lyase	0.00–5.85	0.1961	0.6813	−**0.7175**	**0.0344**
Malic enzyme	7.00–9.22	0.8016	0.9944	**0.8982**	**0.0017**
Pyruvate/oxaloacetate carboxyltransferase	2.11–5.85	0.7265	0.9605	**0.8286**	**0.0297**
Respiration					
Ubiquinone oxidoreductase	2.11–6.89	−0.3813	0.1709	−**0.7143**	**0.0356**
Cytochrome bd-type quinol oxidase	0.00–6.89	0.6345	0.9787	**0.8024**	**0.0038**
	2.11–6.89	0.6098	0.9519	**0.7619**	**0.0208**
	2.11–5.85	0.7100	0.9548	**0.8286**	**0.0297**
Cytochrome o ubiquinol oxidase	2.11–5.85	−**0.7651**	**0.0275**	−**0.8197**	**0.0329**
Nitrate reductase	2.11–6.89	0.5273	0.9165	**0.8024**	**0.0120**
Nickel-dependent hydrogenase	7.00–9.22	0.6132	0.9531	**0.7545**	**0.0228**
	2.11–6.89	0.7117	0.9810	**0.9048**	**0.0014**
	2.11–5.85	0.7408	0.9652	**0.8286**	**0.0297**
Amino acid/nitrogen metabolism					
Glutamate dehydrogenase	0.00–5.85	0.2271	0.7082	−**0.7175**	**0.0344**
ABC-type histidine transport system	2.11–6.89	−0.6175	**0.0453**	−**0.8810**	**0.0026**
ABC-type polar amino acid transport system	0.00–5.85	−0.5227	0.0858	**0.8217**	**0.0088**
Dipeptidase	2.11–6.89	−0.5522	0.0717	−**0.7319**	**0.0295**
Periplasmic component/domain	0.00–5.85	0.5861	0.9428	**0.7569**	**0.0221**
Histidinol-phosphate/aromatic aminotransferase and cobyric acid decarboxylase	0.00–6.89	−0.5063	0.0639	−**0.7416**	**0.0106**
Isopropylmalate/homocitrate/citramalate synthases	0.00–5.85	−0.5417	0.0765	**0.7413**	**0.0266**
Transglutaminase-like enzyme	7.00–9.22	0.4183	0.8540	**0.7349**	**0.0285**
Tryptophanase	7.00–9.22	0.6833	0.9745	**0.7401**	**0.0269**
Zinc metalloprotease (elastase)	7.00–9.22	0.6567	0.9673	**0.8507**	**0.0052**
	2.11–6.89	−**0.7397**	**0.0137**	−0.3546	0.3544
5-Enolpyruvylshikimate-3-phosphate synthase	0.00–5.85	−0.0925	0.4128	−**0.9157**	**0.0009**
Acetylglutamate kinase	0.00–5.85	0.2930	0.7628	**0.8109**	**0.0105**
Acetylornithine deacetylase/succinyl-diaminopimelate desuccinylase	0.00–5.85	−0.1849	0.3286	−**0.7522**	**0.0234**
	2.11–6.89	0.8070	0.9949	**0.8571**	**0.0046**
Allophanate hydrolase	2.11–5.85	−**0.7979**	**0.0191**	−0.6000	0.1688
	0.00–5.85	−**0.8984**	**0.0005**	0.5747	0.1125
Amino acid permeases	0.00–6.89	0.5764	0.9630	**0.8511**	**0.0013**
	2.11–6.89	0.5355	0.9205	**0.7143**	**0.0356**
Ammonia permease	7.00–9.22	0.7553	0.9888	**0.8144**	**0.0099**
	2.11–6.89	−0.5740	0.0622	−**0.7143**	**0.0356**
Anthranilate phosphoribosyltransferase	7.00–9.22	0.9302	0.9999	**0.8862**	**0.0023**
Anthranilate/para-aminobenzoate synthase	7.00–9.22	0.6953	0.9774	**0.7904**	**0.0142**
Aspartate/tyrosine/aromatic aminotransferase	2.11–6.89	−0.6139	**0.0466**	−**0.7381**	**0.0275**
Aspartate ammonia-lyase	0.00–6.89	−0.6037	**0.0289**	−**0.8207**	**0.0026**
Aspartyl aminopeptidase	2.11–6.89	−**0.7314**	**0.0152**	−0.6190	0.0821
Aspartate-semialdehyde dehydrogenase	0.00–5.85	−0.1194	0.3880	−**0.7396**	**0.0271**
Chorismate synthase	0.00–5.85	0.5157	0.9107	**0.8456**	**0.0057**
	7.00–9.22	−**0.7571**	**0.0109**	−**0.7545**	**0.0228**
Di- and tripeptidases	7.00–9.22	0.8528	0.9980	**0.8503**	**0.0052**
γ-Aminobutyrate permease and related permeases	2.11–6.89	0.7297	0.9845	**0.7857**	**0.0152**
	0.00–6.89	0.7358	0.9941	**0.8875**	**0.0004**
Glutamate 5-kinase	0.00–5.85	0.0364	0.5344	**0.7228**	**0.0325**
Glutaminase	0.00–5.85	−**0.7147**	**0.0184**	0.1199	0.7601
Glutamine amidotransferase	0.00–6.89	−0.6801	**0.0127**	−**0.7356**	**0.0116**
Glutamine synthetase	7.00–9.22	0.7693	0.9908	**0.8503**	**0.0052**
Glycine cleavage system	2.11–8.51	−**0.8150**	**0.0153**	−**0.8286**	**0.0297**
Histidine ammonia-lyase	7.00–9.22	0.5501	0.9274	**0.7186**	**0.0340**
Histidinol dehydrogenase	0.00–6.89	−**0.7217**	**0.0073**	−**0.8024**	**0.0038**
Imidazolonepropionase	0.00–5.85	0.3145	0.7798	−**0.8977**	**0.0017**
Arginine lysine ornithine decarboxylases	2.11–5.85	−**0.8961**	**0.0034**	–	–
l-Serine deaminase	0.00–6.89	−**0.7128**	**0.0083**	−**0.7052**	**0.0175**
Threonine efflux protein	2.11–5.85	−**0.8012**	**0.0183**	−0.4286	0.3486
Na+/alanine symporter	2.11–6.89	−0.4081	0.1527	−**0.7143**	**0.0356**
O-Acetylhomoserine sulfhydrylase	0.00–6.89	−0.6020	**0.0294**	−**0.7173**	**0.0149**
Oligoendopeptidase F	0.00–6.89	−0.6813	**0.0125**	−**0.7052**	**0.0175**
Peptidylarginine deiminase	2.11–6.89	−0.4507	0.1256	−**0.8095**	**0.0107**
Phosphoribosylanthranilate isomerase	2.11–5.85	−**0.7429**	**0.0341**	−**0.8286**	**0.0297**
Uncharacterized protein involved in cysteine biosynthesis	2.11–6.89	−**0.7811**	**0.0077**	−0.5774	0.1106
	2.11–5.85	−**0.8424**	**0.0102**	−0.6547	0.1249
Urease	2.11–6.89	−0.6991	**0.0217**	−0.3095	0.4222
Antimicrobials					
Lactacin F ABC transporter	2.11–6.89	−0.1990	0.3161	−**0.7407**	**0.0267**
*creA*; colicin E2 tolerance	2.11–6.89	0.6291	0.9586	**0.8456**	**0.0057**
*creD*; colicin E2 tolerance	2.11–5.85	−**0.8424**	**0.0102**	−0.6547	0.1249
Membrane protein involved in colicin uptake	0.00–5.84	0.4838	0.8937	−**0.7154**	**0.0351**
Polyketide synthase module	2.11–5.85	−**0.8333**	**0.0118**	−**0.8804**	**0.0142**
Non-ribosomal peptide synthetase module	0.00–5.85	−**0.8319**	**0.0031**	0.3689	0.3342
	2.11–5.85	−**0.8154**	**0.0152**	−0.2571	0.5839
Thioesterase domains of type I polyketide synthases	0.00–5.85	−0.6147	**0.0464**	**0.8561**	**0.0047**
	2.11–5.85	−**0.8424**	**0.0102**	−0.6547	0.1249
Yersiniabactin non-ribosomal peptide synthetase	2.11–6.89	−**0.7386**	**0.0139**	−0.2790	0.4712
	2.11–5.85	−**0.8638**	**0.0070**	−0.6983	0.0946
Yersiniabactin non-ribosomal peptide/polyketide synthase	0.00–6.89	−**0.7000**	**0.0099**	−0.5854	0.0622
	0.00–5.85	−**0.8408**	**0.0026**	—	—
	2.11–5.85	−**0.9383**	**0.0008**	−**0.9856**	**0.0002**
Yersiniabactin synthetase	2.11–5.85	−**0.8424**	**0.0102**	−0.6547	0.1249
	2.11–6.89	−**0.7811**	**0.0077**	−0.5774	0.1106
	2.11–8.51	−0.6897	**0.0026**	−0.4477	0.1012
Yersiniabactin salicyl-AMP ligase	2.11–6.89	−**0.7811**	**0.0077**	−0.5774	0.1106
	2.11–5.85	−**0.8424**	**0.0102**	−0.6547	0.1249
	2.11–8.51	−0.6897	**0.0026**	−0.4477	0.1012
Mycobactin salicyl-AMP ligase	0.00–5.85	−0.2261	0.2926	**1.0000**	**<0.0001**
	2.11–5.85	−**0.8424**	**0.0102**	−0.6547	0.1249
	2.11–8.51	−0.5132	**0.0286**	0.1061	0.7085
Oligoketide cyclase/lipid transport protein Predicted thioesterase involved in non-ribosomal peptide biosynthesis	2.11–5.85	−**0.8424**	**0.0102**	−0.6547	0.1249
	0.00–6.89	−**0.7430**	**0.0053**	−**0.8078**	**0.0034**
	0.00–5.85	−**0.7511**	**0.0118**	−0.3436	0.3704
	2.11–6.89	−**0.7811**	**0.0077**	−0.5774	0.1106
	2.11–5.85	−**0.8424**	**0.0102**	−0.6547	0.1249
Type 6 secretion system	7.00–9.22	−0.3975	0.1598	−**0.7731**	**0.0180**
Ribosomal RNA small subunit methyltransferase E	2.11–5.85	−**0.7014**	**0.0483**	−0.2571	0.5839
rRNA small subunit methyltransferase I	7.00–9.22	**0.7050**	0.9795	**0.7186**	**0.0340**
Streptomycin 3-*O*-adenylyltransferase	0.00–6.89	−0.6742	**0.0137**	−**0.7853**	**0.0052**
	0.00–5.85	−0.6750	**0.0277**	−0.3305	0.3899
Spectinomycin 9-*O*-adenylyltransferase	7.00–9.22	0.8248	0.9964	**0.7370**	**0.0279**
Translation elongation factor LepA	0.00–5.85	0.1102	0.6036	**0.8518**	**0.0051**
SsrA-binding protein SmpB	2.11–6.89	−**0.7811**	**0.0077**	−0.5774	0.1106
	2.11–5.85	−**0.8424**	**0.0102**	−0.6547	0.1249
	2.11–8.51	−0.6897	**0.0026**	−0.4477	0.1012
DedA protein	2.11–6.89	−0.4985	0.0983	−**0.7326**	**0.0293**
Anti-sigma B factor RsbT	0.00–5.85	0.1998	0.6846	**0.7339**	**0.0289**
Transcriptional regulator YkgA	2.11–5.85	−**0.8424**	**0.0102**	−0.6547	0.1249
ABC-type antimicrobial peptide transport system	7.00–9.22	0.7960	0.9939	**0.8264**	**0.0081**
Oxidative Stress					
Rubredoxin-NAD (+) reductase	2.11–6.89	−**0.7811**	**0.0077**	−0.5774	0.1106
	2.11–5.85	−**0.8424**	**0.0102**	−0.6547	0.1249
QorR	2.11–5.85	−**0.8424**	**0.0102**	−0.6547	0.1249
	0.00–5.85	−0.2261	0.2926	−**0.7367**	**0.0280**
Uncharacterized glutathione S-transferase-like protein	6.79–9.22	0.2190	0.0826	0.6208	**0.0034**
Alkyl hydroperoxide reductase	2.11–5.85	−**0.8892**	**0.0041**	−0.5218	0.2432
OT coproporphyrinogen III oxidase	2.11–6.89	−**0.8333**	**0.0031**	−0.6429	0.0682
	2.11–5.85	−**0.9678**	**0.0001**	−0.7143	0.0846
Glutamate-cysteine ligase	2.11–5.85	−**0.8923**	**0.0038**	−**0.9411**	**0.0034**
Paraquat-inducible protein A	0.00–6.89	0.7090	0.9913	**0.7570**	**0.0084**
Glutathionylspermidine amidohydrolase	6.79–9.22	0.2094	0.0881	0.5293	**0.0075**
CoA-disulfide reductase	0.00–5.85	0.7287	0.9843	**0.8429**	**0.0060**
Hydroxyacylglutathione hydrolase	7.00–9.22	0.2236	0.1126	−**0.7186**	**0.0340**
Iron-binding ferritin-like antioxidant protein	0.00–5.85	0.1037	0.7855	**0.9708**	**<0.0001**
Glutaredoxin-related protein	2.11–6.89	−0.5407	0.0770	−**0.7075**	**0.0381**
Oxidoreductase YihU	2.11–5.95	−**0.8424**	**0.0102**	−0.6547	0.1249
GshF	2.11–5.95	−**0.8424**	**0.0102**	−0.6547	0.1249
Hydrogen peroxide-inducible genes activator	7.00–9.22	−**0.7824**	**0.0076**	−**0.8836**	**0.0025**
	6.79–9.22	−**0.7433**	**0.0052**	0.9416	0.2619
Glutathione synthetase	0.00–6.89	0.5343	0.9480	**0.7028**	**0.0180**
	2.11–6.89	0.3819	0.9548	**0.7638**	**0.0203**
Radical SAM family heme chaperone	2.11–6.89	−**0.7811**	**0.0077**	−0.5774	0.1106
	2.11–5.85	−**0.8424**	**0.0102**	−0.6547	0.1249
Xanthosine/inosine triphosphate pyrophosphatase	0.00–6.89	−**0.7243**	**0.0070**	−**0.8081**	**0.0034**
Xanthosine/inosine triphosphate diphosphatase	0.00–6.89	−0.6499	**0.0181**	−**0.7173**	**0.0149**
	2.11–6.89	−**0.9638**	**0.0002**	–	–
Probable peroxiredoxin	2.11–6.89	−**0.7811**	**0.0077**	−0.5474	0.1106
	2.11–5.85	−**0.8424**	**0.0102**	−0.6547	0.1249
Organic hydroperoxide resistance transcriptional regulator	6.79–9.22	−0.3910	0.1286	0.3731	**0.0068**
Thiol:disulfide oxidoreductase	2.11–6.89	−**0.7811**	**0.0077**	−0.5774	0.1106
	2.11–5.85	−**0.8424**	**0.0102**	−0.6547	0.1249
	0.00–5.85	−0.2261	0.2926	**1.0000**	**<0.0001**
Osmotic stress					
Glycine betaine transporter OpuD	2.11–6.89	−**0.7639**	**0.0100**	−0.6001	0.0944
	2.11–5.85	−**0.7038**	**0.0474**	−0.5161	0.2491
Choline ABC transport system	2.11–6.89	−**0.7216**	**0.0170**	−0.6088	0.0886
	2.11–5.85	−**0.7414**	**0.0346**	−0.6983	0.0946
Glycine betaine ABC transport system	2.11–5.85	−**0.8498**	**0.0090**	−**0.8286**	**0.0297**
l-Proline glycine betaine ABC transport system permease	6.79–9.22	−0.3996	0.1229	0.4931	**0.0059**
YehW	2.11–5.85	−**0.8424**	**0.0102**	−0.6547	0.1249
Osmotically inducible protein OsmY	2.11–5.85	−**0.8424**	**0.0102**	−0.6547	0.1249
Glucans biosynthesis protein C	2.11–5.85	−**0.8424**	**0.0102**	−0.6547	0.1249
Glucans biosynthesis glucosyltransferase H	2.11–5.85	−**0.7298**	**0.0383**	−0.6983	0.0946
NdvA	0.00–6.89	−0.0691	0.4347	**0.9324**	**0.0005**
Acid tolerance					
Carbonic anhydrous	0.00–5.85	0.3307	0.7922	**0.7350**	**0.0285**
Ornithine aminotransferase	7.00–9.22	−**0.7900**	**0.0067**	−**0.7609**	**0.0211**
Glutamate transport ATP-binding protein	7.00–9.22	0.7719	0.9911	**0.7545**	**0.0228**
Iron acquisition					
Iron chelate uptake ABC transporter family permease	2.11–5.85	−**0.8424**	**0.0102**	−0.6547	0.1249
	2.11–6.89	−**0.7811**	**0.0077**	−0.5774	0.1106
Heat shock					
DnaJ	0.00–5.85	−0.6695	**0.0291**	**0.9767**	**<0.0001**
GrpE	7.00–9.22	0.6085	0.9514	**0.7425**	**0.0262**
HrcA	7.00–9.22	0.7567	0.9890	**0.7665**	**0.0196**
Ribosome-associated heat shock protein	2.11–6.89	−0.5922	0.0547	−**0.7326**	**0.0293**
Carbon starvation					
Adenylate cyclase	0.00–6.89	0.5411	0.9506	**0.7966**	**0.0042**
	0.00–5.85	0.6448	0.9637	**0.7617**	**0.0208**
	2.11–6.89	0.5415	0.9234	**0.7545**	**0.0228**
CRP/FNR family transcriptional regulator	2.11–5.85	−**0.9377**	**0.0008**	−**0.9429**	**0.0032**
Carbon starvation protein A	7.00–9.22	0.7533	0.9885	**0.7904**	**0.0142**
Starvation lipoprotein Slp	2.11–5.85	−**0.8424**	**0.0102**	−0.6547	0.1249
Aldose-ketose isomerase YihS	2.11–5.85	−**0.8424**	**0.0102**	−0.6547	0.1249
Cellobiose phosphorylase	2.11–5.85	−**0.7420**	**0.0344**	−0.0286	0.9521
Outer membrane sugar transport protein YshA	2.11–5.85	−**0.8424**	**0.0102**	−0.6547	0.1249
Transcriptional regulator SgrR	2.11–5.85	−**0.8424**	**0.0102**	−0.6547	0.1249
	0.00–5.85	−0.2261	0.2926	**0.8488**	**0.0054**
Various polyols ABC transporters	2.11–6.89	−**0.7259**	**0.0162**	−0.6429	0.0682
Universal stress response					
Universal stress protein D	0.00–5.85	0.1950	0.6804	**0.8851**	**0.0024**
Universal stress protein G	2.11–5.85	−**0.8424**	**0.0102**	−0.6547	0.1249
Cold shock response					
CspD	6.79–9.22	−0.4641	0.0846	0.5354	**0.0002**
Envelop stress					
Phage shock protein A	2.11–5.85	−**0.8769**	**0.0054**	−0.4638	0.3067
	0.00–5.85	−0.1500	0.3599	**0.7211**	**0.0331**
Psp operon transcriptional activator	2.11–6.89	−**0.7811**	**0.0077**	−0.5774	0.1106
	2.11–5.85	−**0.8424**	**0.0102**	−0.6547	0.1249
Outer membrane stress sensor protease Deg	2.11–6.89	0.7090	0.9804	**0.8539**	**0.0049**
Outer membrane protein H precursor	2.11–6.89	−**0.7811**	**0.0077**	−0.5774	0.1106
	2.11–5.85	−**0.8424**	**0.0102**	−0.6547	0.1249
Sigma factor RpoE-negative regulatory protein RseA	0.00–5.85	0.4320	0.8628	**0.7633**	**0.0203**

^1^Pearson or Spearman correlation analysis was performed for enzyme abundance versus *Salmonella* abundance for 598 enzyme transcripts from MG-RAST KO metabolism, stress, or virulence data sets. Results are organized into metabolism (carbohydrate utilization; fermentation; TCA; respiration; or amino acid/nitrogen metabolism) or stress response (antimicrobials; oxidative stress; osmotic stress; acid tolerance, iron acquisition; heat shock; carbon starvation; universal stress response; cold shock or envelop stress) categories. Of all enzyme transcripts, 171 correlated with *Salmonella* abundance (*p* < 0.05), whereas 52 correlated with chicken age (*p* < 0.05, not shown). ^2^qPCR was used to estimate *Salmonella* abundance per gram cecal content (Pedroso et al., 2021). ^3^Significant *r*- or *ρ*-values > 0.6999 for Pearson or Spearman, respectively, or *p* < 0.05 are bold.

Most cecal transcripts identified as propionate kinase, methyl-malonyl CoA mutase, propionyl-CoA synthase, acetyl-CoA hydrolase, lactate dehydrogenase, or acetyl-CoA synthetase had 98–100% identity to *Escherichia coli* at the nucleotide level (Supplementary Table S8). The identities of other genera within the Enterobacteriaceae were all <98% nucleotide identities. However, some of these same transcripts had 98–100% identity, at the nucleotide level, to *Enterococcus faecium*, *Flavonifractor plautii*, or *Clostridia*. Some butyryl-CoA dehydrogenase and propionyl-CoA carboxylase enzyme transcripts had >98% nucleotide identity with *Clostridia* yet to be given a genus or species designation. However, there were many enzyme transcripts with little to no homology to any nucleotide sequences in BLAST but contained pfam domains characteristic of these enzymes. Pyruvate oxidase transcripts were primarily from *Enterococcus* or *Lactobacillus* species.

Analysis of the volatile fatty acid profile of the cecal contents of two birds with high *Salmonella* abundance showed variability. One sample had high acetate levels relative to the other VFAs, whereas another had high propionate and butyrate concentrations (Table 4). Therefore, in this small sample set, the volatile fatty acid profile and abundance did not correlate with the community transcriptome associated with fermentation.

**Table 4:** Volatile fatty acid composition of cecal contents of chickens colonized with *Salmonella.*

Volatile fatty acid	8.51 Log_10_ genomes/g^1^		7.00 Log_10_ genomes/g^1^	
	Weight (μg)	Mole %	Weight (μg)	Mole %
Acetate	13.6	2.7	146.8	91.0
Propionate	325.3	51.5	5.2	2.6
Butyrate	325.0	43.3	15.0	6.4
Valerate	21.0	2.4	0.0	0.0
Caproate	0.0	0.0	0.0	0.0
Decanoate	0.0	0.0	0.0	0.0
SUM	684.9	100.0	167.1	100.0

^1^*Salmonella* abundance.

### Cecal community transcripts that correlate with *Salmonella* abundance *in vivo*

Carbohydrate analysis revealed that arabinose and glucose were the most abundant sugars present in the cecal lipid fraction (Table 5), with varying levels of rhamnose, fucose, xylose, glucuronic acid, galacturonic acid, galactose, N-acetyl galactosamine, and N-acetyl glucosamine in the lipid or precipitate fractions of both groups. However, mannose was present in the cecal contents of birds with high *Salmonella* abundance. The cecal community transcriptome contained many enzyme transcripts associated with liberating the sugars from complex carbohydrates, including transcripts annotated as: α-l-arabinofuranosidase, arabinogalactan endo-β-1,4-galactanase, β-fructofuranosidase, β-fructosidase, α-fucosidase, β-N-acetylhexosaminidase, β-hexosaminidase, α,β-galactosidases, α,β-glucosidases, α-mannosidase, α-glucuronidase, β-xylosidase, glycosidases, endoglucanase, cellulase, chitinase, and xylanases. Sialidase was present as a rare transcript in the cecal transcriptome. The transcriptome also contained various enzyme transcripts for transport and channeling of these sugars into central catabolic pathways. Twenty-four enzyme transcripts associated with carbohydrate utilization, out of 70 analyzed, had a significantly positive or negative correlation with *Salmonella* abundance as determined by Pearson or Spearman correlation coefficients (*p* < 0.05), and 5 and 12 of these enzyme transcripts correlated with high or low *Salmonella* abundance, respectively (Table 3). The enzymes α-galactosidase, l-fuculokinase, and rhamnulokinase exhibited a significant (p < 0.05) positive correlation with high *Salmonella* abundance (7.00–9.22 Log_10_ *Salmonella* genomes/g cecal contents), while arabinogalactan endo-4,4-β-galactosidase, ribose/xylose/arabinose/galactoside ABC-type transport, ABC-type maltose transport system, β-hexosaminidase, and dehydro-3-deoxyphosphogluconate aldolase were positively correlated with *Salmonella* low abundance (<6.00 Log_10_ *Salmonella* genomes/g cecal contents; p < 0.05). There was also a significant positive correlation with fucose isomerase and *Salmonella* abundance (2.11–9.22 Log_10_ *Salmonella* genomes/g, cecal content). Most fuculokinase (*fucK*) transcripts were identified as *E. coli* transcripts, while l-fucose isomerase (*fucI*) transcripts had >98% sequence identity with *Anaerotruncus colihominis* or *Clostridium* sp. *M62/1* (Supplementary Table S8).

**Table 5:** Carbohydrate composition of cecal contents from chickens colonized with *Salmonella.*

Sugar	5.85 Log_10_ genomes/g^1^			7.20 Log_10_ genomes/g^1^		
	Free^2^	Lipid^2^	Precipitate^2^	Free^2^	Lipid^2^	Precipitate^2^
Arabinose	0.0	40.2	24.2	0.0	32.7	32.5
Rhamnose	0.0	3.9	0.0	0.0	8.0	4.3
Fucose	0.0	2.7	2.7	0.0	0.7	2.1
Xylose	0.0	14.8	19.4	0.0	6.0	15.9
Glucuronic acid	0.0	2.0	0.0	0.0	9.3	Trace
Galacturonic acid	0.0	1.0	2.5	0.0	0.3	1.9
Mannose	0.0	1.6	1.7	3.9	2.4	1.7
Galactose	31.0	17.6	11.1	26.2	16.9	29.4
Glucose	69.0	11.9	29.8	62.9	19.4	10.9
N-acetyl galactosamine	0.0	0.0	0.0	0.0	1.6	0.0
N-acetyl glucosamine	0.0	4.2	2.8	0.0	2.7	1.3
N-acetyl mannosamine	0.0	0.0	0.0	0.0	0.0	0.0

^1^*Salmonella* abundance. ^2^Fractions.

Cytochrome bd type quinol oxidase and cytochrome o ubiquinol oxidase had a significant positive and negative correlation, respectively, with *Salmonella* abundance. Nickel-dependent hydrogenase had a positive correlation with *Salmonella* abundance from 2.11 to 9.22 Log_10_ *Salmonella* genomes/g cecal contents (*p* < 0.05) (Table 3). The majority of the enzyme transcripts associated with respiration were from *E. coli*, with a few identified as *Salmonella enterica* (*hybD*, *narI*) (Supplementary Table S8).

Finally, a detailed analysis of the cecal community transcriptome focused on amino acid metabolism and *Salmonella* abundance identified 43 enzyme transcripts associated with amino acid/nitrogen transport or metabolism and *Salmonella* abundance (*p* < 0.05 by Pearson or Spearman) (Table 3). These transcripts were responsible for arginine, aspartate, glutamate, glutamine, glycine, histidine, phenylalanine, threonine, tryptophan, and tyrosine metabolism. Five (ABC-type polar amino acid transport system; periplasmic component/domain involved in amino acid transport; isopropylmalate/homocitrate/citramalate synthases; acetylglutamate kinase; and glutamate 5-kinase) and 10 enzyme transcripts (glutamate dehydrogenase; 5-enolpyruvylshikimate-3-phosphate synthase; allophanate hydrolase; aspartate-semialdehyde dehydrogenase; glutaminase; imidazolonepropionase; arginine/lysine/ornithine decarboxylases; threonine efflux protein; phosphoribosylanthranilate isomerase; and uncharacterized protein involve in cysteine biosynthesis) from cecal transcriptomes with low *Salmonella* levels had positive or negative correlations, respectively, with *Salmonella* abundance (*p* < 0.05), while seven enzyme transcripts (transglutaminase-like enzyme; tryptophanase; anthranilate phosphoribosyltransferase; anthranilate/para-aminobenzoate synthase; di- and tripeptidases; glutamine synthetase; and histidine ammonia-lyase) had a significantly positive correlation with high *Salmonella* abundance. Enzyme transcripts that focused primarily on glutamate metabolism (*glt*, *gadB*, *gadC*, *gabT*, and *gabD*) were from *E. coli*, *Enterococcus*, *Lactobacillus*, *Clostridia*, and unknown organisms (Supplementary Table S8).

### *Salmonella* exclusion by the intestinal community *ex vivo* was not due to scavenger metabolism and competition

**Figure 10:**
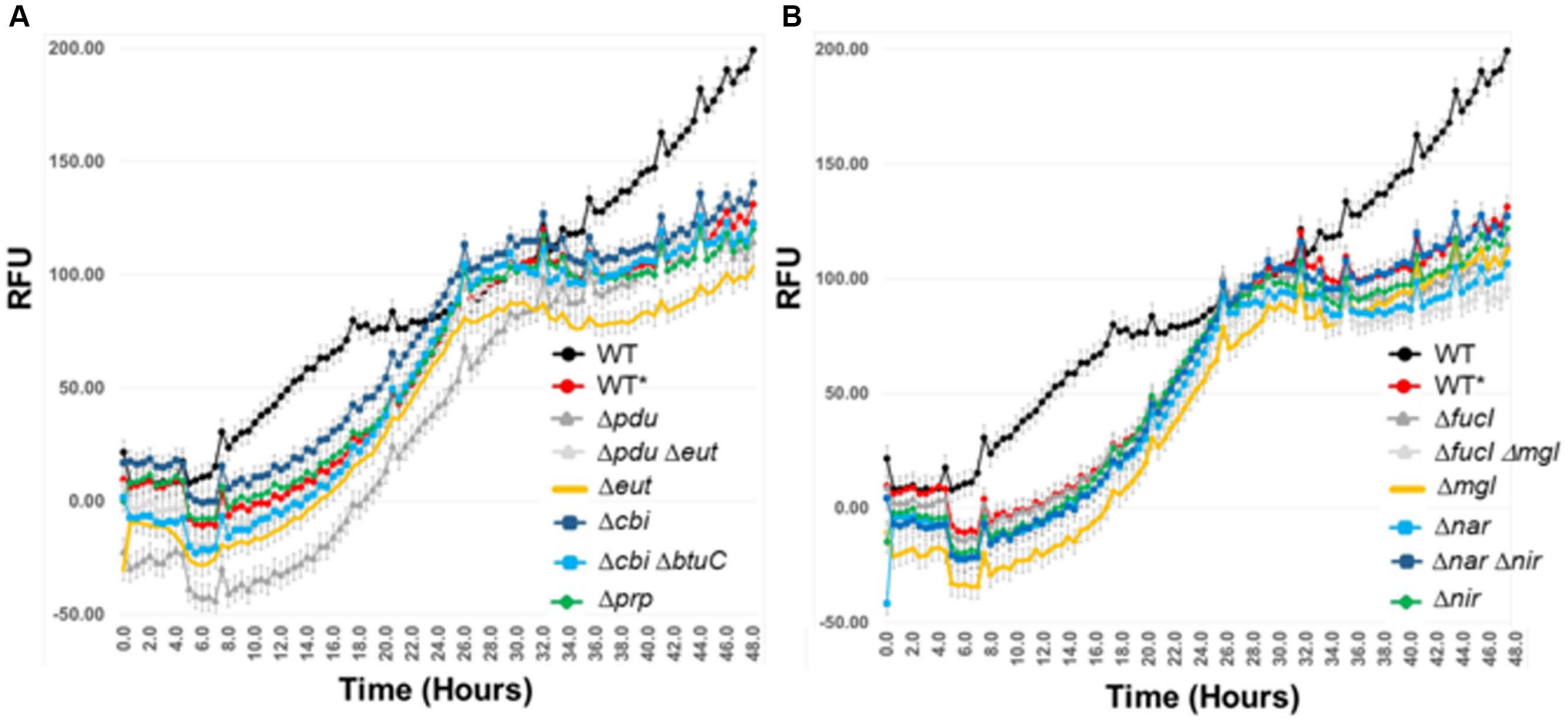
The effect of mutations in one or more metabolic pathways involved in *Salmonella* catabolism in an *ex vivo* intestinal environment containing an exclusive community. λ Red was used to create single and double mutations in *Salmonella* SL1344. pGLOW, a fluorescent reporter that fluoresces under aerobic and anaerobic conditions, was used to monitor *Salmonella* growth over 48 h. Mutant and wild-type (WT*) *S. typhimurium* SL1344 strains were grown in EVCC with exclusive community; WT was grown in EVCC alone as a control. Oxyrase (Sigma-Aldrich; St. Louis, MO) was added to the medium and overlaid with mineral oil to create and maintain low oxygen conditions. The selected mutations affect the catabolism of: **(A)** microbial metabolites (*eut*, *pdu*, *prp*), vitamin B12 synthesis (*cbi*), vitamin B12 uptake (*btuC*), or **(B)** sugar utilization (*fucI*, *mgl*) or anaerobic respiration (*nar*, *nir*).

Single and double mutations were examined in *Salmonella* that affected vitamin B12 synthesis (Δ*cbi*) or transport (Δ*btuC*); propanediol (Δ*pdu*) and ethanolamine utilization (Δ*eut*); propionate metabolism (Δ*prp*), fucose (Δ*fucI*) metabolism; anaerobic respiration (Δ*nar* or Δ*nir*), or glucose/galactose (Δ*mgl*) transport (Figure 10). A fluorescent reporter that functions under anaerobic conditions was used to monitor *Salmonella* growth over time. The *Salmonella* wild-type strain had a longer growth lag in co-culture with the exclusive community, and its growth plateaued sooner compared to growth in EVCC medium alone. At 18.5- to 26-h incubation, the exponential phase growth rate, as determined from the slope of the line, was the same for the *Salmonella* wild-type and most metabolic mutants when grown with the exclusive community (m = 4.27 + 0.04; range 4.04–4.57). However, the *Salmonella* Δ*pdu* single and Δ*cbi*, Δ*btuC* double mutants had slower (3.84) or faster (4.76) growth rates, respectively, compared to the wild type (4.23; Student’s *t*-test *p* < 0.05), when grown with the exclusive community. Biphasic growth was observed over the 48-h incubation, but *Salmonella* growth was significantly retarded in co-culture with the exclusive community during the last 8 h (*m* = 4.27 vs. 1.35). The biphasic growth exhibited by *Salmonella* wild type in EVCC medium alone exhibited a slower growth rate in the first phase (*m* = 2.74) compared to the second phase (*m* = 3.14). These results using mutants deficient in scavenger metabolism, anaerobic respiration, and multiple carbohydrate utilization indicate that *Salmonella*’s metabolic versatility augments its ability to compete for nutrients with an exclusive community.

### The role of antagonism in the mechanism of competitive exclusion

**Figure 11:**
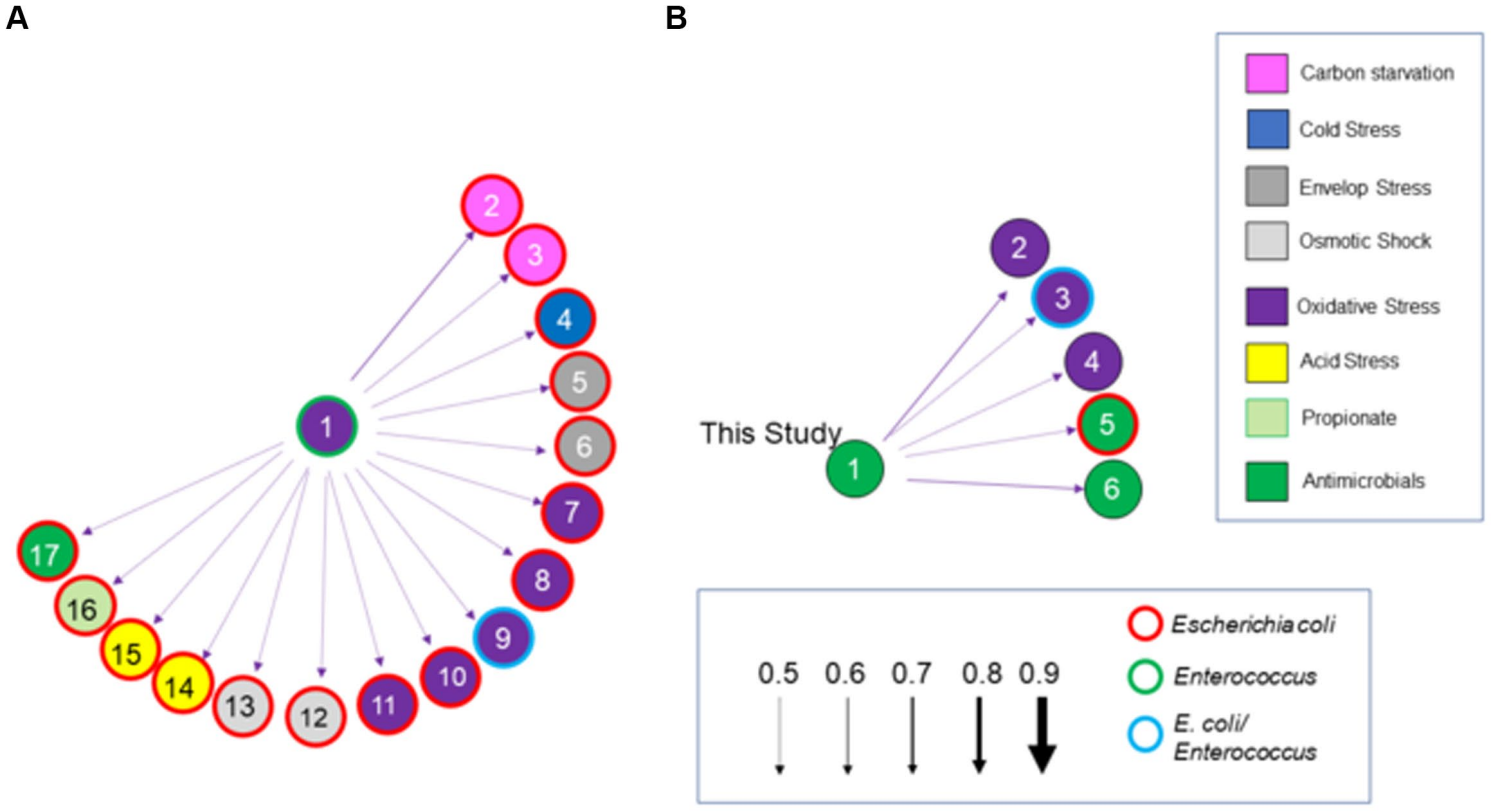
A Bayesian network analysis of the cecal transcriptome, focused on stress response, from chickens with high (>5.85 Log_10_ *Salmonella* genomes/g) **(A)** or low **(B)** *Salmonella* abundance. The network depicted was constructed using the score-based learning algorithm Hill-Climbing (arrows with solid line). Arrows indicate the direction and strength of the connection. Enzymes are color-coded based on the stress response. Enzymes or genes identified in the high *Salmonella* abundance community **(A)** are: (1) organic hydroperoxide resistance protein (*ohr*); (2) universal stress protein E (*uspE*); (3) *yciT*; (4) *cspI*; (5) *pspC*; (6) *rseA*; (7) *arcA*; (8) glutaredoxin; (9) *nrd*; (10) *yncG*; (11) *fmrR*; (12) *betI*; (13) *yehZ*; (14) *ybaT*; (15) *hdeA*; (16) propanediol diffusion facilitator; (17) glutamate-1-semialdehyde aminotransferase. Enzymes or genes identified in the low *Salmonella* abundance community **(B)** are: (1) yersiniabactin non-ribosomal peptide synthase; (2) putative bacterial hemoglobin; (3) *gshF*; (4) *hemW*; (5) *tehB*; (6) thioesterase domains of type I polyketides. Circles with thick red, green, or blue borders are for enzymatic transcripts with 98–100% nucleotide identity, by BLAST scores, to *Escherichia coli*, *Enterococcus* spp. or both, respectively.

*Salmonella ex vivo* transcripts in response to both communities were elevated for 34 of the total 161 genes associated with the stress response (Figure 8), compared to their growth in EVCC alone. Approximately 70% of the response was associated with heat shock, acid tolerance, and antimicrobials. There was also a significant difference in the *Salmonella* stress response in the exclusive community, where transcription of 32 genes was increased compared to the permissive community. One-third of these genes were associated with response to antimicrobials (Figure 7). In a network analysis of the cecal community transcriptomes’ stress response, a pattern emerged between cecal communities with high versus low *Salmonella* abundance (Figure 11). Oxidative stress appeared to be a major stress response in both cecal communities, but none of these or other stress response transcripts were shared, indicating that the stressors experienced in the communities were different. This contrasts with the results from the fermentation network analysis, where 30 enzyme transcripts were shared between *Salmonella* abundance groups and network analyses. For the high *Salmonella* abundance group, the organic hydroperoxide resistance protein (*ohr*) was the central node linked to various enzymes involved in carbon starvation, cold stress, envelope stress, osmotic stress, and acid tolerance. It also connected with glutamate-1-semialdehyde aminotransferase, an enzyme with a role in antibiotic synthesis. Yersiniabactin non-ribosomal peptide synthase was the major node in the stress response network for the cecal community with low *Salmonella* abundance (Figure 11). Forty-one of the enzyme transcripts from the two cecal communities were identified as *E. coli* or *Enterococcus* transcripts (>98% nucleotide identity with >99% coverage by BLAST) (Figure 11; Supplementary Table S8). There was a significantly negative correlation between transcript and *Salmonella* abundance for 72.5% of the enzyme transcripts associated with cecal transcriptomes’ stress response (Table 3). Several of these transcripts were associated with polyketide synthesis and annotated as: polyketide synthase module; thioesterase domains of type I polyketide synthases; Yersiniabactin non-ribosomal peptide synthase, non-ribosomal peptide/polyketide synthase, synthetase, and salicyl-AMP ligase; mycobactin salicyl-AMP ligase; oligoketide cyclase/lipid transport protein; and predicted thioesterase involved in non-ribosomal peptide biosynthesis. For yersiniabactin non-ribosomal peptide synthase, a few transcripts displayed a significant nucleotide identity with *E. coli* and *Klebsiella pneumoniae*. These sequence matches were identified as polyketide synthases *irp* responsible for producing the iron-scavenging yersiniabactin siderophore.

Several of the antimicrobial transcripts with a positive correlation to *Salmonella* abundance were also associated with antimicrobial resistance, such as colicin E2 resistance *creA*; rRNA small subunit methyltransferase I (*rsmI*); spectinomycin 9-O-adenylyltransferase [*ant (9)*]; and translation elongation factor *lepA*. The *creA* transcript was from *E. coli* (≥98% nucleotide identity; ≥99 coverage by BLAST). The aminoglycoside resistance gene *ant (9)* was commonly associated with a variety of diverse bacterial species with near-identical nucleotide sequences, including spectinomycin-resistant *Bacteroides*, *Campylobacter jejuni*, *Lactobacillus crispatus*, *Clostridioides difficile*, and *Clostridium* sp. *C1*. Additionally, the following antimicrobial resistance genes were identified in the chicken cecal transcriptome: *aadA1*, *spw*, *erm(B)*, and *ermG*. The latter two antibiotic resistance genes were from many bacterial species, including those commonly inhabiting the gastrointestinal tract (Supplementary Table S8). The *lepA* and *rsmI* enzyme transcripts had identity to sequences in *E. coli*, *Enterococcus* sp., various *Clostridia*, and unknown bacteria.

These results indicate that many of the members of the intestinal bacterial community were expressing resistance to antimicrobial compounds such as bacteriocins and antibiotics; however, there were no exogenous antimicrobials administered to the birds. The polyketide pathways suggest that some members of the microbiota may have been secreting antimicrobials that modulated the bacterial community, indicating the role of antagonism in competitive exclusion.

## Discussion

### An exclusive bacterial community attenuates the expression of virulence

These experiments illustrated that the chicken intestinal microbiota can significantly modulate *Salmonella*’s pathogenic behavior, indicating that attenuation may be involved in competitive exclusion. This suppression may be due to microbial metabolites like butyrate (Zhang Z.J. et al., 2020) or indole (Kohli et al., 2018) being shown to repress the SPI-1 T3SS cell invasion locus. Butyrate was not likely the main repressor, as this pathway was expressed in the cecal transcriptome of chickens with low and high *Salmonella* abundance, and there were differences in butyrate levels in chickens with high *Salmonella* abundance. Tryptophanase, the enzyme that liberates indole from tryptophan, was also present in the cecal transcriptome and directly proportional to *Salmonella* levels in the cecum, suggesting indole as a likely modulator of *Salmonella* virulence by the intestinal community. However, repression of SPI-1 T3SS by the exclusive community may be indirect, as this community also slowed *Salmonella* growth *ex vivo*, and growth rate is important in regulating the cell invasion of T3SS (Lee and Falkow, 1990). In addition, the exclusive community turned on *avrA*, a gene within the SPI-1 locus that lowers inflammation (Jones et al., 2008) and restores intestinal barrier integrity (Liao et al., 2008). It is the combination of SPI-1 T3SS repression and *avrA* activation that is responsible for *Salmonella*’s reduced virulence in the presence of an exclusive community. Others have shown repression of an enteropathogen’s virulence by the intestinal community or its member species (Le Bihan et al., 2015; Girinathan et al., 2021; Saenz et al., 2023).

In avian and mammalian species, juveniles are more likely to exhibit disease symptoms than adults when infected with *Salmonella* (Barrow and Methner, 2013). Day-of-hatch chicks may succumb to *Salmonella* infection after challenge (Bythwood et al., 2019), while 2-day-old birds will not. Instead, they shed high levels of *Salmonella* for 2–3 weeks before there is a precipitous drop in *Salmonella* shedding at approximately 3 weeks of age (Cheng et al., 2015; Pedroso et al., 2021). This suggests that the hatchling’s intestinal community is not effective in reducing the expression of *Salmonella* pathogenic behavior, but the microbiota rapidly gains this ability or at least counters inflammation induced by the pathogen (Yu et al., 2023). The exclusive community repressed LPS modification by the *pmr* locus in *Salmonella*, which is associated with resistance to cationic antimicrobial peptides (Matamouros and Miller, 2015). The host’s intestinal surface expresses β-defensins, a group of cationic antimicrobial peptides toxic to *Salmonella* (Milona et al., 2007). The exclusive community repressed *pmr* and therefore would make *Salmonella* susceptible to these and other defensins. While this early community can reduce the pathogen’s virulence early in the chick’s life, it’s not sufficient to exclude the pathogen from the intestinal luminal environment. The mechanism of competitive exclusion must involve more than suppression of the pathogen’s virulence in its mode of action.

### Competition did not overcome *Salmonella*’s metabolic versatility

*Salmonella* appeared to spend less energy toward anabolism when grown in the permissive community compared to the exclusive community. There was a substantial difference in transcript abundance for amino acid metabolism and peptide transport, nucleotide biosynthesis, and vitamin B12 uptake between *Salmonella* grown in these two communities. There were also differences in cecal community transcript abundance in the comparison of enzymes involved in amino acid metabolism. These findings suggest substantial differences in the availability of free amino acids, nucleotides, and vitamins within permissive and exclusive communities. Low availability of these nutrients in an exclusive community should result in *Salmonella*’s slow growth because of the energy cost of *de novo* synthesis (Ushijima and Seto, 1991; Ha et al., 1994; Yang et al., 2016, 2017).

However, the diverse sugars available for *Salmonella* utilization were reflected in the glycome analysis of the cecal contents of birds with high *Salmonella* abundance. In addition, the cecal community transcriptome displayed a diversity of glycosyl hydrolases that would liberate these sugars from complex carbohydrates and glycoproteins. Carbohydrate metabolism appeared to be central to *Salmonella* growth, as revealed by the array of *Salmonella* enzyme transcripts associated with catabolism and the significant number of differentially expressed genes subject to catabolite repression. This concurs with the finding that mutations in *crp* and *cya* attenuate *Salmonella* (Curtiss and Kelly, 1987) and other intestinal pathogens (Peighambari et al., 2002; Sun et al., 2010; Zhou et al., 2020). The *ex vivo* transcriptome revealed that the exclusive community appeared to contain fewer sugars but more fermentation end products, such as propanediol, for *Salmonella* growth. Network connections and cecal community enzyme transcript abundance relative to *Salmonella* levels indicated that propanediol may be linked to *Salmonella* growth through community cooperation. Transcriptional analysis of *Salmonella* grown anaerobically *ex vivo* in the exclusive community showed abundant respiration transcripts, suggesting that it was involved in growth on a poor energy source such as 1,2-propanediol. Propanediol utilization has previously been shown to be a contributor to early *Salmonella* colonization in mice and chickens; however, these studies were done with animals lacking a mature intestinal microbiota (Harvey et al., 2011; Faber et al., 2017). These results suggest that cooperation may be involved in promoting *Salmonella* growth in cecal communities.

Several metabolic genes were identified that were differentially regulated in the cecal communities with high or low *Salmonella* abundance. Single and multiple deletions were introduced into these genes to assess their contribution to *Salmonella* growth in the exclusive community. The genes *pdu*, *cbi*, and vitamin B12 receptors are all necessary for propanediol metabolism, and they were transcribed during the *ex vivo* growth of *Salmonella* with the cecal communities. Only the *Salmonella* Δ*pdu* single mutation significantly reduced *Salmonella* growth rates compared to wild type. There were no significant growth defects between the other mutants and wild type in the presence of the exclusive community. This finding suggests that, when presented with a diverse array of nutrients, *Salmonella* is metabolically versatile and adapts to the changing availability of nutrients in the intestine. Its metabolic versatility is reflected in the different metabolic genes shown to be involved in the colonization of different animal and plant species (Kwan et al., 2018). Therefore, neither cooperation nor competition appears to be the sole mechanism behind competitive exclusion.

### Cecal communities may exhibit antagonism via the production of antimicrobials

The network analysis of the cecal stress response revealed that a polyketide synthase was the central node linked to similar enzymes in the cecal community containing low *Salmonella* abundance. With a few exceptions, these enzyme transcripts could not be assigned to known intestinal species in the NCBI database but exhibited similarity to as yet to be identified clostridial species. Most polyketide synthase modules responsible for antibiotic synthesis have been found in aerobic soil microbes because much research has focused on these organisms (Behnken and Hertweck, 2012a). However, recent studies have identified potential antimicrobial polyketides in *Clostridia* (Seedorf et al., 2008; Behnken and Hertweck, 2012b; Li et al., 2020), and polyketide synthases have been identified within the human intestinal microbiome (Fritz et al., 2018). Clostridial polyketide synthase may also be involved in siderophore synthesis (Singh et al., 2017). There was a negative correlation between transcript abundance for many of these enzymes and *Salmonella* levels in the cecum, as well as other enzyme transcripts associated with antibiotic and colicin resistance. This finding suggests that the exclusive community was likely producing antimicrobial substances. In addition, transcripts annotated as resistance genes with homology to many intestinal species, including *Salmonella* and *E. coli*, were detected, indicating the expression of antibiotic resistance. The chickens used in this study were reared without any antibiotics; therefore, the *in vivo* detection of antimicrobial resistance gene expression was not due to iatrogenic usage. Alternatively, the contact-dependent antibacterial type 6 secretion system (T6SS) (Alteri et al., 2013) might be at play in *Salmonella* exclusion. The *ex vivo* experiment would have precluded the detection of this mechanism. However, *E. coli* T6SS transcripts were present in the cecal transcriptomes, demonstrating the strength of a combined *ex vivo*/*in vivo* approach to unraveling the mechanism of competitive exclusion. While there was a negative correlation between the cecal T6SS transcript and *Salmonella* abundance, this is not the likely mechanism, as this correlation was only observed in chickens with high *Salmonella* colonization.

Early work demonstrated inhibition of *Salmonella* growth by cecal microbiota *in vivo* (Bohnhoff et al., 1964a; Royal and Mutimer, 1972), and pretreatment of mice with streptomycin reduced the inhibitory effect of the cecal microbiota on *Salmonella* colonization (Bohnhoff et al., 1964b). Others have looked at the ability of intestinal species to compete with bacterial pathogens for limiting resources (Barrow et al., 1987; Ushijima and Seto, 1991) or adherence to mucosal surfaces, indicating that competition was accepted as the mechanism of competitive exclusion (Gusils et al., 2003; Collado et al., 2005). However, the inhibitory effect on *Salmonella* growth was hypothesized to be related to volatile fatty acid (VFA) production and the potentiating effect of pH, suggesting that the role of inhibition was also considered (Bohnhoff et al., 1964b; Royal and Mutimer, 1972). This led to bio-prospecting for intestinal bacteria species for their production of inhibitory VFAs (Hinton et al., 1991). Antimicrobial production has also been used to select candidate probiotics; however, most have failed to reduce *Salmonella* colonization (Pan et al., 2008; Grant et al., 2018; Lu et al., 2023). There has also been considerable focus on microbes that produce bacteriocins (Grant et al., 2018; Angelopoulou et al., 2019; Khan et al., 2020). As an example, Wooley *et al*. demonstrated the inhibitory effects of a microcin-producing avian *E. coli* isolate on *Salmonella* colonization in chickens (Wooley et al., 1999). More recently, *Bacillus* spp. have been shown to be quite effective at excluding or controlling some intestinal pathogens in chickens (Grant et al., 2018). The genus *Bacillus* contains many member species containing antimicrobial polyketide synthases (*pks*) (Olishevska et al., 2019). *B. subtilis pks* has been shown to produce substances that inhibit *Salmonella* growth in co-culture (Podnar et al., 2022). It has been long established that prophylactic use of antibiotics, even at sub-therapeutic levels, can adversely affect *Salmonella* colonization in several animal species, including chickens (Sieburth, 1957; Evangelisti et al., 1975; Smith and Tucker, 1975). The logical next step would be to screen, isolate, and characterize presumptive antimicrobials from exclusive communities to demonstrate their effects on colonization.

## Conclusion

The mode of action of competitive exclusion in reducing *Salmonella* in chickens appears to involve a combination of competition, attenuation, and antagonism by member species in the cecal community. Studies have demonstrated the importance of microbial community diversity in pathogen exclusion (recently reviewed in Pedroso et al., 2021). There have been many studies seeking to identify one or several intestinal species that inversely correlate with *Salmonella* abundance in the chicken intestine in an effort to describe a defined competitive exclusion formulation. Except for the *Bacillus* commercial products, no single species has been shown to directly affect *Salmonella* abundance in chickens across multiple studies. Even the competitive exclusion product Aviguard^®^ exhibits considerable variability in community composition while retaining product efficacy for reducing *Salmonella* levels in chickens (Lee et al., 2023b). This diversity may reflect the multitude of intestinal species required to collectively affect *Salmonella*’s metabolic versatility and compete for the available resources in the intestine, reduce pathogenic behavior, or produce antimicrobials that inhibit *Salmonella*. Overlap of single functions may occur between different members of the collective community, rendering individual species dispensable. Alternatively, all three functions may be required for pathogen exclusion within the collective community, with a diversity of members necessary to achieve pathogen control.

## Data availability statement

The datasets presented in this study can be found in online repositories. The names of the repository/repositories and accession number(s) can be found in the article/Supplementary material.

## Ethics statement

The animal study was approved by the University of Georgia Animal Care and Use and Procedures Committee. The study was conducted in accordance with the USDA and institutional requirements.

## Author contributions

JM: Conceptualization, Data curation, Formal analysis, Funding acquisition, Investigation, Writing – original draft, Writing – review & editing. YC: Conceptualization, Formal analysis, Investigation, Methodology, Writing – review & editing. AP: Investigation, Methodology, Writing – review & editing. KT: Formal analysis, Investigation, Methodology, Writing – review & editing. SA: Data curation, Formal analysis, Investigation, Methodology, Writing – review & editing. TK: Investigation, Methodology, Writing – review & editing. GM: Formal analysis, Supervision, Writing – review & editing. SK: Data curation, Formal analysis, Writing – review & editing. SP: Data curation, Formal analysis, Investigation, Methodology, Validation, Writing – review & editing. MM: Conceptualization, Data curation, Formal analysis, Funding acquisition, Investigation, Writing – review & editing. JE-S: Methodology, Writing – review & editing. ML: Conceptualization, Funding acquisition, Investigation, Project administration, Supervision, Writing – review & editing.

## Footnotes

^1^ http://statweb.stanford.edu/∼tibs/SAM/
