# Supplementary material for: Peeling back the many layers of competitive exclusion
Source: Front Microbiol. 2024 Mar 21;15:1342887. doi: 10.3389/fmicb.2024.1342887 (PMC11000858; doi:10.3389/fmicb.2024.1342887)
Supplement: Supplementary file 1 [file Data_Sheet_1.PDF]

SUPPLEMENTAL

TABLE S1 Bacterial strains and plasmids

Bacterial strain or plasmid[1]	Description[2]	Reference
***E. coli* (K12)**		
CC118	Δ*(ara-leu)*, *araD*, Δ*lacX*74, *galE*, *galK*, *phoA*20, *thi*-1, *rpsE*, *rpoB*, *argE(Am)*, *recA*l, λ*pir*	(1)
SM10	*thi*-1, *thr*, *leu*, *tonA*, *lacY*, *supE*, *recA*::RP4-2-Tc::Mu, Kn^r, λ*pir*	(2)
***S.* Typhimurium**		
LT2		(3)
KKT01 (LT2)	pKD20	This Study
KKT02 (LT2)	Δ*fucI::cat;* Strain KKT01 transformed with PCR amplicon[3]	This Study
KKT04 (LT2)	Δ*nirB::cat*; Strain KKT01 transformed with PCR amplicon[3]	This Study
KKT05 (LT2)	Δ*btuC::cat*; Strain KKT01 transformed with PCR amplicon[3]	This Study
43	Poultry isolate	(4)
43 *mgl*	Δ*mgl::cat*	(4)
43 *prp*	Δ*prp::cat*	(4)
43 *nar*	Δ*nar::aph*	(4)
43R	Rifampicin-resistant (64 μg/ml) derivative of poultry isolate 43	(4)
JE16025	*metE2702*, *araB9*, Δ*cbiB-cobT4::aph*	This Study
JE8088	*metE205*, *araB9*, Δ*eutBC1156*::*cat*	(5)
JE8566	*metE205*, *araB9*, Δ*pduCDE512*::*cat*	This Study
SL1344	*hisG*	(6)
KKT07 (SL1344)	*hisG*, Δ*cbi::aph*; SL1344 transduced with P22 JE16025 lysate	This Study
KKT08 (SL1344)	*hisG*, Δ*cbi::aph*; pCP20; Strain KKT07 transformed with pCP20[4]	This Study
KKT09 (SL1344)[5]	*hisG,* Δ*cbi*	This Study
KKT10 (SL1344)	*hisG*, Δ*eut::cat*; SL1344 transductant with P22 JE8088 lysate	This Study
KKT11 (SL1344)	*hisG*, Δ*pdu::cat*; SL1344 transductant with P22 JE8566 lysate	This Study
KKT12 (SL1344)	*hisG*, Δ*pdu*::*cat*, pCP20; Strain KKT11 transformed with pCP20[4]	This Study
KKT13 (SL1344)[5]	*hisG, Δpdu*	This Study

KKT14 (SL1344)	*hisG*, Δ*mgl::cat*; SL1344 transduced with P22 43 *mgl* lysate	This Study
KKT15 (SL1344)	*hisG*, Δ*mgl::cat*; pCP20; KKT14 transformed with pCP20[4]	This Study
KKT16 (SL1344) [5]	*hisG*, Δ*mgl*	This Study
KKT17 (SL1344)	*hisG*, Δ*prp::cat*; SL1344 transduced with P22 43 *prp* lysate	This Study
KKT18 (SL1344)	*hisG*, Δ*prp::cat*; pCP20; KKT17 transformed with pCP20[4]	This Study
KKT19 (SL1344) [5]	*hisG*, Δ*prp*	This Study
KKT20 (SL1344)	*hisG*, Δ*nar::aph*; SL1344 transduced with P22 43 *nar* lysate	This Study
KKT21 (SL1344)	*hisG*, Δ*nar::aph*; pCP20; Strain KKT20 transformed with pCP20[4]	
KKT22 (SL1344) [5]	*hisG*, Δ*nar*	This Study
KKT23 (SL1344)	*hisG*, Δ*fucI::cat;* SL1344 transduced with P22 KKT02 lysate	This Study
KKT25 (SL1344)	*hisG*, Δ*nirB::cat*; SL1344 transduced with P22 KKT04 lysate	This Study
KKT26 (SL1344)	*hisG*, Δ*btuC::cat*; SL1344 transduced with P22 KKT05 lysate	This Study
KKT35 (SL1344)	*hisG, Δmgl, ΔfucI*::*cat*; Strain KKT16 transduced with KKT02 lysate	This Study
KKT36 (SL1344)	*hisG, Δmgl, ΔfucI*::*cat,* pCP20; Strain KKT35 transformed with pCP20[4]	This Study
KKT41 (SL1344) [6]	*hisG*, Δ*nar*, Δ*nirB*::*cat*; Strain KKT22 transduced with P22 KKT04 lysate	This Study
KKT43 (SL1344) [6]	*hisG,* Δ*cbi*, Δ*btuC::cat*; Strain KKT09 transduced with P22 KKT05 lysate	This Study
KKT44 (SL1344) [6]	*hisG,* Δ*pdu*, Δ*eut*::*cat*; Strain KKT13 transduced with P22 JE8088 lysate	This Study
KKT45 (SL1344) [6]	*hisG,* Δ*cbi*, pGLOW-K^{XN}-Bs2; Strain KKT09 transformed with pGLOW-K^{XN}-Bs2	This Study
KKT46 (SL1344) [6]	*hisG,* Δ*pdu*, pGLOW-K^{XN}-Bs2; Strain KKT13 transformed with pGLOW-K^{XN}-Bs2	This Study
KKT47 (SL1344) [6]	*hisG*, Δ*mgl*, pGLOW-K^{XN}-Bs2; Strain KKT16 transformed with pGLOW-K^{XN}-Bs2	This Study
KKT48 (SL1344)	*hisG*, Δ*prp*, pGLOW-K^{XN}-Bs2; Strain KKT19 transformed with pGLOW-K^{XN}-Bs2	This Study
KKT49 (SL1344)	*hisG*, Δ*nar*, pGLOW-K^{XN}-Bs2; Strain KKT22 transformed with pGLOW-K^{XN}-Bs2	This Study
KKT51 (SL1344)	*hisG*, Δ*nirB::cat*, pGLOW-K^{XN}-Bs2; Strain KKT25 transformed with pGLOW-K^{XN}-Bs2	This Study
KKT55 (SL1344)	*hisG, Δmgl, ΔfucI*, pGLOW-K^{XN}-Bs2; Strain KKT37 transformed with pGLOW-K^{XN}-Bs2	This Study
KKT60 (SL1344)	*hisG*, Δ*nar*, Δ*nirB*::*cat*, pGLOW-K^{XN}-Bs2; Strain KKT41 transformed with pGLOW-K^{XN}-Bs2	This Study

KKT62 (SL1344)	*hisG,* Δ*cbi*, Δ*btuC::cat*, pGLOW-K^{XN}-Bs2; Strain KKT43 transformed with pGLOW-K^{XN}-Bs2	This Study
KKT63 (SL1344)	*hisG,* Δ*pdu*, Δ*eut*::*cat*, pGLOW-K^{XN}-Bs2; Strain KKT44 transformed with pGLOW-K^{XN}-Bs2	This Study
YC1098 (SL1344)	*hisG*, pKD46	This Study
YC1099 (SL1344)	*hisG*, *iag-tetB*-transcriptional terminator, pKD20; YC1098 transformed with PCR amplicon[6]	This Study
YC1100 (SL1344)	*hisG*, *iag-cfp*-transcriptional terminator; YC1099 transformed with PCR amplicon[7]	This Study
YC1101 (SL1344)	*hisG*, P22 *att*::*rrnB* promoter-RBS-*yfp*, *cat;* YC1095 transformed with PCR amplicon[8]	This Study
YC1103 (SL1344)	*hisG*, P22 *att*::*rrnB* promoter-RBS-*yfp*, *cat;* SL1344 transduced with P22 YC1101 lysate	This Study
YC1104 (SL1344)	*hisG*, P22 *att*::*rrnB* promoter-RBS-*yfp*, *cat*, , *iag-cfp*-transcriptional terminator*;* YC1100 transduced with P22 YC1103 lysate	This Study
**Phage**		
P22	HT *int*; Generalized transducing phage	(7)
**Plasmids**		
pGP704	*ori* R6K, *mob* RP4, MCS of M13tgl31; Ap^r; Plasmid replication is dependent on Π protein supplied in trans in *E. coli* strains with λ*pir*. Plasmid can be mobilized in *E. coli* strains with RP4 (ex. *E. coli* SM10).	(2)
pCR-XL-TOPO	Plasmid for cloning 3-10 kb PCR amplicons; pUC origin; Km^r, Zeocin^r	Invitrogen
pROBE-gfp[LVA]	Gfp variant in broad host range plasmid with *oriV* and p15a *ori* for plasmid replication in *Escherichia coli*. Amino acid substation shortens the protein's half-life to 40 minutes. Gm^r/Km^r	(8)
pMG32	*yfp* (gfpmut3.1 V68L Q69K Q80R T203Y[9])	(9)
pMG34	*cfp* (gfpmut3.1 F64L G65T Y66W A72S Q80R N146I M153T V163A N164H[9])	(9)
pMG32T1T2	*SpeI*, *ApaI* digested *rrnB*T1T2 amplicon was cloned into same pMG32 restriction sites, downstream of *yfp* (*yfp-rnB*T1T2). *Salmonella* Typhimurium SL1344 served as template for *rrnB*T1T2 PCR.	This Study
pCRXL01	pCR-XL-TOPO with *yfp* with ribosome binding site (rbs) flanked by 5' ribosomal RNA P1 promoter (*rrnB*P1) and *rrnB*T1T2 (*rrnB*P1-rbs-*yfp-rrnB*T1T2). Plasmid pMG32T1T2 served as template in PCR using forward, *Sal*I engineered *rrnB*P1 primer, which contained 3' overlap with *yfp*, and the reverse rrnBT1T1 oligonucleotide with the engineered, 3' *Xba*I restriction enzyme site. Km^r, Zeocin^r	This Study
pCY01	pGP704 with *rrnB* promoter-RBS-*yfp-rrnB*T1T2. A 1.1 kb *Sal*I, *Xba*I DNA fragment from pCRXL01 was cloned into *Sal*I, *Eco*RV sites of pGP704. Ap^r	This Study

pCY02	pGP704 with *rrnB* promoter-RBS-*yfp*-λT0. *rrnB*T1T2 was replaced with λT0 transcriptional terminator by cloning λT0 248 bp amplicon into *Apa*I, *Spe*I site of pCY01. Plasmid pROBE-gfp[LVA] served as template in PCR using transcriptional terminator λT0 primers with engineered *SpeI*, *ApaI* sites for directional cloning. Ap^r	This Study
pCY03	pGP704 with *rrnB* promoter-RBS-*yfp*-λT0 transcriptional terminator, in tandem with pKD3 *cat* gene with flanking FRT sequences (*rrnB*P-rbs-*yfp-cat*). PCR product was generated using cat primers with engineered *Apa*I and *Eco*RV restriction sites in the forward and reverse primers, respectively using pKD3 as template and was cloned into *Apa*I, *Eco*RV restriction sites of pCY02. The resulting plasmid pCY03 served as PCR template for λ*red* insertion (10) into *S.* Typhimurium P22 integration site using P22 *att* site specific primers with nucleotide sequence overlap with *rrnB*P1 promoter and *cat*. Ap^r, Cm^r	This Study
pKD3	Template plasmid for *cat* cassette used in recombineering λ*red* mediated insertions and subsequent "flippase" mediated excisions/deletions; Ap^r, Cm^r	(10)
pKD4	Template plasmid for *aph* cassette used in recombineering λ red mediated insertions and subsequent "flippase" mediated excisions/deletions; Ap^r, Km^r	(10)
pKD20, pKD46	*repA101ts*, λ γ, β, *exo*; Ap^r	(10)
pCP20	Temperature-sensitive replicon and inducible "flippase" (Flp) for deleting *cat* and adjacent sequences to create targeted deletions; Ap^r, Cm^r	(11)
pGLOW-K^{XN}-Bs2	Engineered fluorescent protein YtvA from *Bacillus subtilis* with *Escherichia coli* codon usage for expression in Gram-negatives. Unlike the jellyfish GFP, YtvA fluoresces in the absence of oxygen. Cm^r, Km^r.	(12)

Ap^r- ampicillin resistance (25 μg/ml); Cm^r-chloramphenicol resistance (25 μg/ml); Gm^r-gentamicin resistance; Km^r-kanamycin resistance (50 μg/ml); Tc^r-tetracycline resistance (10 μg/ml). *aph*- kanamycin resistance gene; *cat*-chloramphenicol resistance gene; *tetA*- tetracycline resistance gene. [1]( ) *Salmonella* or *Escherichia coli* strain background. [2]Genotype and general description of how strain or plasmid was constructed. [3]Bacterial strains were transformed, by electroporation (13), with PCR products. Amplicons were generated with primers that target a metabolic gene (*fucI*, *nanA*, *nirB*, or *btuC*); using pKD3 (*cat*; chloramphenicol resistance) or pKD4 (*aph*; kanamycin resistance) as template (10) (Table S2). Transformants were selected by plating electroporated, bacterial cells on media with chloramphenicol or kanamycin. [4]Transformants (13) were selected by plating cells on LB agar with ampicillin (25 μg/ml) at 30°C (10). [5]"Flippase" on pCP20 was induced at 43°C. Resulting colonies were screened for loss of plasmid (ampicillin) and "cassette" encoded resistance (chloramphenicol or kanamycin) (10). [6]Amplicon was generated with primers that target the region between *iag* and transcriptional terminator, for *hilA* operon (Table S2); using Tn*10* (*tetR,B*; tetracycline resistance) as template. [7]Amplicon was generated with primers that target the region between *iag* and transcriptional terminator, for *hilA* operon (Table S2); using pMG34 (*cfp*) as template. Transformants were selected by plating electroporated, bacterial cells on media containing fusaric acid. Fusaric acid-resistant colonies were subsequently screened for tetracycline sensitivity (14). [8]Amplicon was generated with primers flanking *rrnB* promoter and FRT in pCY03 (Table S2). Transformants were selected by plating electroporated, bacterial cells on media containing chloramphenicol. [9]Amino acid substitution in gfpmut3.1 (Clonetech; Mountain View, CA) changing GFP fluorescence to yellow or cyan spectrum (9).

TABLE S2 PCR Primers

Gene	Sequence [1]	PCR Conditions [2]	Expected Size (bp)	Reference
Universal 16S	F:cggtgaatacgttcycgg	56.3, 2 mM	142	(15)
	R:ggwtaccttgttacgactt			
iagB (*tetR*)	F:gaagagaaaaacaaaagactttctatcgcggcaaacaaataattaAGACCCACTTTCACATT		1,907	This Study
sptP(*tetA*)	R:taaaaacatagcttacttttagaactatctgaaagtaagctatttctgtataaCTAAGCACTTGTCTCCTG			
iagB (*cfp*)	F:gaagagaaaaacaaaagactttctatcgcggcaaacaaataaCCTAGAATTAAAGAGGAGAA		857	This Study
sptB (*cfp*)	R:taaaaacatagcttacttttagaactatctgaaagtaagctatttctgtataaaGGTCAGCTAATTAAGCTTA			
*Sal*I-*rrnB*-P	F:catc***gtcgac***tcctcttgtcaggcagaaaataactccctataatgcgccaccactgacacggaacaacggcaggtacct agaaattaaagaggag		1,089	This Study
*Xba*I-*rrnB*T1T2	R:agcc***tctaga***ttacagacaagctgtgacc			
*Spe*I-*rrnB*T1T2	F:***actagt***agagtagggaactgccag		148bp	This Study
*Apa*I-*rrnB*T1T2	R:***gggccca***agagtttgtagaaacgc			
*Apa*I-FRT-*cat*	F:gactca***gggccc***GTGTAGGCTGGAGCTGCTTC		1,024	This Study
*Eco*RV-FRT-*cat*	R:cagcta***gatatc***GGTCCATATGAATATCCTCCTTAG			
*Pst*I-FRT-*cat*	F:gactca***ctgcag***GTGTAGGCTGGAGCTGCTTC		1,025	This Study
*Xho*I-FRT-cat	R:cagcta***ctcgag***GGTCCATATGAATATCCTCCTTAG			
*Spe*I-λT0	F: gactca***actagt***cttaattagctgagcttggac		248	This Study
*Apa*I-λT0	R:cagcta***gggccc***ttgagcaactgactgaaatg			
P22*att* (*rrnB*P1)	F:aggttcgactcctattatcggcaccatctaaatcaatcacTCCTCTTGTCAGGCAGAA		2,081	This Study
P22*att* (*cat*)	R:agcaaaaaatggtgtttttgagaaatgaggttgtacataaAAGAGTTTGTAGAAACG			

fucI λred	F:ccgaaaatcggtatccgcccggtgattgatggacgtcgtatgggcgtacgGTGTAGGCTGGAGCTGCTTC	60ºC[3]	1,200	This Study
fucI λred	R:ccgggatacgcagcatcgcggcgagagtaataaagtccgcccctacatggGGTCCATATGAATATCCTCCTTAG			
prpE λred	F:cgaatttgctgcaacgacgcgggatcgtcaatggttgtcGTGTAGGCTGGAGCTGCTTC	50ºC, 2 mM	1,179	(4)
prpB λred	R:cgagatatgtctttacattcgccggggcaggcatttcgcgGGTCCATATGAATATCCTCCTTAG			
btuC λred	F:ctgagcttatgcgcaggcgaacagtggattgcccccggtgactggttaagcGTGTAGGCTGGAGCTGCTTC	58ºC[3]	1,201	This Study
btuC λred	R:gttcagccgacgccagtgccagtcgggcgaccacatcagccaatagcaggGGTCCATATGAATATCCTCCTTAG			
narU λred	F:tgtgaggggtaaaatgacacgacaaaacgagaattataacGTGTAGGCTGGAGCTGCTTC	50ºC, 2 mM	1,681	(4)
narV λred	R:gggcaaagagaattagcggcgggtacgaacaatctggtagcGGTCCATATGAATATCCTCCTTAG			
nirB λred	F:cgacattaccgtgttctgtgaagaaccccgtaaagcctatgaccgtgtccaccGTGTAGGCTGGAGCTGCTTC	58ºC[3]	1,203	This Study
nirB λred	R:ggtactcgatgccgccttccagattatccagccacggcgcggtacgggtcGGTCCATATGAATATCCTCCTTAG			
mglC λred	F:gtctggataactacttcttacgcgcgtatttcagcgagtcGTGTAGGCTGGAGCTGCTTC	50ºC, 2 mM	1,180	(4)
mglB λred	R:ctaccatgaataagaaggtactgaccctttctgccgtgatGGTCCATATGAATATCCTCCTTAG			
eutB λred	F:atgaaactaaagaccacattgttcggcaatgtttatcagtttaaggatgtaGTGTAGGCTGGAGCTGCTTC			(5)
eutC λred	R:ttaacgggtcatgttgatgccggacgctttctgctccagcatccgtttGGCCATATGAATATCCTCCTTAG			

[1] Sequence in all CAPS and underlined represent 5' and 3' FRT sequences flanking *cat* and *aph* genes present on plasmids pKD3 and pKD4, respectively. The 5' under case sequence represents target gene sequences. Restriction enzyme recognition sites engineered into primer sequence are italicized with 5' sequence overhang allowing restriction enzyme digestion of PCR amplicon. [2] Annealing temperature and $MgCl_2$ concentration. [3]Platinum^{Tm} Taq DNA Polymerase High Fidelity (Invitrogen; Waltham, MA) using manufacturer's PCR mix which contains nucleotides, buffers and salts.

TABLE S3 *Ex vivo* chicken cecal medium[1]

Compound	Concentration	Manufacturer
Basal medium		
Mucin from porcine stomach (type III)	2.5 mg/ml	Sigma-Aldrich
Phytone peptone[2]	5.0 mg/ml	BD
KCl	0.37 mg/ml	J. T. Baker
$NaHCO_3$	0.42 mg/ml	J. T. Baker
NaCl	1.75 mg/ml	J. T. Baker
L-Cysteine HCl H_2O	0.69 mg/ml	Thermo-Fisher Scientific
Hemin	0.0001 mg/ml	Frontier Scientific
Resazurin	0.001 mg/ml	MP Biomedicals
Uric Acid	0.002 mg/ml	Sigma-Aldrich
Amino acid supplement		
L-Arginine HCl	0.94 mg/ml	Sigma-Aldrich
L-Isoleucine	0.60 mg/ml	Acros
L-Lysine HCl	0.89 mg/ml	Sigma-Aldrich
L-Methionine	0.50 mg/ml	Sigma-Aldrich
L-Threonine	0.52 mg/ml	Acros

[1]Modification of an intestinal medium formulation described by Ruiz-Perez et al., 2004 (16).
[2]Product contains sufficient histidine to support *S.* Typhimurium histidine auxotroph SL1344.

TABLE S4. MG-RAST chicken cecal transcriptome in response to *Salmonella* abundance

MG-RAST ID[1]	ID	Sample[2]	bp count	seq. count	Material	Library	Method[3]	*Salmonella* Abundance (Log10 CFU/g)[4]
mgm4692833.3	mgs465805	21d1	1.89E+09	1.68E+07	cecal contents	mgl465807	Illumina	8.51
mgm4692954.3	mgs466021	21d2	1.95E+09	1.75E+07	cecal contents	mgl466023	Illumina	7.00
mgm4693685.3	mgs467371	21d3	2.54E+09	2.19E+07	cecal contents	mgl467373	Illumina	9.14
mgm4693971.3	mgs468586	21d4	1.90E+09	1.65E+07	cecal contents	mgl468588	Illumina	9.22
mgm4694327.3	mgs469643	21d5	2.12E+09	1.84E+07	cecal contents	mgl469645	Illumina	7.00
mgm4694708.3	mgs471524	28d1	1.53E+09	1.32E+07	cecal contents	mgl471526	Illumina	5.85
mgm4694762.3	mgs471569	28d2	2.30E+09	2.00E+07	cecal contents	mgl471571	Illumina	7.20
mgm4694925.3	mgs471740	28d3	2.75E+09	2.36E+07	cecal contents	mgl471742	Illumina	5.45
mgm4698334.3	mgs478480	28d5	1.91E+09	1.70E+07	cecal contents	mgl478482	Illumina	7.60
mgm4698338.3	mgs478483	35d1	8.74E+08	7.70E+06	cecal contents	mgl478485	Illumina	6.89
mgm4696219.3	mgs473851	35d2	1.78E+09	1.56E+07	cecal contents	mgl473853	Illumina	6.79
mgm4696521.3	mgs474005	35d3	1.59E+09	1.42E+07	cecal contents	mgl474007	Illumina	0.00
mgm4696883.3	mgs475929	35d4	7.95E+08	6.90E+06	cecal contents	mgl475931	Illumina	7.86
mgm4697357.3	mgs476535	35d5	3.05E+09	2.71E+07	cecal contents	mgl476537	Illumina	5.78
mgm4697717.3	mgs477158	42d1	3.32E+09	3.01E+07	cecal contents	mgl477160	Illumina	0.00
mgm4697857.3	mgs477286	42d2	1.73E+09	1.59E+07	cecal contents	mgl477288	Illumina	2.11
mgm4698147.3	mgs477391	42d3	1.98E+09	1.73E+07	cecal contents	mgl477393	Illumina	3.64
mgm4698298.3	mgs477937	42d4	9.14E+08	8.12E+06	cecal contents	mgl477939	Illumina	5.30

[1]Access to chicken cecal transcriptomes in MG-RAST (17): https://www.mg-rast.org/linkin.cgi?metagenome= followed by MG-RAST ID. For example: https://www.mg-rast.org/linkin.cgi?metagenome=mgm4692833.3, with there being no space between "=" and mgm4692833. [2]Sample ID is as follows xxd designates day of age; for example, 21d is chicken at 21 days of age, and xxdy designates subject. 21d1 is chicken subject 1 at 21 days of age. 3Sequences were generated by Illumina RNA seq. 4Salmonella abundance was determined for nucleic acid extracted from pelleted bacteria present in cecal contents by qPCR (18).

Table S5. List of enzymes associated with fermentation in KO data det from MG-RAST.

glycerol dehydratase, propanediol dehydratase, propionate kinase, propionyl-CoA carboxylase, methylmalonyl-CoA mutase, propionaldehyde dehydrogenase, propionate-CoA transferase, propionyl-CoA synthetase, propanediol utilization protein, butyrate kinase, glutaconate-CoA transferase, butyryl-CoA dehydrogenase, pyruvate dehydrogenase, pyruvate oxidase, acetate kinase, acetyl-CoA hydrolase, pyruvate ferredoxin oxidoreductase, acetyl-CoA synthetase, formate C-acetyltransferase, serine dehydratase, ferredoxin hydrogenase, lactate dehydrogenase, 4-aminobutyrate aminotransferase, ethanolamine utilization protein, ethanolamine ammonia lyase, vitamin B12, aldehyde dehydrogenase, and lactaldehyde reductase.

Table S6. List of catabolic enzymes in KO data set from MG-RAST.

Fermentation: ethanolamine ammonia lyase, ethanolamine utilization protein, glycerol dehydratase, 1,2-propanediol dehydratase, propanediol utilization protein, propionate kinase, propionyl CoA carboxylase, propionate CoA transferase, propionyl CoA synthetase, propionaldehyde dehydrogenase, acetyl propionyl CoA carboxylase, Na-transporting methylmalonyl CoA oxaloacetate decarboxylase, oxaloacetate decarboxylase, methylmalonyl CoA mutase, acetyl propionyl CoA carboxylase, cobalamin, cobalamin biosynthesis, vitamin B12 transport system permease protein, glutamate decarboxylase, 4-aminobutyrate aminotransferase, γ-aminobutyrate permease, serine dehydratase, tryptophanase, cysteine desulfhydrase , serine decarboxylase , phosphatidylserine decarboxylase , glycerol dehydrogenase, dihydroxyacetone kinas, glycerol kinase, glycerol 3 phosphate transport system, glycerol 3 phosphate dehydrogenase, glyceraldehyde 3 phosphate dehydrogenase, acyl CoA dehydrogenase, enoyl CoA hydratase, hydroxyacyl CoA dehydrogenase, butyrate kinase, butyryl CoA dehydrogenase, butyryl CoA acetate CoA transferase, hydroxybutyrate CoA transferase, acyl CoA acetate 3 ketoacid CoA transferase, acetyl CoA acetyltransferase, glutaconate CoA transferase, pyruvate formate lyase, pyruvate dehydrogenase, pyruvate oxidase, acetate CoA transferase, acetyl CoA synthase, acetyl CoA hydrolase, acetate kinase, lactate dehydrogenase, pyruvate ferredoxin oxidoreductase, pyruvate synthase, formate C-acetyltransferase, formate hydrogenlyase, ferredoxin hydrogenase.

Carbohydrate Metabolism: glycogen, starch, amylase, pyruvate carboxylase, phosphoenolpyruvate carboxykinase, fructose -1-6-bisphosphatase, phosphofructokinase, glucose-6 phosphate isomerase, phosphoglucoisomerase, glucose 6 phosphatase, phosphotransferase system, PTS, ABC type sugar transport system, ABC type glucose galactose transport system , methyl galactoside transport system permease protein, phosphoglycerate kinase, pyruvate kinase, sorbosone dehydrogenase, glucose sorbosone dehydrogenases, glucose dehydrogenase, glucose oxidase, beta fructosidase, α-galactosidase, β-galactosidase, α-glucosidase, β-glucosidase, α-mannosidase, α-glucuronidase, β-glucuronidase, β-xylosidase, xylanase, β-fructofuranosidase, β-N-acetylhexosaminidase, β-hexosaminidase, cellulase, glycosidase, glycosyl hydrolases, endoglucanase, fructuronate, mannonate , fructoselysine, chitinase, Dihydroxyacid dehydratase phosphogluconate dehydratase, 2-keto-3-deoxy-6-phosphogluconate aldolase 2-dehydro-3-deoxyphosphogluconate aldolase, fructose-6-phosphate phosphoketolase, 6-phosphogluconolactonase, phosphogluconate dehydrogenase, α-L-fucosidase, fucose permease, L-fucose isomerase and related proteins, L-fucose isomerase ,

fucose dissimilation pathway protein FucU, L-fucose mutarotase, L-fuculokinase, L-fuculose phosphate aldolase, rhamnosidase, L-rhamnose isomerase, rhamnulokinase, lactaldehyde reductase, sialidase, N-acetylneuraminate lyase, arabinose efflux permease, L-arabinose transport system permease protein, L arabinose isomerase, α-N arabinofuranosidase, sorbitol dehydrogenase, sorbitol 6-phosphate-2-dehydrogenase, adenylate cyclase.

Peptide and amino acid metabolism: oligopeptide, peptide nickel transport system permease protein, GTP pyrophosphokinase, phosphoenolpyruvate carboxylase, urease, glutamate dehydrogenase, ornithine acetylornithine aminotransferase , ornithine carbamoyltransferase , carbamoylphosphate synthase, argininosuccinate lyase, argininosuccinate synthase, arginase agmatinase, acetylornithine aminotransferase, acetylornithine deacetylase, arginine deiminase, carbamate kinase, arginine lysine ornithine decarboxylases, arginine ornithine N-succinyltransferase, ornithine N-succinyltransferase, arginine kinase.

Respiration: ubiquinone oxidoreductase, cytochrome o ubiquinol oxidase, cytochrome bd type quinol oxidase, cytochrome bd I oxidase, ubiquinol cytochrome c reductase, trimethylamine N-oxide reductase, heme copper type cytochrome quinol oxidase, thiosulfate reductase, nitrate reductase, nitrite reductase, nitric oxide reductase, formate dehydrogenase.

Miscellaneous: carbonic anhydrase, superoxide dismutase, catalase, polyketide, non-ribosomal peptide synthetase, type III secretion, type IV secretion.

Table S7. List of enzymes and proteins associated with stress response in stress and virulence data sets in MG-RAST.

Heat Shock: DnaK, DnaJ, GrpE, RpoH, HrcA, hypothetical radical SAM family enzyme in heat shock, HtrA, Yci, RdgB, YggW, ribosome-associated heat shock protein, serine protease).

Carbon Starvation: carbon starvation protein A, RspA, RspB, starvation lipoprotein Slp paralog, starvation lipoprotein Slp, carbon storage regulator, YihV, YihR, YihS, cellobiose phosphorylase, YshA, lactoylglutathione lyase, SgrR, YihW, various polyols ABC transporters, universal stress protein A-G, universal stress protein family, stringent starvation protein B, YciT, CspA-G, CspI).

Extra-cytoplasmic/Envelope Stress Response: phage shock protein A, C, E, Psp operon transcriptional activator, RseP, Hfq, outer membrane protein A, NmpC, Deg, outer membrane protein H precursor, RseA,B, DedA, phosphatidylglycerophosphatase.

Regulation: RNA polymerase sigma factor, SigB, transcriptional regulator, two-component sensor histidine kinase, RsbT, RsbV, RasP/Ylu, Rsb, YkgA.

Translation and Protein Export: ribosomal RNA small subunit methyltransferase E, rRNA small subunit methyltransferase I, ribosomal protein L11 methyltransferase, NAD-dependent protein deacetylase of SIR2 family, signal peptidase-like protein, LepA, HflX, GTP-binding protein related to HflX, HflC, YjeT, HflK, D-tyrosyl-tRNA(Tyr) deacylase, ribonuclease PH, SurA, MiaB, SsrA-binding protein SmpB, tmRNA-binding protein SmpB, protein arginine N-methyltransferas .

Oxidative Stress: hypothetical radical SAM family enzyme in heat shock, NAD-dependent glyceraldehyde-3-phosphate dehydrogenase, rubredoxin, rubredoxin-NAD(+) reductase, rubredoxin-oxygen oxidoreductase, rubrerythrin, ArcA,B, QorR, superoxide reductase, redox-sensitive transcriptional regulator, glutathione peroxidase, glutathione S-transferase, uncharacterized glutathione S-transferase-like protein, glutaredoxin, Nrd, glutaredoxin 1, glutaredoxin 2, GrlA, Sarcosine oxidase, glycine N-methyltransferase, alkyl hydroperoxide reductase, OT coproporphyrinogen III oxidase, γ-glutamyltranspeptidase, peroxide stress regulator, radical SAM family enzymes, glutamate--cysteine ligase, paraquat-inducible protein A,B, glutathionylspermidine synthase, glutathionylspermidine amidohydrolase, peroxidase, glutathione reductase, glutathione peroxidase, CoA-disulfide reductase, cytochrome c551 peroxidase, Hydroxyacylglutathione hydrolase, radical SAM protein 2, catalase, ferroxidase, iron-binding ferritin-like antioxidant protein, HemW, yghU, yibF yncG, putative bacterial , haemoglobin, glutaredoxin-related protein, flavohemoprotein, YihU, 3-phenylpropionate dioxygenase, dimethylarginine dimethylaminohydrolase, GshF, hydrogen peroxide-inducible genes activator, S-(hydroxymethyl)glutathione dehydrogenase, glutathione synthetase, predicted alternative glutathione synthetase, S-formylglutathione hydrolase, radical SAM family heme chaperone, NADPH: quinone oxidoreductase 2, Nicotinate phosphoribosyltransferase, Nicotinamidase, Xanthosine/inosine triphosphate pyrophosphatase, Xanthosine/inosine triphosphate diphosphatase, betaine aldehyde dehydrogenase, fumarate and nitrate reduction regulatory protein, diguanylate cyclase/phosphodiesterase, probable peroxiredoxin, organic hydroperoxide resistance protein, organic hydroperoxide resistance transcriptional regulator, thiol: disulfide oxidoreductase, poly [ADP-ribose] polymerase-1, SoxS, FrmR, 5-oxoprolinase.

Osmotic Stress: BetI, BetT, aquaporin Z, choline ABC transport system, glycine betaine ABC transport system, L-proline glycine betaine ABC transport system permease, YehW, YehX-Z, choline binding protein A, choline-sulfatase, choline dehydrogenase, osmotically activated L-carnitine/choline ABC transporter, OsmY, glycerol uptake facilitator protein, glucans biosynthesis protein C, D, G, H, NdvA, phosphoglycerol transferase I.

Acid Tolerance: arginine decarboxylase, arginine/agmatine antiporter, ornithine aminotransferase, probable glutamate/γ-aminobutyrate antiporter, glutamate decarboxylase, GadE, glutamate transport ATP-binding protein, glutamate transport permease protein, glutamate transport substrate-binding protein, glutamate transport membrane-spanning protein, YbaT, putative membrane transporter ATPase, HdeA, B.

Iron Metabolism: ferric uptake regulation protein, iron chelate uptake ABC transporter family permease, ABC-type Fe3+-siderophore transport system).

Antimicrobials: YihO, Yih, TehA,B, putative transferase clustered with tellurite resistance proteins TehA/TehB, TsgA, ZUR, MarA, bacteriocin.

Miscellaneous: Diaminobutyrate-pyruvate aminotransferase, CysA, polysulfide binding and transferase domain, AraC family transcriptional regulator, Phosphoesterase, YihT, YihQ, YabA, PduF (1,2-propanediol diffusion facilitator).

Polyketide Synthesis: regulator of polyketide synthase expression, polyketide synthase module, non-ribosomal peptide synthetase module, acyl-CoA synthetases (AMP-forming)/AMP-acid ligases II, aryl carrier domain, thioesterase domains of type I polyketide synthases, thioesterase involved in non-ribosomal peptide biosynthesis, O-methyltransferase involved in polyketide biosynthesis,

glutamate-1-semialdehyde aminotransferase, putative dehydrogenase domain of multifunctional non-ribosomal peptide synthetases and related enzymes, yersiniabactin non-ribosomal peptide synthetase, yersiniabactin non-ribosomal peptide/polyketide synthase, yersiniabactin synthetase, yersiniabactin salicyl-AMP ligase, mycobactin phenyloxazoline synthetase, mycobactin salicyl-AMP ligase.

REFERENCES

1. **Herrero M, de Lorenzo V, Timmis KN.** 1990. Transposon vectors containing non-antibiotic resistance selection markers for cloning and stable chromosomal insertion of foreign genes in gram-negative bacteria. J Bacteriol **172:**6557-6567.
2. **Miller VL, Mekalanos JJ.** 1988. A novel suicide vector and its use in construction of insertion mutations: osmoregulation of outer membrane proteins and virulence determinants in Vibrio cholerae requires toxR. J Bacteriol **170:**2575-2583.
3. **Sanderson KE, Roth JR.** 1988. Linkage map of Salmonella typhimurium, edition VII. Microbiol Rev **52:**485-532.
4. **Cheng Y, Pedroso AA, Porwollik S, McClelland M, Lee MD, Kwan T, Zamperini K, Soni V, Sellers HS, Russell SM, Maurer JJ.** 2015. rpoS-Regulated core genes involved in the competitive fitness of Salmonella enterica Serovar Kentucky in the intestines of chickens. Appl Environ Microbiol **81:**502-514.
5. **Brinsmade SR, Paldon T, Escalante-Semerena JC.** 2005. Minimal functions and physiological conditions required for growth of salmonella enterica on ethanolamine in the absence of the metabolosome. J Bacteriol **187:**8039-8046.
6. **Smith BP, Reina-Guerra M, Hoiseth SK, Stocker BA, Habasha F, Johnson E, Merritt F.** 1984. Aromatic-dependent Salmonella typhimurium as modified live vaccines for calves. Am J Vet Res **45:**59-66.
7. **Provence DL, and R. Curtiss III.** 1994. Gene transfer in Gram-negative bacteria. ASM Press, Washington, D.C.
8. **Miller WG, Leveau JH, Lindow SE.** 2000. Improved gfp and inaZ broad-host-range promoter-probe vectors. Mol Plant Microbe Interact **13:**1243-1250.
9. **Miyashiro T, Goulian M.** 2007. Stimulus-dependent differential regulation in the Escherichia coli PhoQ PhoP system. Proc Natl Acad Sci U S A **104:**16305-16310.
10. **Datsenko KA, Wanner BL.** 2000. One-step inactivation of chromosomal genes in Escherichia coli K-12 using PCR products. Proc Natl Acad Sci U S A **97:**6640-6645.
11. **Cherepanov PP, Wackernagel W.** 1995. Gene disruption in Escherichia coli: TcR and KmR cassettes with the option of Flp-catalyzed excision of the antibiotic-resistance determinant. Gene **158:**9-14.
12. **Drepper T, Eggert T, Circolone F, Heck A, Krauss U, Guterl JK, Wendorff M, Losi A, Gartner W, Jaeger KE.** 2007. Reporter proteins for in vivo fluorescence without oxygen. Nat Biotechnol **25:**443-445.
13. **Dower WJ, Miller JF, Ragsdale CW.** 1988. High efficiency transformation of E. coli by high voltage electroporation. Nucleic Acids Res **16:**6127-6145.
14. **Bochner BR, Huang HC, Schieven GL, Ames BN.** 1980. Positive selection for loss of tetracycline resistance. J Bacteriol **143:**926-933.
15. **Suzuki MT, Taylor LT, DeLong EF.** 2000. Quantitative analysis of small-subunit rRNA genes in mixed microbial populations via 5'-nuclease assays. Applied and environmental microbiology **66:**4605-4614.
16. **Ruiz-Perez F, Sheikh J, Davis S, Boedeker EC, Nataro JP.** 2004. Use of a continuous-flow anaerobic culture to characterize enteric virulence gene expression. Infect Immun **72:**3793-3802.
17. **Keegan KP, Glass EM, Meyer F.** 2016. MG-RAST, a Metagenomics Service for Analysis of Microbial Community Structure and Function. Methods Mol Biol **1399:**207-233.
18. **Pedroso AA, Lee MD, Maurer JJ.** 2021. Strength Lies in Diversity: How Community Diversity Limits Salmonella Abundance in the Chicken Intestine. Frontiers in Microbiology **12**.
