# Supplementary figures and images for: Peeling back the many layers of competitive exclusion
Source: Front Microbiol. 2024 Mar 21;15:1342887. doi: 10.3389/fmicb.2024.1342887 (PMC11000858; doi:10.3389/fmicb.2024.1342887)

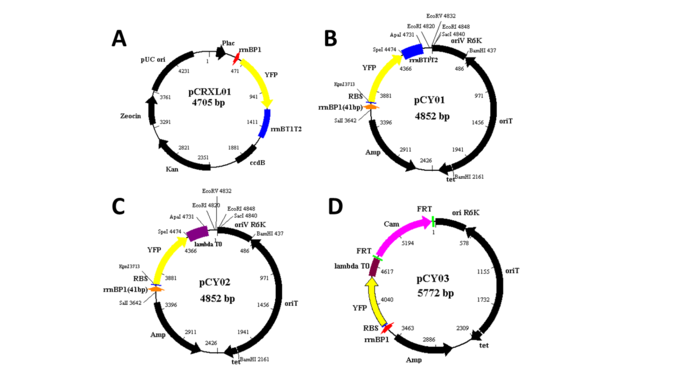

Supplement: Supplementary file 2 [file Image_1.TIFF]

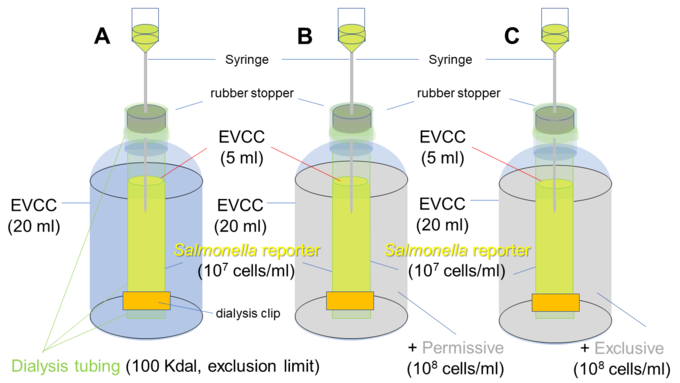

Supplement: Supplementary file 3 [file Image_2.TIFF]

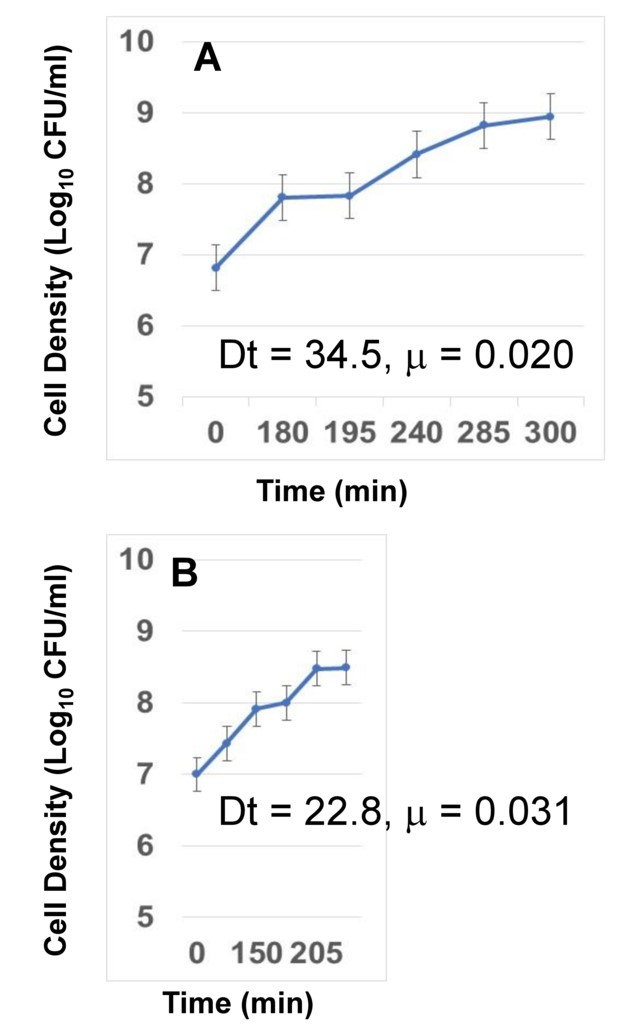

Supplement: Supplementary file 4 [file Image_3.TIFF]

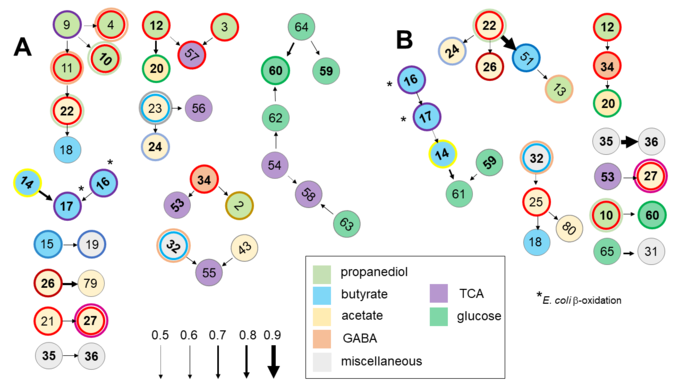

Supplement: Supplementary file 5 [file Image_4.TIFF]
