# Supplementary material for: Peeling back the many layers of competitive exclusion
Source: Front Microbiol. 2024 Mar 21;15:1342887. doi: 10.3389/fmicb.2024.1342887 (PMC11000858; doi:10.3389/fmicb.2024.1342887)
Supplement: Supplementary file 8 [file Data_Sheet_2.docx]

**TABLE S8.** Identity of microbial community transcripts identified in Bayesian network analysis of cecal transcriptomes or correlated with *Salmonella* abundance in the chicken cecum

|  | **Universal Stress** | | | **Cold Shock** | | | | **Envelop Stress** | | | | | | | | | | | | | | **Oxidative Stress** | | | | | | |
| --- | --- | --- | --- | --- | --- | --- | --- | --- | --- | --- | --- | --- | --- | --- | --- | --- | --- | --- | --- | --- | --- | --- | --- | --- | --- | --- | --- | --- |
| **Genus/Species**^1^ | *uspD* | *uspE* | | *cspA* | *cspD* | *cspI* | | *pspA* | | *pspC* | *rseA* | *rseP* | | *ompA* | | *nmpC* | | | *deqQ* | | | *yihU* | | | *nrd* | | *yncG* | *gapA* |
| *Escherichia coli* |  |  | |  |  |  | |  | |  |  |  | |  | |  | | |  | | |  | | |  | |  |  |
| *Salmonella enterica* |  |  | |  |  |  | |  | |  |  |  | |  | |  | | |  | | |  | | |  | |  |  |
| *Klebsiella pneumoniae* |  |  | |  |  |  | |  | |  |  |  | |  | |  | | |  | | |  | | |  | |  |  |
| *Enterococcus faecium* |  |  | |  |  |  | |  | |  |  |  | |  | |  | | |  | | |  | | |  | |  |  |
| *Enterococcus gallinarum* |  |  | |  |  |  | |  | |  |  |  | |  | |  | | |  | | |  | | |  | |  |  |
| *Enterococcus hirae* |  |  | |  |  |  | |  | |  |  |  | |  | |  | | |  | | |  | | |  | |  |  |
| *Anaerostipes caccae* |  |  | |  |  |  | |  | |  |  |  | |  | |  | | |  | | |  | | |  | |  |  |
| *Clostridioides difficile* |  |  | |  |  |  | |  | |  |  |  | |  | |  | | |  | | |  | | |  | |  |  |
| *Clostridium sp. C1*^7^ |  |  | |  |  |  | |  | |  |  |  | |  | |  | | |  | | |  | | |  | |  |  |
| *Clostridium sp. M62/1*^7^ |  |  | |  |  |  | |  | |  |  |  | |  | |  | | |  | | |  | | |  | |  |  |
| *Flavonifractor plautii* |  |  | |  |  |  | |  | |  |  |  | |  | |  | | |  | | |  | | |  | |  |  |
| *Oscillibacter sp. PEA192*^7^ |  |  | |  |  |  | |  | |  |  |  | |  | |  | | |  | | |  | | |  | |  |  |
|  | **Oxidative Stress** | | | | | | | | | | | | | | | | | | | | | | | | | | | |
| **Genus/Species**^1^ | *gstA* | *gstB* | | *gss* | *cdr* | *katG* | | *dps* | | *gshA* | *gshB* | *gshF* | | *ggt* | | *grxC* | | | *qorR* | | | *hmpA* | | | *ohr* | | *gloB* | *α* |
| *Escherichia coli* |  |  | |  |  |  | |  | |  |  |  | |  | |  | | |  | | |  | | |  | |  |  |
| *Salmonella enterica* |  |  | |  |  |  | |  | |  |  |  | |  | |  | | |  | | |  | | |  | |  |  |
| *Enterococcus durans* |  |  | |  |  |  | |  | |  |  |  | |  | |  | | |  | | |  | | |  | |  |  |
| *Enterococcus faecium* |  |  | |  |  |  | |  | |  |  |  | |  | |  | | |  | | |  | | |  | |  |  |
| *Enterococcus gallinarum* |  |  | |  |  |  | |  | |  |  |  | |  | |  | | |  | | |  | | |  | |  |  |
| *Clostridium sp. C1*^7^ |  |  | |  |  |  | |  | |  |  |  | |  | |  | | |  | | |  | | |  | |  |  |
| *Clostridium AM29-11AC*^7^ |  |  | |  |  |  | |  | |  |  |  | |  | |  | | |  | | |  | | |  | |  |  |
| *Flavonifractor plautii* |  |  | |  |  |  | |  | |  |  |  | |  | |  | | |  | | |  | | |  | |  |  |
| *Oscillibacter sp. PEA192*^7^ |  |  | |  |  |  | |  | |  |  |  | |  | |  | | |  | | |  | | |  | |  |  |
|  | **Oxidative Stress** | | | |  |  | |  | |  |  |  | |  | |  | | |  | | |  | | |  | |  |  |
| **Genus/Species**^1^ | *β* | *cysA* | | *γ* |  |  | |  | |  |  |  | |  | |  | | |  | | |  | | |  | |  |  |
| *Escherichia coli* |  |  | |  |  |  | |  | |  |  |  | |  | |  | | |  | | |  | | |  | |  |  |
| *Clostridiales CCNA10*^7^ |  |  | |  |  |  | |  | |  |  |  | |  | |  | | |  | | |  | | |  | |  |  |
| *Clostridiales sp. SM4/1*^7^ |  |  | |  |  |  | |  | |  |  |  | |  | |  | | |  | | |  | | |  | |  |  |
| *Anaerostipes caccae* |  |  | |  |  |  | |  | |  |  |  | |  | |  | | |  | | |  | | |  | |  |  |
| *Clostridioides difficile* |  |  | |  |  |  | |  | |  |  |  | |  | |  | | |  | | |  | | |  | |  |  |
| *Clostridium innocuum* |  |  | |  |  |  | |  | |  |  |  | |  | |  | | |  | | |  | | |  | |  |  |
| *Clostridium perfringens* |  |  | |  |  |  | |  | |  |  |  | |  | |  | | |  | | |  | | |  | |  |  |
| *Clostridium sp. C1*^7^ |  |  | |  |  |  | |  | |  |  |  | |  | |  | | |  | | |  | | |  | |  |  |
| *Clostridium sp. K10*^7^ |  |  | |  |  |  | |  | |  |  |  | |  | |  | | |  | | |  | | |  | |  |  |
| *Clostridium sp. M62/1*^7^ |  |  | |  |  |  | |  | |  |  |  | |  | |  | | |  | | |  | | |  | |  |  |
| *Flavonifractor plautii* |  |  | |  |  |  | |  | |  |  |  | |  | |  | | |  | | |  | | |  | |  |  |
| *Oscillibacter sp. PEA192*^7^ |  |  | |  |  |  | |  | |  |  |  | |  | |  | | |  | | |  | | |  | |  |  |
| *Romboutsia hominis* |  |  | |  |  |  | |  | |  |  |  | |  | |  | | |  | | |  | | |  | |  |  |
| *Romboutsia ilealis* |  |  | |  |  |  | |  | |  |  |  | |  | |  | | |  | | |  | | |  | |  |  |
|  | **Heat Shock** | | | | | | | | **Starvation** | | | | | | | | | | | | | | **Antimicrobials** | | | | | |
| **Genus/Species**^1^ | *dnaJ* | *dnaK* | | *hrcA* | *htrA* | | *yci* | | *cstA* | *sgrR* | *yihS* | | *rspA* | | *slp* | | | *yihW* | | *pqiA* | | | *lepA* | | | *rsmI* | *rsmE* | *ykgA* |
| *Escherichia coli* |  |  | |  |  | |  | |  |  |  | |  | |  | | |  | |  | | |  | | |  |  |  |
| *Salmonella enterica* |  |  | |  |  | |  | |  |  |  | |  | |  | | |  | |  | | |  | | |  |  |  |
| *Enterococcus faecium* |  |  | |  |  | |  | |  |  |  | |  | |  | | |  | |  | | |  | | |  |  |  |
| *Enterococcus gallinarum* |  |  | |  |  | |  | |  |  |  | |  | |  | | |  | |  | | |  | | |  |  |  |
| *Clostridiales CCNA10*^7^ |  |  | |  |  | |  | |  |  |  | |  | |  | | |  | |  | | |  | | |  |  |  |
| *A. colihominis* |  |  | |  |  | |  | |  |  |  | |  | |  | | |  | |  | | |  | | |  |  |  |
| *Blautia argi* |  |  | |  |  | |  | |  |  |  | |  | |  | | |  | |  | | |  | | |  |  |  |
| *Clostridium sp. C1*^7^ |  |  | |  |  | |  | |  |  |  | |  | |  | | |  | |  | | |  | | |  |  |  |
| *Clostridium sp. K10*^7^ |  |  | |  |  | |  | |  |  |  | |  | |  | | |  | |  | | |  | | |  |  |  |
| *Clostridium sp. M62/1*^7^ |  |  | |  |  | |  | |  |  |  | |  | |  | | |  | |  | | |  | | |  |  |  |
| *Clostridium innocuum* |  |  | |  |  | |  | |  |  |  | |  | |  | | |  | |  | | |  | | |  |  |  |
| *Clostridium perfringens* |  |  | |  |  | |  | |  |  |  | |  | |  | | |  | |  | | |  | | |  |  |  |
| *Dysosmobacter Q4140*^7^ |  |  | |  |  | |  | |  |  |  | |  | |  | | |  | |  | | |  | | |  |  |  |
| *E. ramosum* |  |  | |  |  | |  | |  |  |  | |  | |  | | |  | |  | | |  | | |  |  |  |
| *Flavonifractor plautii* |  |  | |  |  | |  | |  |  |  | |  | |  | | |  | |  | | |  | | |  |  |  |
| *Flintibacter KGMB00164*^7^ |  |  | |  |  | |  | |  |  |  | |  | |  | | |  | |  | | |  | | |  |  |  |
| *L. phocaeense* |  |  | |  |  | |  | |  |  |  | |  | |  | | |  | |  | | |  | | |  |  |  |
| *Oscillibacter sp. PEA192*^7^ |  |  | |  |  | |  | |  |  |  | |  | |  | | |  | |  | | |  | | |  |  |  |
| *Romboutsia sp. 13368*^7^ |  |  | |  |  | |  | |  |  |  | |  | |  | | |  | |  | | |  | | |  |  |  |
| *Sellimonas intestinalis* |  |  | |  |  | |  | |  |  |  | |  | |  | | |  | |  | | |  | | |  |  |  |
|  | **Antimicrobials** | | | | | | | | | | | | | | | | | | | | | | | | | | | |
| **Genus/Species**^1^ | *dedA* | *frmR* | | *tehA* | *tehB* | | *ybaT* | | *yibF* | *aadA1* | *spw* | | *ant(9)* | | *erm(B)* | | | *ermG* | | *cbrC* | | | *yejB* | | | *creA* | *creB* | *creC* |
| *Escherichia coli* |  |  | |  |  | |  | |  | ^4^ |  | |  | | ^4^ | | |  | |  | | |  | | |  |  |  |
| *Salmonella enterica* |  |  | |  |  | |  | |  | ^4^ |  | |  | | ^4^ | | |  | |  | | |  | | |  |  |  |
| *Campylobacter jejuni* |  |  | |  |  | |  | |  |  |  | | ^4^ | |  | | |  | |  | | |  | | |  |  |  |
| *B. thetaiotaomicron* |  |  | |  |  | |  | |  |  |  | | ^4^ | |  | | | ^4^ | |  | | |  | | |  |  |  |
| *Enterococcus faecalis* |  |  | |  |  | |  | |  |  | ^4^ | |  | | ^4^ | | | ^4^ | |  | | |  | | |  |  |  |
| *Enterococcus faecium* |  |  | |  |  | |  | |  |  | ^4^ | |  | | ^4^ | | | ^4^ | |  | | |  | | |  |  |  |
| *Enterococcus gallinarum* |  |  | |  |  | |  | |  |  | ^4^ | |  | | ^4^ | | |  | |  | | |  | | |  |  |  |
| *Lactobacillus crispatus* |  |  | |  |  | |  | |  |  |  | | ^4^ | |  | | |  | |  | | |  | | |  |  |  |
| *Clostridioides difficile* |  |  | |  |  | |  | |  |  | ^4^ | | ^4^ | |  | | | ^4^ | |  | | |  | | |  |  |  |
| *Clostridium sp. C1*^7^ |  |  | |  |  | |  | |  |  |  | | ^4^ | |  | | |  | |  | | |  | | |  |  |  |
| *Clostridium sp. M62/1*^7^ |  |  | |  |  | |  | |  |  |  | |  | |  | | |  | |  | | |  | | |  |  |  |
| *L. phocaeense* |  |  | |  |  | |  | |  |  |  | |  | |  | | |  | |  | | |  | | |  |  |  |
| *Flavonifractor plautii* |  |  | |  |  | |  | |  |  |  | |  | |  | | |  | |  | | |  | | |  |  |  |
| *Flintibacter KGMB00164*^7^ |  |  | |  |  | |  | |  |  |  | |  | |  | | |  | |  | | |  | | |  |  |  |
| *Oscillibacter sp. PEA192*^7^ |  |  | |  |  | |  | |  |  |  | |  | |  | | |  | |  | | |  | | |  |  |  |
| *Sellimonas intestinalis* |  |  | |  |  | |  | |  |  |  | |  | |  | | |  | |  | | |  | | |  |  |  |
|  | **Antimicrobials** | | | | | | | | **Osmotic Stress** | | | | | | | | |  | |  | | | **Carbohydrate Metabolism** | | | | | |
| **Genus/Species**^1^ | *creD* | *tolA* | | *irp1* | *δ* | | *ε* | | *proV* | *opgB* | *yciT* | | *betI* | | *yehZ* | | | *lsr* | | *luxS* | | | *ζ* | | | *araJ* | *η* |  |
| *Escherichia coli* |  |  | | ^5^ |  | |  | |  |  |  | |  | |  | | |  | |  | | |  | | |  | ^6^ |  |
| *Salmonella enterica* |  |  | |  |  | |  | |  |  |  | |  | |  | | |  | |  | | |  | | |  |  |  |
| *Klebsiella pneumoniae* |  |  | | ^5^ |  | |  | |  |  |  | |  | |  | | |  | |  | | |  | | |  |  |  |
| *Enterococcus faecium* |  |  | |  |  | |  | |  |  |  | |  | |  | | |  | |  | | |  | | |  |  |  |
| *Enterococcus gallinarum* |  |  | |  |  | |  | |  |  |  | |  | |  | | |  | |  | | |  | | |  |  |  |
| *L. vaginalis* |  |  | |  |  | |  | |  |  |  | |  | |  | | |  | |  | | |  | | |  |  |  |
| *Clostridiales sp. SM4/1*^7^ |  |  | |  |  | |  | |  |  |  | |  | |  | | |  | |  | | |  | | |  |  |  |
| *A. colihominis* |  |  | |  |  | |  | |  |  |  | |  | |  | | |  | |  | | |  | | |  |  |  |
| *Clostridium perfringens* |  |  | |  |  | |  | |  |  |  | |  | |  | | |  | |  | | |  | | |  |  |  |
| *Clostridium sp. C1*^7^ |  |  | |  |  | |  | |  |  |  | |  | |  | | |  | |  | | |  | | |  |  |  |
| *Clostridium sp. K10*^7^ |  |  | |  |  | |  | |  |  |  | |  | |  | | |  | |  | | |  | | |  |  |  |
| *D. welbionis* |  |  | |  |  | |  | |  |  |  | |  | |  | | |  | |  | | |  | | |  |  |  |
| *Flavonifractor plautii* |  |  | |  |  | |  | |  |  |  | |  | |  | | |  | |  | | |  | | |  |  |  |
| *L. phocaeense* |  |  | |  |  | |  | |  |  |  | |  | |  | | |  | |  | | |  | | |  |  |  |
| *Oscillibacter sp. NSJ-62*^7^ |  |  | |  |  | |  | |  |  |  | |  | |  | | |  | |  | | |  | | |  |  |  |
| *Oscillibacter sp. PEA192*^7^ |  |  | |  |  | |  | |  |  |  | |  | |  | | |  | |  | | |  | | |  |  |  |
| *Sellimonas intestinalis* |  |  | |  |  | |  | |  |  |  | |  | |  | | |  | |  | | |  | | |  |  |  |
| *Subdoligranulum variabile* |  |  | |  |  | |  | |  |  |  | |  | |  | | |  | |  | | |  | | |  |  |  |
| *Turicibacter sp. TJ11*^7^ |  |  | |  |  | |  | |  |  |  | |  | |  | | |  | |  | | |  | | |  |  |  |
|  |  |  | | **Fucose** | | | | | **Propanediol/Propionate** | | | | | | | | | | | | | | |  | |  |  |  |
| **Genus/Species**^1^ | *nagB* | *lamB* | | *fucA* | *fucI* | | *fucK* | | *pduC* | *pduD* | *pduF* | | *pduL* | | *pduP* | | | *pduW* | | | *prpE* | | | *pcc* | | *pct* | *eutA* | *eutB* |
| *Escherichia coli* |  |  | |  |  | |  | |  |  |  | |  | |  | | |  | | |  | | |  | |  |  |  |
| *Salmonella enterica* |  |  | |  |  | |  | |  |  |  | |  | |  | | |  | | |  | | |  | |  |  |  |
| *Enterococcus avium* |  |  | |  |  | |  | |  |  |  | |  | |  | | |  | | |  | | |  | |  |  |  |
| *Enterococcus faecium* |  |  | |  |  | |  | |  |  |  | |  | |  | | |  | | |  | | |  | |  |  |  |
| *Clostridiales 02340*^2,7^ |  |  | |  |  | |  | |  |  |  | |  | |  | | |  | | |  | | |  | |  |  |  |
| *Anaerostipes caccae* |  |  | |  |  | |  | |  |  |  | |  | |  | | |  | | |  | | |  | |  |  |  |
| *A. colihominis* |  |  | |  |  | |  | |  |  |  | |  | |  | | |  | | |  | | |  | |  |  |  |
| *Clostridioides difficile* |  |  | |  |  | |  | |  |  |  | |  | |  | | |  | | |  | | |  | |  |  |  |
| *Clostridium sp. C1*^7^ |  |  | |  |  | |  | |  |  |  | |  | |  | | |  | | |  | | |  | |  |  |  |
| *Clostridium sp. K10*^7^ |  |  | |  |  | |  | |  |  |  | |  | |  | | |  | | |  | | |  | |  |  |  |
| *Clostridium sp. M62/1*^7^ |  |  | |  |  | |  | |  |  |  | |  | |  | | |  | | |  | | |  | |  |  |  |
| *L. phocaeense* |  |  | |  |  | |  | |  |  |  | |  | |  | | |  | | |  | | |  | |  |  |  |
| *Flavonifractor plautii* |  |  | |  |  | |  | |  |  |  | |  | |  | | |  | | |  | | |  | |  |  |  |
| *Oscillibacter sp. PEA192*^7^ |  |  | |  |  | |  | |  |  |  | |  | |  | | |  | | |  | | |  | |  |  |  |
| **Genus/Species**^1^ | *eutE* |  | |  |  | |  | |  |  |  | |  | |  | | |  | | |  | | |  | |  |  |  |
| *Escherichia coli* |  |  | |  |  | |  | |  |  |  | |  | |  | | |  | | |  | | |  | |  |  |  |
| *Salmonella enterica* |  |  | |  |  | |  | |  |  |  | |  | |  | | |  | | |  | | |  | |  |  |  |
|  | **Respiration** | | | | | | | | | | | | | | | | | | | | | | | | | | | |
| **Genus/Species** | *appC* | *cyoB* | | *hybD* | *hydA^3^* | | *narC* | | *narG* | *narH* | *narI* | | *narJ* | | *narV* | | | *narW* | | | *narY* | | | *narZ* | | *napB* | *napC* | *poxB* |
| *Escherichia coli* |  |  | |  |  | |  | |  |  |  | |  | |  | | |  | | |  | | |  | |  |  |  |
| *Salmonella enterica* |  |  | |  |  | |  | |  |  |  | |  | |  | | |  | | |  | | |  | |  |  |  |
| *Enterococcus faecalis* |  |  | |  |  | |  | |  |  |  | |  | |  | | |  | | |  | | |  | |  |  |  |
| *Enterococcus faecium* |  |  | |  |  | |  | |  |  |  | |  | |  | | |  | | |  | | |  | |  |  |  |
| *Enterococcus gallinarum* |  |  | |  |  | |  | |  |  |  | |  | |  | | |  | | |  | | |  | |  |  |  |
| *L. plantarum* |  |  | |  |  | |  | |  |  |  | |  | |  | | |  | | |  | | |  | |  |  |  |
| *Lactobacillus crispatus* |  |  | |  |  | |  | |  |  |  | |  | |  | | |  | | |  | | |  | |  |  |  |
| *Clostridioides difficile* |  |  | |  |  | |  | |  |  |  | |  | |  | | |  | | |  | | |  | |  |  |  |
| *Clostridium sp. M62/1*^7^ |  |  | |  |  | |  | |  |  |  | |  | |  | | |  | | |  | | |  | |  |  |  |
| *Oscillibacter sp. PEA192*^7^ |  |  | |  |  | |  | |  |  |  | |  | |  | | |  | | |  | | |  | |  |  |  |
|  |  |  | | **Fermentation** | | | | | | | | | | | | | | | | | | | | | | | | |
| **Genus/Species**^1^ | *arcA* | *hemL* | | *ldh* | *lldP* | | *pfo* | | *pta* | *aceE* | *acs* | | *ackA* | | *adhE* | | *paaH* | | | | *scpA* | | | *bcd* | | *gct* | *crt* | *hbd* |
| *Escherichia coli* |  |  | |  |  | |  | |  |  |  | |  | |  | |  | | | |  | | |  | |  |  |  |
| *Salmonella enterica* |  |  | |  |  | |  | |  |  |  | |  | |  | |  | | | |  | | |  | |  |  |  |
| *Enterococcus faecalis* |  |  | |  |  | |  | |  |  |  | |  | |  | |  | | | |  | | |  | |  |  |  |
| *Enterococcus faecium* |  |  | |  |  | |  | |  |  |  | |  | |  | |  | | | |  | | |  | |  |  |  |
| *Enterococcus gallinarum* |  |  | |  |  | |  | |  |  |  | |  | |  | |  | | | |  | | |  | |  |  |  |
| *Lactobacillus crispatus* |  |  | |  |  | |  | |  |  |  | |  | |  | |  | | | |  | | |  | |  |  |  |
| *Lactococcus cremoris* |  |  | |  |  | |  | |  |  |  | |  | |  | |  | | | |  | | |  | |  |  |  |
| *L. vaginalis* |  |  | |  |  | |  | |  |  |  | |  | |  | |  | | | |  | | |  | |  |  |  |
| *Clostridiales sp. SM4/1*^7^ |  |  | |  |  | |  | |  |  |  | |  | |  | |  | | | |  | | |  | |  |  |  |
| *Anaerobutyricum hallii* |  |  | |  |  | |  | |  |  |  | |  | |  | |  | | | |  | | |  | |  |  |  |
| *Anaerostipes caccae* |  |  | |  |  | |  | |  |  |  | |  | |  | |  | | | |  | | |  | |  |  |  |
| *B. hydrogenotrophica* |  |  | |  |  | |  | |  |  |  | |  | |  | |  | | | |  | | |  | |  |  |  |
| *A. colihominis* |  |  | |  |  | |  | |  |  |  | |  | |  | |  | | | |  | | |  | |  |  |  |
| *A. rhamnosivorans* |  |  | |  |  | |  | |  |  |  | |  | |  | |  | | | |  | | |  | |  |  |  |
| *Clostridioides difficile* |  |  | |  |  | |  | |  |  |  | |  | |  | |  | | | |  | | |  | |  |  |  |
| *Clostridium innocuum* |  |  | |  |  | |  | |  |  |  | |  | |  | |  | | | |  | | |  | |  |  |  |
| *Clostridium perfringens* |  |  | |  |  | |  | |  |  |  | |  | |  | |  | | | |  | | |  | |  |  |  |
| *Clostridium sp. C1*^7^ |  |  | |  |  | |  | |  |  |  | |  | |  | |  | | | |  | | |  | |  |  |  |
| *Clostridium sp. K10*^7^ |  |  | |  |  | |  | |  |  |  | |  | |  | |  | | | |  | | |  | |  |  |  |
| *Clostridium sp. M62/1*^7^ |  |  | |  |  | |  | |  |  |  | |  | |  | |  | | | |  | | |  | |  |  |  |
| *Dysosmobacter Q4140*^7^ |  |  | |  |  | |  | |  |  |  | |  | |  | |  | | | |  | | |  | |  |  |  |
| *E. ramosum* |  |  | |  |  | |  | |  |  |  | |  | |  | |  | | | |  | | |  | |  |  |  |
| *Eubacterium sp. c-25*^7^ |  |  | |  |  | |  | |  |  |  | |  | |  | |  | | | |  | | |  | |  |  |  |
| *F. prausnitzii* |  |  | |  |  | |  | |  |  |  | |  | |  | |  | | | |  | | |  | |  |  |  |
| *Flavonifractor plautii* |  |  | |  |  | |  | |  |  |  | |  | |  | |  | | | |  | | |  | |  |  |  |
| *Flintibacter KGMB00164*^7^ |  |  | |  |  | |  | |  |  |  | |  | |  | |  | | | |  | | |  | |  |  |  |
| *I. butyriciproducens* |  |  | |  |  | |  | |  |  |  | |  | |  | |  | | | |  | | |  | |  |  |  |
| *L. asaccharolyticus* |  |  | |  |  | |  | |  |  |  | |  | |  | |  | | | |  | | |  | |  |  |  |
| *L. phocaeense* |  |  | |  |  | |  | |  |  |  | |  | |  | |  | | | |  | | |  | |  |  |  |
| *Oscillibacter sp. PEA192*^7^ |  |  | |  |  | |  | |  |  |  | |  | |  | |  | | | |  | | |  | |  |  |  |
| *Sellimonas intestinalis* |  |  | |  |  | |  | |  |  |  | |  | |  | |  | | | |  | | |  | |  |  |  |
| *Turicibacter sp. TJ11*^7^ |  |  | |  |  | |  | |  |  |  | |  | |  | |  | | | |  | | |  | |  |  |  |
|  |  | **Amino Acid Metabolism** | | | | | | | | | | | | | | | | | | | | | | | |  |  |  |
| **Genus/Species**^1^ | *ppc* | *dtd* | *gcv* | | *amtB* | | *glt* | | *gadB* | *gadC* | *gabP* | | *gabT* | | *gabD* | | *glsA* | | | *ilvE* | | | *astB* | | | *asnB* |  |  |
| *Escherichia coli* |  |  |  | |  | |  | |  |  |  | |  | |  | |  | | |  | | |  | | |  |  |  |
| *Salmonella enterica* |  |  |  | |  | |  | |  |  |  | |  | |  | |  | | |  | | |  | | |  |  |  |
| *Enterococcus avium* |  |  |  | |  | |  | |  |  |  | |  | |  | |  | | |  | | |  | | |  |  |  |
| *Enterococcus faecalis* |  |  |  | |  | |  | |  |  |  | |  | |  | |  | | |  | | |  | | |  |  |  |
| *Enterococcus faecium* |  |  |  | |  | |  | |  |  |  | |  | |  | |  | | |  | | |  | | |  |  |  |
| *Lactobacillus crispatus* |  |  |  | |  | |  | |  |  |  | |  | |  | |  | | |  | | |  | | |  |  |  |
| *Clostridiales 02340*^2,7^ |  |  |  | |  | |  | |  |  |  | |  | |  | |  | | |  | | |  | | |  |  |  |
| *Anaerostipes caccae* |  |  |  | |  | |  | |  |  |  | |  | |  | |  | | |  | | |  | | |  |  |  |
| *Clostridioides difficile* |  |  |  | |  | |  | |  |  |  | |  | |  | |  | | |  | | |  | | |  |  |  |
| *Clostridium sp. C1*^7^ |  |  |  | |  | |  | |  |  |  | |  | |  | |  | | |  | | |  | | |  |  |  |
| *Clostridium sp. K10*^7^ |  |  |  | |  | |  | |  |  |  | |  | |  | |  | | |  | | |  | | |  |  |  |
| *Clostridium sp. M62/1*^7^ |  |  |  | |  | |  | |  |  |  | |  | |  | |  | | |  | | |  | | |  |  |  |
| *Eubacterium sp. c-25*^7^ |  |  |  | |  | |  | |  |  |  | |  | |  | |  | | |  | | |  | | |  |  |  |
| *Flavonifractor plautii* |  |  |  | |  | |  | |  |  |  | |  | |  | |  | | |  | | |  | | |  |  |  |
| *L. phocaeense* |  |  |  | |  | |  | |  |  |  | |  | |  | |  | | |  | | |  | | |  |  |  |
| *Oscillibacter sp. PEA192*^7^ |  |  |  | |  | |  | |  |  |  | |  | |  | |  | | |  | | |  | | |  |  |  |
| *Sellimonas intestinalis* |  |  |  | |  | |  | |  |  |  | |  | |  | |  | | |  | | |  | | |  |  |  |
| **Class** |  | **Code** | **Category** | | | | | | | | | | | | **Code** | |  | | |  | | |  | | |  |  |  |
| *γ-proteobacteria* |  |  | Universal Stress | | | | | | | | | | | |  | | **Total Genes =** | | | | | | | | | | 128 |  |
| *Bacteroidia* |  |  | Cold Stress | | | | | | | | | | | |  | | **Different Bacterial Species =** | | | | | | | | | | 49 |  |
| *Bacilli* |  |  | Envelop Stress | | | | | | | | | | | |  | |  | | |  | | |  | | |  |  |  |
| *Clostridia* |  |  | Oxidative Stress | | | | | | | | | | | |  | |  | | |  | | |  | | |  |  |  |
|  |  |  | Heat Shock | | | | | | | | | | | |  | |  | | |  | | |  | | |  |  |  |
|  |  |  | Starvation | | | | | | | | | | | |  | |  | | |  | | |  | | |  |  |  |
|  |  |  | Antimicrobials | | | | | | | | | | | |  | |  | | |  | | |  | | |  |  |  |
|  |  |  | Osmotic Shock | | | | | | | | | | | |  | |  | | |  | | |  | | |  |  |  |
|  |  |  | Carbohydrate Metabolism | | | | | | | | | | | |  | |  | | |  | | |  | | |  |  |  |
|  |  |  | Fermentation | | | | | | | | | | | |  | |  | | |  | | |  | | |  |  |  |
|  |  |  | Respiration | | | | | | | | | | | |  | |  | | |  | | |  | | |  |  |  |
|  |  |  | Amino Acid Metabolism | | | | | | | | | | | |  | |  | | |  | | |  | | |  |  |  |
|  |  |  | Miscellaneous | | | | | | | | | | | |  | |  | | |  | | |  | | |  |  |  |

Enzyme transcripts were identified in Bayesian network analysis of the cecal transcriptomes or Pearson/Spearman correlation coefficients between transcript abundance and *Salmonella* abundance (detailed in **Table 6**). ^1^ >98% nucleotide identity, >99% coverage by BLAST; ^2^ *Clostridiales* bacterium MGYG-HGUT-02340; ^3^ H_2_ producing hydrogenase; ^4^ Antimicrobial resistance gene cannot be ascribed to any one bacterial species due to its broad distribution (>99% nucleotide identity with 100% coverage by BLAST) but it has been reported in intestinal species listed; ^5^ yersiniabactin polyketide synthase HMWP1; ^6^ *ebgA*; α – Nicotinamidase (cysteine hydrolase); ^7^ Phylogenetically distinct and yet to be assigned new genus or species name; β - Pyridine nucleotide-disulphide oxidoreductase; γ – Rubrerythrin; δ – Type VI secretion system; ε – Toxin/antitoxin; ζ - Arabinogalactan endo-β-1,4-galactanase; η – β-glucuronidase. *A. rhamnosivorans*: *Anaerostipes rhamnosivorans*; *A. colihominis*: *Anaerotruncus* *colihominis*; *B. thetaiotaomicron*: *Bacteroides thetaiotaomicron*; *B. hydrogenotrophica*: *Blautia hydrogenotrophica*; *D. welbionis*: *Dysosmobacter welbionis*; *E. ramosum*: *Erysipelatoclostridium ramosum*; *I. butyriciproducens*: *Intestinimonas butyriciproducens*; *L. phocaeense*: *Lachnoclostridium phocaeense*; *L. plantarum*: *Lactiplantibacillus plantarum*; *L. asaccharolyticus*: *Lawsonibacter asaccharolyticus*; *L. vaginalis*: *Limosilactobacillus vaginalis*.
